# Face-to-face physical activity incorporated into dietary intervention for overweight/obesity in children and adolescents: a Bayesian network meta-analysis

**DOI:** 10.1186/s12916-022-02462-6

**Published:** 2022-09-02

**Authors:** Jing Hong Liang, Yu Zhao, Yi Can Chen, Nan Jiang, Shu Xin Zhang, Shan Huang, Aerziguli Kakaer, Ya Jun Chen

**Affiliations:** Department of Maternal and Child Health, School of Public Health, Sun Yat-sen University, No.74 Zhongshan 2nd Road, Yuexiu District, Guangzhou, 510080 People’s Republic of China

**Keywords:** Physical activity, Nutrition, Obesity, Children and adolescents, Bayesian network meta-analysis

## Abstract

**Background:**

Adolescent obesity has been reported to have deleterious consequences but is considered a promising modifiable risk factor. We aimed to investigate the optimal intervention for obese and overweight children and adolescents.

**Methods:**

We searched the Medline (PubMed, 1946–December 2020), PsycINFO (Ovid, 1927–December 2020), Cochrane library (1966–December 2020), Web of Science (1900–December 2020), Embase (1974–December 2020), CINAHL (1937–December 2020), Chinese Biomedical Literature (1978–December 2020), and ClinicalTrials.gov (December 2020) databases. We included randomized controlled trials (RCTs) reporting the association between various interventions and obese/overweight children and adolescents. The quality of the included studies was judged by two independent reviewers using the Cochrane Collaboration Risk of Bias Tool. A Bayesian network meta-analysis was conducted to summarize the comparative effectiveness of interventions based on several outcomes.

**Results:**

We included 118 RCTs comprising 71,064 participants in our analyses. Based on the outcome of the body mass index (BMI), face-to-face physical activity (FTF PA) combined with dietary intervention (DI) (mean difference [MD] = − 0.98; 95% credible interval [CrI] − 1.19, − 0.77), FTF multi-lifestyle intervention (MLI) (MD = − 0.95; 95% CrI − 1.14, − 0.75), and mobile health (MH)-delivered MLI (MD = − 0.87; 95% CrI − 1.63, − 0.09) showed significant benefits over the named control group (NCG). For the outcome of BMI z-score, FTF PA+DI (MD = − 0.10; 95% CrI − 0.15, − 0.04) and MH-delivered PA+DI (MD = − 0.09; 95% CrI − 0.14, − 0.04) were more effective than the NCG. Sensitivity analyses revealed similar findings after exclusion of studies with < 12-month and 24-month outcome assessments for the intervention, which indicated the results were stable.

**Conclusions:**

Based on limited quality evidence and limited direct evidence, our preliminary findings showed that FTF-PA+DI, FTF-MLI, and MH-delivered MLI improved the health-related parameters in obese adolescents, in comparison with NCG. Owing to the absence of strong, direct evidence of a significant difference between the various interventions for the four outcomes, we can only cautiously suggest that FTF-PA+DI is likely the most effective intervention.

**Supplementary Information:**

The online version contains supplementary material available at 10.1186/s12916-022-02462-6.

## Background

The continually high prevalence of childhood overweight and obesity worldwide has been well established [1, 2] and is associated with the rapid increase in numerous metabolic and cardiovascular complications such as hypertension, hyperlipidemia, and hyperglycemia [3–5]. The rising burden on families, medical systems, and even society, particularly in low-income countries [6], have tremendous long-term consequences globally.

Given the emergence of various adverse effects and the high risk of contraindications [7, 8] of metabolic surgery, an option that is only recommended for individuals with severe obesity (body mass index [BMI] > 32.5 and medication management), the efficacy of medication requires long-term administration and individuals’ weight would immediately rebound once they stop medications, such as leptin [9]. These measures are often used as alternatives to lifestyle-related interventions in improving the well-being of obese and overweight children and adolescents [10]. Previous quantitative reviews have reported the significant effectiveness of various treatments, including physical activity (PA), dietary intervention (DI), and multi-lifestyle intervention (MLI) [11–13] as well as whether different administration modes such as telemedicine technology or face-to-face (FTF) models [14, 15] influence the effectiveness of intervention. However, the evidence regarding effective interventions for obesity in children and adolescents remains fragmented and controversial. Many countries support various types of PA as the preferred intervention (such as aerobic exercise, resistance training, balance activities, and muscle-strengthening activities) [16, 17], as do the World Health Organization guidelines [18]. Some researchers have argued that other interventions combined with PAs are more effective than PA alone [19]. However, other studies give priority to PA, together with diet, as the essential component for both modulating childhood obesity and metabolic risk reduction. There have been inconsistent findings among previous meta-analyses, especially regarding school-based intervention (SBI) and the effect of intervention. For example, one study observed a moderate strength of evidence (SOE) regarding the effects of SBI [20], whereas a Cochrane review demonstrated strong evidence on the effects of SBI, particularly in children aged 6–12 [21]. Moreover, a high-quality meta-analysis involving 18 randomized controlled trials (RCTs) focused on SBI found that SBI did not reduce BMI among children [20]. Discrepancies among the preceding studies emphasize the need for an updated and comprehensive meta-analysis to gather reliable evidence. A limitation of the methodology used in conventional meta-analyses is that they can only evaluate the efficacy of separate interventions with the limited available evidence. No previous comprehensive studies have addressed which treatment is optimal for children and adolescents with obesity, and whether the treatment involves telemedicine technology. This is a complicated question to which the guidelines of many countries, and even evidence-based medical studies, have not yet offered any definitive answer. Consequently, the objective of our study was to investigate the optimal prevention/intervention strategy for obesity in children and adolescents using direct or indirect available evidence via a Bayesian network meta-analysis (NMA).

## Methods

### Protocol

Our paper adheres to the Preferred Reporting Items for Systematic Reviews and Meta-Analyses extension statement for systematic reviews incorporating network meta-analyses for health care (PRISMA-NMA) and the Cochrane Collaboration [22]. The PRISMA checklist is presented in Additional file 1: Appendix. S1. We have registered our study in the International Platform of Registered Systematic Review and Meta-analysis Protocols (INPLSY) with number 202120072 (see Additional file 2).

### Search strategy and study selection

With no limitation on language and publication data, two authors independently screened several databases including Medline (via PubMed), PsycINFO (via Ovid) Cochrane, Web of Science, Embase, CINAHL, the Chinese Biomedical Literature Database, and clinical trials (www.clinicaltrials.gov). The authors also used other meta-analysis search strategies to identify eligible RCTs investigating the association between various prevention and intervention strategies and obesity/overweight in children and adolescents, from their inception through December 1, 2020. The following Medical Subject Headings [MeSH] and keywords incorporating Boolean operators were applied: “children,” “adolescents,” “students,” “youth,” “treatment,” “diet,” “physical activity,” “telemedicine,” “healthy lifestyle,” “obesity,” “adiposity,” “overweight,” and “randomized controlled trials.”

Further searches were conducted manually, which included screening the bibliographies of relevant published systematic reviews or meta-analyses. The researchers conducted a review search of key journals and major conferences to identify eligible studies that may have been missed in the initial search. Details of the search strategy are presented in Additional file 1: Appendix. S2.

Based on the inclusion and exclusion criteria, two authors independently screened all titles, and the remaining abstracts were screened. All citations were imported and managed in Endnote software (Version X9; Thompson ISI Research Soft, Philadelphia, PA, USA), and duplicates were removed. Two authors screened the titles and abstracts independently, and the remaining studies were scrutinized in a full-text review by the same two investigators to ensure that potentially eligible articles were included. Disagreements during the process of literature search were resolved in discussion until reaching consensus or by involving an experienced expert in the last judgment.

### Inclusion and exclusion criteria

The inclusion criteria for publications were as follows: (1) interventions involving any type of PA (e.g., aerobic exercise, resistance training, endurance exercise), DI (e.g., very low-carbohydrate diet, very low-energy diet, low-fat diet), MLI, or any the abovementioned interventions in combination or as multiple components, whether delivered via mobile health (MH) technology or using an FTF approach; (2) studies recruiting participants who were children or adolescents aged 6–18 years using standardized diagnostic measures of obesity; (3) comparators were various interventions themselves or the named control group (NCG) alone, such as the wait-list control group or usual treatment; (4) children or adolescents with obesity or any prevalent subtype, assessed using measurable instruments or quantifiable indicators with quadratic transformation, such as BMI, BMI z-score, and waist circumference (WC); and (5) any type of RCT whether designed in parallel or cross-over settings. We placed no restriction regarding ethnicity, region, publication year, or language for the above items. Publications were excluded if they met the following criteria: (1) outcomes presented using biological indicators or other measures that cannot produce an intersecting endpoint in our analyses; (2) the NCG was combined with any existing intervention; and (3) non-randomized trials, such as protocols, population-based observational studies, or studies not containing the required data. Details of the inclusion and exclusion criteria are presented in Additional file 1: Appendix. S3.

### Outcome measure, data extraction, and quality appraisal

Pertinent information was collected by two independent authors based on a pre-defined data extraction strategy, which adhered to the Cochrane Consumers and Communication Review Group, that included the following: last name of the first author responsible for the study; year of publication; prespecified outcome of interest; and demographic characteristics such as age, sex ratio, and total participants [22].

The predetermined primary obesity-related outcomes were BMI and BMI *z*-score, and the secondary outcomes of interest were percent body fat (PBF) and WC. The odds ratio was used to compute the pooled effect sizes in each study with the random-effect model for dichotomous variables. Weight mean differences (WMDs) were pooled as the effect size of continuous data, derived by extracting the change from baseline to the last follow-up observation in the treatment group. We divided the study data if results were stratified by sex, grade, or other subgroups. A sequential approach was considered in our model, shown as follows: grade (children vs. adolescents), region (developed countries vs. developing and underdeveloped countries), publication year (≥ 2010 to < 2010), treatment cycle (duration ≥ 12 weeks vs. < 12 weeks), sex ratio (≥ 1 vs. < 1), total sample size (≥ 100 vs. < 100), intervention site (school vs. home), and treatment setting (group vs. individual). Discrepancies generated during the analyses were reconciled through consultation or judged by an experienced author.

The quality of the included studies was judged by two independent reviewers using the Cochrane Collaboration Risk of Bias Tool [23], which consists of seven items: random sequence generation, allocation concealment, blinding of participants and personnel, blinding of outcome assessors, incomplete outcome data, incomplete outcome reporting, and other bias. For selection bias, we considered studies clearly describing random sequence generation and specifying the method of allocation concealment to have a low risk of bias; otherwise, studies were considered to have a high risk of bias except when their methods on allocation concealment was not reported, and such studies were rated as unclear bias. Regarding performance and detection bias, this was mainly based on whether participants knew to which intervention they were assigned and whether caregivers and study coordinators were aware of which treatment participants had received. We considered studies missing long-term follow-up for individuals initially included and followed to have attrition bias. We appraised reporting bias based on whether the study reported insufficient available data and either differential or non-differential errors in measurement of the outcome data. For other potential biases, we rated studies through a full-text search for specific evidence that may lead to biased results, such as less rigorous study deigns or obvious inconsistency compared with previous studies. Low, unclear, and high risks of bias were rated as quality grades of the studies, respectively. We used the Grades of Recommendations Assessment, Development and Evaluation (GRADE) system to rate the findings, as outlined in the GRADE handbook [24–26]. Comparisons were initially rated as high-quality evidence (four plus:++++) and were downgraded accordingly, based on study limitations, imprecision, inconsistency, indirectness, and publication bias. We downgraded the study quality by one level in the study limitations item based on all the relevant ROB items including selection bias, performance bias, detection bias, attrition bias, reporting bias, and other bias. Situations such as comparisons failing to provide pertinent outcome data for all or most participants, the proportion of missing data at the endpoint being too high, or whether missing data were balanced between groups are also likely reasons for the downgrading [27]. For imprecision item, we downgraded it if the sample size was insufficient or if imprecise estimates of wide confidence interval were generated in this comparison. For the inconsistency item, if study heterogeneity, especially the local inconsistency was found between direct and indirect evidence among the comparisons, we downgraded it one level. The indirectness item was downgraded if there were heterogeneity observed based on four domains, namely, differences in populations, interventions, outcome measures, and indirect comparisons. When head-to-head comparisons are unavailable, the quality of evidence decreases. Additionally, we take into account the combined effect of the four types of indirectness [28].

As for publication bias item, we judged it through asymmetrical funnel plot.

Each item was rated as no downgrade, downgrade one level (serious), or downgrade two levels (very serious). Studies were upgraded for three reasons: a large magnitude of the effect (large: upgrade one level, + 1; very large: upgrade two level, + 2), a dose–response relationship (evidence of a gradient: upgrade one level, + 1), and attenuation by plausible confounding (would reduce a demonstrated effect: upgrade one level, + 1; would suggest a spurious effect if no effect was observed: upgrade two level, + 2).

After the above assessment, all comparisons were eventually rated as four levels of evidence, namely, high, moderate, low, or very low quality. GRADE assessment was done independently in duplicate by 2 investigators.

### Statistical analyses

For the conventional meta-analysis, in terms of heterogeneity, *I* ^2^ statistic values of 25%, 50%, and 75% indicated mild, moderate, and high heterogeneity, respectively, used to measure whether there was substantial heterogeneity [29].

A network plot was created to briefly summarize the entire acquirable evidence for each treatment. The sequence mentioned in the above analyses were implemented with Stata version 14.0 (StataCorp, College Station, TX, USA). For the effect sizes referring to successive results, we adopted the group (relevant) mean and standard deviation (SD) extracted from individual studies to compare each calculated WMD. The moving 95% credible interval (CrI) and pooled mean difference (MD) were computed as a reference to estimate pooled effect sizes separately [30]. If the included study did not provide the information needed in the analysis (such as mean, SD, or sample size), we computed other acquirable values, such as clarifying the SD (like standard error, confidence interval, or other statistical indicators) [31, 32].

Network transitivity is presented as the vital significant assumption in the NMA, the appraisal of which would further impact our analysis directly [33]. Thus, to ensure that multiple treatment comparisons were sufficiently similar, we estimated the transitivity by contrasting the clinical and methodological features, such as patient and experimental designs, in all the included studies [34]. With the Bayesian framework, the restrained maximum likelihood is presented as the estimation of parameters. The Bayesian hierarchical random effects were conducted to contrast diverse treatments. A connective network was generated and direct and indirect assessment was integrated using the method of multivariate meta-analysis to compare diverse treatments at the same time [35]. Three Markov chains in parallel were created randomly first, to simulate an exact appraisal in the statistical models. We generated 50,000 iterations per chain, referring to the period of burn-in, when the chain came to its eventual distribution. To make the beginning value deviate at a minimum, the former 10,000 iterations were given up [36, 37]. The model convergence was estimated using the Brooks–Gelman–Rubin diagnostic, in which the historical trajectory was observed directly combining the trace plot and density plot [38]. As an appraisable likelihood that gave a grade of treatments in the target, the surface under the cumulative ranking curve (SUCRA) summarized individual treatments by providing a brief numerical statistic cumulative ranking probability plot. If the value of SUCRA is higher, it is more probable that the provided treatment ranks or takes effect at the top; on the contrary, the SUCRA value is zero, the treatment has the worst result [39]. The “node-splitting” technique was used to clarify the possibility of an underlying source difference contrasting the direct and indirect evidence from all networks (consistency appears when the *p*-value surpasses 0.05) [40]. For ensuring the quality of included studies and evaluating the stability of the results, further sensitivity analyses were conducted with the included studies limited to trials whose outcome assessments of the intervention were more than 12 months and 24 months, respectively. The Bayesian NMA hierarchical model was implemented in OpenBUGS version 14 (OpenBUGS code shown in Additional file 1: Appendix. S4). Finally, based on several variables of interest, planned random-effect subgroup analyses were conducted to ensure the robustness of the summarized effect size, and all of them were judged as preestablished concomitant variables.

## Results

### Participants and study characteristics

**Fig. 1:**
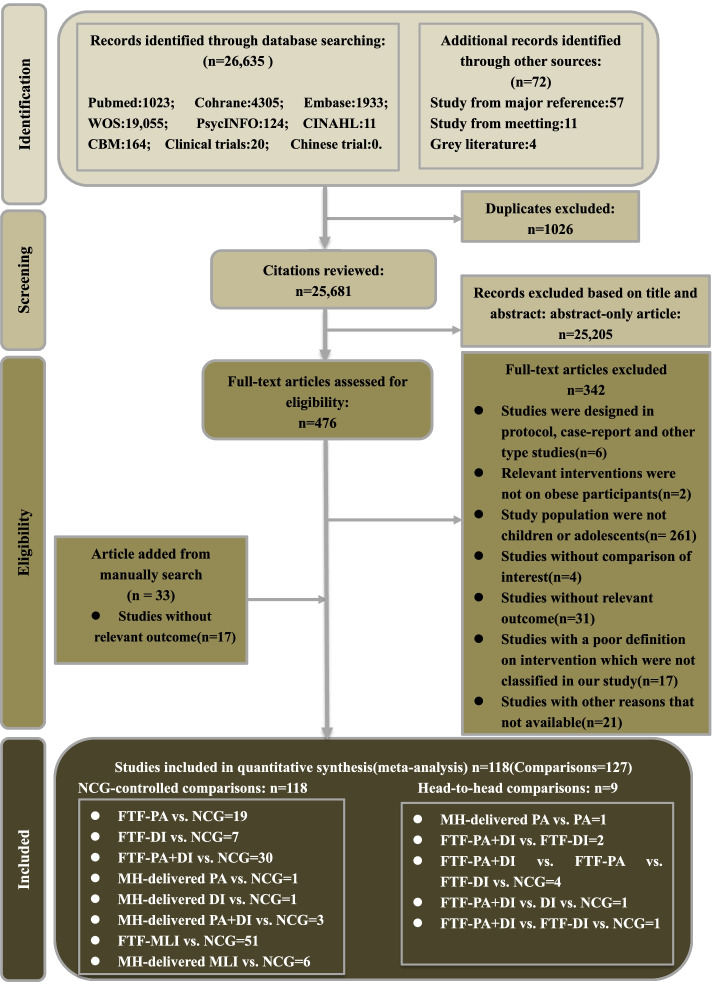
Literature review flowchart. DI, dietary intervention; FTF, face-to-face; MH-delivered, mobile health-based; MLI, multi-lifestyle intervention; NCG, named control group; PA, physical activity; WOS, Web of Science

The PRISMA-NMA flow chart for study literature selection is presented in Fig. 1. The initial database search yielded a total of 26,635 records, and another 72 were identified in a manual search; among the total, there were 1026 duplicates. After screening of the titles and abstracts, 25,205 studies were excluded; the remaining 476 potentially eligible studies were included in the full-text review. We deemed 102 trials to be eligible, according to the preset inclusion criteria. A total of 342 trials were excluded for various reasons (23 did not explicitly provide the key information, 6 were not RCTs or data appeared elsewhere, participants in 261 studies were not primarily children and adolescents, 31 studies were missing relevant outcomes, and 21 did not present the available data). We further retrieved relevant trials in a manual search, and 16 studies met our inclusion criteria. Finally, 118 trials were included in our analysis. One-hundred and ten studies were unique, while the remaining eight were by how they reported results, by sex, grade, or other subgroups.

The 118 included studies comprised 71,064 participants who were randomly assigned to either the intervention group (*N* = 38,500) or control group (*N* = 32,564). The median therapeutic duration across 118 RCTs was 6 months and the participants age ranged from 6.03 to 16.14 years (median age = 10.90 years). Regarding regions, 43 studies were conducted in the USA, 36 were in Europe, and the remaining 21 and 18 studies were conducted in Asia and other regions, respectively. Fifty-five trials described the setting of the intervention; 39 of these were in a group setting and 16 were conducted individually. In most included trials, the sex ratio was > 1 (*N* = 62, 48.4%); a total of 13 studies did not report participants’ sex. All included studies were published from 2002 to 2019 and were written in English. Table 1 summarizes the main information of the 118 included studies. Additional file 3: Tab. S1 gives other details regarding demographic characteristics of the included 118 studies (References were shown in Additional file 1: Appendix. S5).

**Table 1:** Demographic characteristics of the included studies and their participants

Characteristics of the 118 included studies	No (%) of studies
Publication year	
2000-2005	8(6.8%)
2006-2010	39(33.1%)
2011-2015	55(46.6%)
2016-	16(13.6%)
Treatment duration(Months)	
1 < M ≤ 3	27(22.9%)
3 < M ≤ 6	35(29.7%)
6 < M ≤ 12	42(35.6%)
M ≥ 12	14(11.9%)
Geographical region	
America	43(36.4%)
Europe	36(30.1%)
Asia	21(17.8%)
Other region	18(15.3%)
Type of obesity	
Obesity	52(44.1%)
Overweight	16(13.6%)
Obesity&Overweight	39(33.1%)
Unclear	11(9.3%)
Outcomes	
BMI	102(40.0%)
BMI Z-score	55(21.6%)
WC	51(20.0%)
PBF	47(18.4%)
Type of RCT	
Cluster	33(28.0%)
Parallel	18(15.3%)
NR	67(56.8%)
Total sample size(N)	
1-100	50(39.1%)
101-500	44(34.4%)
501-1000	16(12.5%)
1001-	18(14.1%)
Baseline age(years)	
Children(6-12)	69(53.9%)
Adolescent(13-18)	27(21.1%)
Both	28(21.9%)
NR	4(3.1%)
Proportion boys(%)	
≥50	62(48.4%)
＜50	53(41.4%)
NR	13(10.1%)
Involvement of center(%)	
Home	65(50.8%)
School	20(15.6%)
Community	10(7.8%)
Combination	3(2.3%)
Unclear	30(23.4%)
Setting	
Group	39(30.5%)
Individual	16(12.5%)
NR	73(57.0%)
Race	
White	4(3.4%)
Black	5(4.2%)
Other/Asia/Yellow	2(1.7%)
Multi-race	28(23.7%)
NR	79(66.9%)

BMI, Body mass index; M, Month; NR, Not reported; PBF, Percent body fat; RCT, Randomized controlled trial; WC, Waist circumference

### Quality of the included studies

Most trials tended to have a low risk of bias with respect to randomization (*N* = 114, 96.61%); 37.30% were deemed to have selection bias (*N* = 44), 37.29% performance bias [blinding of participants and personnel, (*N* = 44)], 56.78% attrition bias (*N* = 67), 73.73% reporting bias (*N* = 87), and 25.42% had other bias (*N* = 30). Concerns were mainly in the domain of selection bias (*N* = 14, 11.86%) and other bias (*N* = 13, 11.02%). The overall and individual risk of bias are shown in Additional file 4: Fig. S1, Additional file 4: Fig. S2 and Additional file 3: Tab. S13. The GRADE level of evidence for the primary outcome was rated very low to low quality, shown in Additional file 3: Tab. S9-12.

### Network plot

**Fig. 2:**
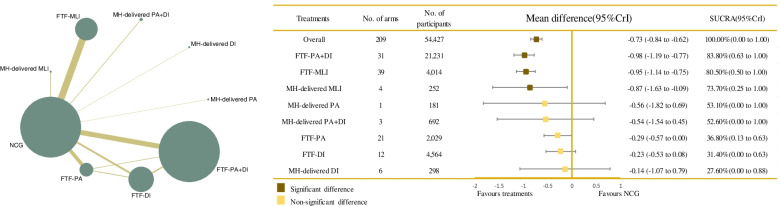
Network plot of all the trials based on the outcome of BMI, and the efficacy of varied treatments compared with named control group. Each node represented a treatment and its size depended on the number of patients that is directly examined. The nodes were joined by different thickness lines which generated to show whether there existed a direct relationship between treatments and the thickness was weighted according to the available direct evidence between them. CrIs, credible intervals; DI, dietary intervention; FTF, face-to-face; MH-delivered, mobile health-based; MLI, multi-lifestyle intervention; NCG, named control group; NR, not reported; PA, physical activity; SUCRA, the surface under the cumulative ranking curve

**Fig. 3:**
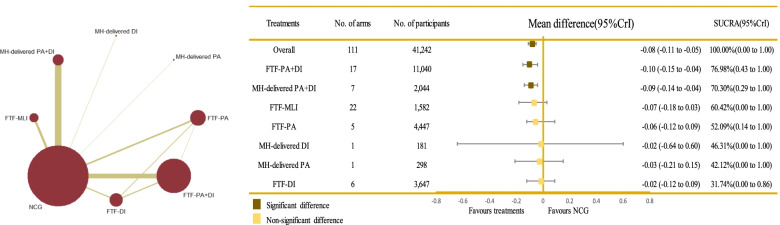
Network plot of all the trials based on the outcome of BMI *z*-score, and the efficacy of varied treatments compared with named control group. Each node represented a treatment and its size depended on the number of patients that is directly examined. The nodes were joined by different thickness lines which generated to show whether there existed a direct relationship between treatments and the thickness was weighted according to the available direct evidence between them. CrIs, credible intervals; DI, dietary intervention; FTF, face-to-face; MH-delivered, mobile health-based; MLI, multi-lifestyle intervention; NCG, named control group; NR, not reported; PA, physical activity; SUCRA, the surface under the cumulative ranking curve

The network evidence of the included trials based on BMI outcomes, to evaluate significant relationships between each arm, is graphically presented in Fig. 2. The size of the nodes represented the total sample size of the intervention, which indicated that FTF-PA+DI had the most participants (*N* = 21,231, 39.0%), and MH-delivered DI had the fewest (*N* = 298, 0.5%). Moreover, the thickness of the lines between interventions related to the number of comparisons showed that direct evidence between FTF-MLI and the NCG was greater than for other comparisons. The remaining network plot regarding BMI z-score, WC, and PBF are given in Fig. 3 and Additional file 4: Fig. S3-4.

### Primary outcome

Based on the outcome of BMI, our results showed that when compared with the NCG, FTF-PA+DI (MD = − 0.98; 95% CrI − 1.19, − 0.77), FTF-MLI (MD = − 0.95; 95% CrI − 1.14, − 0.75), and MH-delivered MLI (MD = − 0.87; 95% CrI − 1.63, − 0.09) all showed significant effectiveness. Specifically, the administration of FTF-PA+DI was associated with significantly greater effectiveness than FTF-PA alone (MD = − 0.69; 95% CrI − 1.04, − 0.36) and FTF-DI alone (MD = − 0.75; 95% CrI − 1.10, − 0.41). Equally remarkable improvement was observed with FTF-MLI treatment, which was more effective than FTF-PA (MD = − 0.66; 95% CrI − 1.01, − 0.31) and FTF-DI (MD = − 0.72; 95% CrI − 1.08, − 0.36). No significant differences were noted for the remaining treatments. Details are shown in Fig. 2 and Table 2. FTF-PA+DI (SUCRA 83.84%; 95% CrI 0.63, 1.00) possessed the greatest likelihood of being the best intervention for BMI, along with the suboptimal intervention FTF-MLI (SUCRA 80.54%; 95% CrI 0.50, 1.00], followed by MH-delivered MLI (SUCRA 73.67%; 95% CrI 0.25, 1.00]; NCG had the lowest SUCRA (SUCRA 10.35%; 95% CrI 0.00, 0.38], as shown in Table 2, Fig. 2, and Additional file 4: Fig. S9). A comparison-adjusted funnel plot failed to provide evidence of obvious publication bias (Additional file 4: Fig. S5). No obvious heterogeneity was detected for BMI (global heterogeneity, 16.51% [pairwise] and 2.75% [consistency]; SD = 0.09). There were no statistically significant inconsistencies across the direct and indirect evidence among interventions (FTF-PA vs. FTF-DI *p*-value = 0.778, FTF-PA+DI vs. FTF-PA *p*-value = 0.485, FTF-PA+DI vs. FTF-DI *p*-value = 0.413).

**Table 2:**
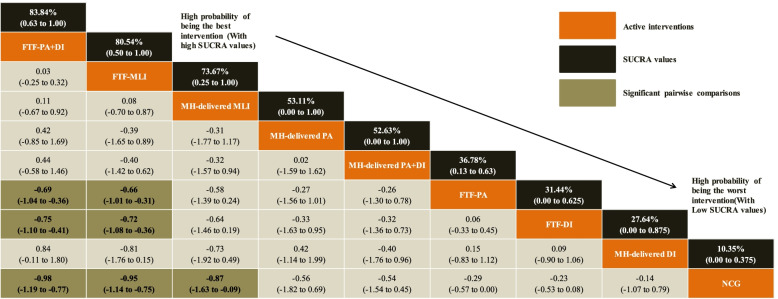
Relative effect sizes of treatments efficacy at post-treatment based on network meta-analysis (BMI outcome)

Treatments are orders in the rank of their chance of being the best treatment. Numbers in black boxes are SUCRA (the surface under the cumulative ranking curve) values and their CrIs (credible intervals), which represented the rank of the treatment. Significant pairwise comparisons are highlighted in grey and in bold. For efficacy in post-treatment, standardized mean differences (MDs) less than 0 favor the column-defining treatment. DI, Dietary intervention; FTF, Face-to-face; MH-delivered, Mobile health-delivered; MLI, Muti-lifestyle intervention; NCG, Named control group; PA, Physical activity

In terms of the outcome of BMI *z*-score, FTF-PA+DI (MD = − 0.10; 95% CrI − 0.15, − 0.04) and MH-delivered PA+DI (MD = − 0.09; 95% CrI − 0.14, − 0.04) were more effective than the NCG. No significant differences were detected between treatments, as shown in Fig. 3 and Table 3. FTF-PA+DI (SUCRA 76.98%; 95% CrI 0.43, 1.00) was clearly ranked best for the BMI z-score outcome, followed by MH-delivered PA+DI (SUCRA 70.30%; 95% CrI 0.29, 1.00) and FTF-MLI (SUCRA 60.42%; 95% CrI 0.00, 1.00), shown in Table 3, Fig. 3 and Additional file 4: Fig. S10). Mild heterogeneity was detected for BMI *z*-score (global heterogeneity, 34.28% [pairwise] and 21.01% [consistency]; SD = 0.49]. The comparison-adjusted funnel plots for BMI z-score outcome is presented in Additional file 4: Fig. S6. There was a statistically significant inconsistency between FTF-PA+DI vs. FTF-PA (*p*-value < 0.001); the direct and indirect evidence for other treatments showed consistent results (FTF-PA vs. FTF-DI *p*-value = 0.106, FTF-PA+DI vs. FTF-DI *p*-value = 0.108).

**Table 3:**
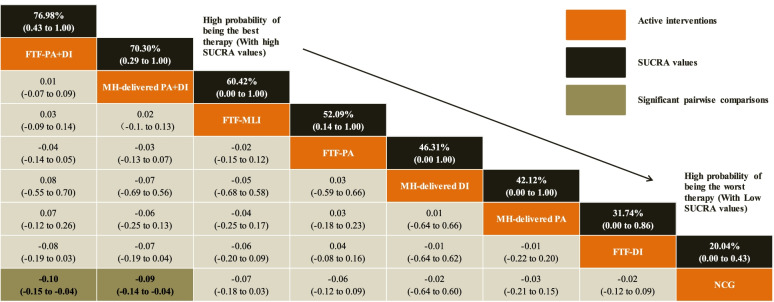
Relative effect sizes of treatments efficacy at post-treatment based on network meta-analysis (BMI *Z*-score outcome)

Treatments are orders in the rank of their chance of being the best treatment. Numbers in black boxes are SUCRA (the surface under the cumulative ranking curve) values and their CrIs (credible intervals), which represented the rank of the treatment. Significant pairwise comparisons are highlighted in grey and in bold. For efficacy in post-treatment, standardized mean differences (MDs) less than 0 favor the column-defining treatment. DI, dietary intervention; FTF, face-to-face; MH-delivered, mobile health-delivered; MLI, multi-lifestyle intervention; NCG, named control group; PA, physical activity

Regarding the WC outcome, FTF-PA+DI (MD = − 1.49; 95% CrI − 1.97, − 1.00) and FTF-MLI (MD = − 1.14; 95% CrI − 1.63, − 0.64) were significantly better than the NCG. FTF-PA+DI appeared to be significantly more effective than FTF-PA alone (MD = − 1.12; 95% CrI − 1.93, − 0.29), shown in Additional file 4: Fig. S3, Additional file 3: Tab. S2(A) and Additional file 4: Fig. S11. The same results were found for PBF, in that FTF-PA+DI (MD = − 0.98; 95% CrI − 1.41, − 0.64), MH-delivered PA+DI (MD = − 0.68; 95% CrI − 1.25, − 0.14), and FTF-MLI (MD = − 1.53; 95% CrI − 3.06, − 0.003) were significantly better than NCG. FTF-PA+DI appeared to be significantly more effective than FTF-PA alone (MD = − 0.71; 95% CrI − 1.23, − 0.27), shown in Additional file 4: Fig. S4, Additional file 3: Tab. S2(B) and Additional file 4: Fig. S12. No heterogeneity was observed for WC (global heterogeneity, 14.20% [pairwise] and 0% [consistency]; SD = 0.25) and mild heterogeneity was observed for PBF (global heterogeneity, 18.46% [pairwise] and 7.23% 0% [consistency]; SD = 0.69]. No indication of inconsistency between direct and indirect evidence was found for the outcomes of WC (FTF-PA vs. FTF-DI *p*-value = 0.901, FTF-PA+DI vs. FTF-DI *p*-value = 0.992) and PBF (FTF-PA vs. FTF-DI *p*-value = 0.601, FTF-PA vs. MH-delivered PA *p*-value = 0.900, FTF-PA+DI vs. FTF-DI *p*-value = 0.325). The comparison-adjusted funnel plots for all secondary outcomes are presented in Additional file 4: Fig. S7-8.

Based on the outcome of BMI, subgroup analyses sequentially showed a consistent trend for most of them; there was no significant difference between subgroup items. Details are presented in Additional file 3: Tab. S3-6. Furthermore, the local inconsistency plots for all outcomes are presented in Additional file 4: Fig. S13-16.

Based on the primary outcome, we noted that the results of sensitivity analyses excluding the trials whose interventions outcome assessments were < 12 months and 24 months, respectively, were consistent with the former results that FTF-PA+DI (12 months: MD = − 0.30; 95% CrI − 0.94, 0.34, 24 months: MD = − 1.44; 95% CrI − 2.18, − 0.71) had the greatest likelihood of improving obesity/overweight among children and adolescents, followed by FTF-MLI (12 months: MD = − 0.30; 95% CrI − 1.74, 1.14 and 24 months: MD = − 0.78; 95% CrI − 2.00, 0.43). Details of the sensitivity analyses are shown in Additional file 3: Tab. S7-8, and Additional file 4: Fig. S17-37.

## Discussion

Our comprehensive and systematic NMA was implemented by updating the currently available data regarding multiple interventions for obese children and adolescents. Our study comprised 118 RCTs comparing eight treatment arms with 71,064 participants and concluded that FTF-PA+DI, FTF-MLI, and MH-delivered MLI were statistically beneficial to obese children and adolescents compared with the NCG. Because limited quality evidence and direct evidence were highly represented among the included studies, our findings should be interpreted with caution in terms of FTF-PA+DI appearing to be the optimal therapeutic strategy for achieving weight loss, based on the four outcomes investigated.

Our findings were inconsistent with a previous quantitative analysis published in 2020, which assessed the efficacy of diet, exercise, and lifestyle intervention for obese youth, based on limited outcomes with only 24 RCTs. That study concluded that there was insufficient indication that exercise exhibited the highest likelihood for effectiveness; exercise and diet were ranked third, which was inferior to the dietary intervention [41]. Our study has further enlarged the evidence regarding intervention strategies by conducting an exhaustive search of 11 databases through December 2020. Our search identified 118 RCTs among 71,064 participants with more reliable outcomes for obesity with high precision for estimates. Our analyses indicated that FTF-PA+DI was significantly more effective for obesity among children and adolescents. The divergence in study findings is probably derived from differences in the inclusion and exclusion criteria among the included studies, the search strategies, as well as the methods of analyses. The Bayesian framework has relative advantages over a frequency method in providing a flexible and efficient modeling instrument by estimating a posterior probability and also controlling various types of bias produced in the iteration process. Thus, the effect size is more precise and stable based on the Bayesian model. Previously published studies on the effectiveness of PA combined with DI in obese children [42, 43] have proposed the most sustainable strategy for improving adiposity in children. Our findings were consistent with previous evidence that such interventions are likely to be more effective for younger children and girls [44]. This is perhaps because girls commonly reach physical maturation earlier than boys, and childhood may be a high-plasticity age window for the development of obesity/overweight, fora preschool children in particular [44, 45]. However, the effectiveness of a single intervention (PA or DI) was not significantly effective in reducing BMI (FTF-PA: MD = − 0.29; 95% CrI –0.57, 0.002 and FTF-DI: MD = − 0.23; 95% CrI –0.53, 0.08). A potential explanation for this finding is that confounding factors, such as the dose of intervention, different interventions, and residual confounding from other unmeasured factors counteracted the efficacy of single intervention. Another contributing factors may be attributed to the complexity of the Bayesian approach, where some single interventions may result in a significant (or non-significant) effect for obese/overweight youths based on the original data; however, this interpretation is weakened when considering other interventions in a comparative analysis, as we illustrate below in the limitations.

The intensity and frequency of the defined PA varied appreciably; therefore, we did not classify subtypes of intervention. For example, the intensity of PA differed, and was commonly presented as low PA, moderate PA, or moderate-to-vigorous-intensity PA; the form of PA could usually characterized as aerobic PA, resistance PA, muscular PA, endurance PA, a reduction in sedentary behavior, or indirect substitution of PA. Thus, it was difficult to categorize the included interventions by subtype, especially a combinations of the different interventions. However, increasing PA was a common principle for reducing body fat [46]. Furthermore, reducing screen time, sitting time, or other sedentary behaviors with defined metabolic equivalent (MET) units < 1.5 were also potentially modifiable risk factors or indirect treatments for obese children, apart from PA [46]. Ideally, reliable evidence and guidelines highlight the amount of time spent being sedentary should be limited, particularly the amount of recreational screen time [18, 47].

BMI is a well-recognized proxy for general child adiposity and is one of the best measures of change in adiposity in growing children [48]. However, this indirect measure of obesity is not a fitting indicator for evaluating healthy people [20] compared with BMI *z*-score, an SD score [49]; BMI *z*-score is an optimal measure because it has greater validity and acceptability than BMI for evaluating overweight or obesity in children and adolescents [50, 51]. Prior studies recommend that a reduction in BMI *z*-score of 0.15–0.25 is associated with fewer cardiovascular and metabolic risk factors in children; a reduction in the range of 0.20–0.25 appears to represent a clinically meaningful change across an important threshold [52, 53]. Nevertheless, in our analysis of BMI *z*-score, a smaller value (Fig. 3 and Table 3) was detected than the recommendations, which may arise from the different study design between the recommendations and our study, especially extraction of the outcome point. For example, the lack of long-term follow-up is a serious limitation because of the propensity for people to regain lost weight. The recommendations are for a minimum follow-up of more than 12 months to increase the overall quality of the study and limit the inclusion of lower quality research. Thus, to comprehensively evaluate the efficacy of the multiple interventions, we used BMI *z*-score, WC, and PBF as measures of adiposity rather than relying solely on BMI. From the outcomes mentioned above, we obtained the consistent conclusion that FTF-PA combined with DI showed the greatest likelihood of being effective in reducing weight among obese children.

With the increasing popularity of electronic devices among young, mobile health intervention, namely, incorporating medical technology into telecommunication, is used to intervene obesity among individuals [54, 55]. Delivery of intervention remotely via MH technology is a cost-effective approach [56]; however, according to our findings, its effectiveness lagged behind that of FTF interventions. Several possibilities for this are worth noting. Self-monitoring without parental involvement may introduce incorrect information that gives rise to reporting bias. Remote monitoring is mainly conducted with individual settings, which weakens the initiative and practicability for individual participants, compared with a group setting. However, evidence suggests that MH-delivered interventions are more likely to lead to attracting and retaining a number of participants because these can broaden the reach of intervention platforms beyond the end of FTF interventions [57, 58]. Well-documented theories suggest that family involvement is a pivotal component in the treatment for children and adolescents with obesity, which has been widely advocated [59–61]. Most of our included studies involved family components, commonly involving parental education [62–64]. One study suggested that nearly all effective treatments included a family session, especially in participants below 12 years of age [65]. Several meta-analyses highlighted that school-based interventions have a crucial role in primary strategies to prevent childhood obesity; this means that the results of intervention may be influenced by the properties of the community where the intervention is applied [66]. Hence, we conducted subgroup analysis based on the variable of whether studies were conducted in school or at home, to ensure the robustness of the summarized effect size. We found several significant differences in the school item, whereas no statistical significance was observed in the family item, which may imply that interventions carried out in school are more effective than those performed at home. PA as a common recommendation in the guidelines of numerous countries [16, 17, 47] is not only beneficial to body fitness but can also improve various physical aspects including vision, executive function, and mental diseases [67, 68]. We excluded pharmacological interventions, as these have some potential adverse effects. Numerous clinical trials are seeking to identify other potential drug candidates to avert these adverse reactions [69, 70].

In investigating whether the outcome assessments of intervention duration influence the effects of intervention on obesity/overweight in children and adolescents, sensitivity analyses were performed, which yielded similar results based on the two items (outcome assessments > 12 months and 24 months), indicating that FTF-PA+DI might be the best strategy for obese/overweight youth and that our findings were stable and reliable. Thus, we may conclude that there is no difference between outcome assessment of treatment duration groups. However, differences between various intervention based on treatment duration did not reach statistical significance, which requires further validation in high-quality and long-term RCTs.

### Strengths

From a methodological point of view, Bayesian NMA can overcome the challenges of conventional meta-analysis, which only analyzes the efficacy of one intervention against one control group. In contrast, Bayesian NMA provides an overview by comparing two or more interventions simultaneously and ranking them, even if direct evidence is absent between them. Within the Bayesian framework, all parameters are tested as random variables, meaning that Bayesian NMA has a categorical advantage over frequentist methodology because of its capability to outline comparisons between various treatments concurrently. Each of these was obtained using the maximum a posteriori, which allows greater flexibility to use complex models and produces a relatively scientific interpretation in terms of causal relationships. Emerging evidence has revealed that the prevalence of overweight has remained somewhat steady [71]. However, there has been a growth in the number of children with severe obesity [72]. With a central role in governing the persistently high levels of obesity during childhood globally, current evidence-based strategies have offered convenient, reliable, and acceptable alternatives for schools, clinicians, and service providers choosing among various interventions. In our literature search, no publication period or language restriction was imposed, and we extend the source of studies to include more trials, thereby enhancing the evidence and making our findings very reliable and accurate in terms of causal relationships.

### Limitations

The limitations of our study should be discussed. First, the quality of the included studies is a weakness worth underscoring. Although most included trials reported their randomization approaches, other clear biases were also generated. The participants in trial were hard to blind, mainly owing to their recent behavior such as dietary intake, exercise engagement or their weight trajectory, which can factor into decision-making in intervention adjustment. Another potential bias was that a proportion of trials had not registered their study on the authorized website. Such difference may influence their quality. Moreover, the Bayesian approach is complicated in that the results of various interventions are sometimes hard to explain. For example, the FTF PA+DI is deemed the optimal intervention based on several outcomes, whereas single FTF-PA or FTF-DI was not significantly effective. This might be ascribed to the quality of the included studies and that FTF-PA+DI, as a comprehensive intervention, could more easily be influenced by numerous confounding effects (FTF-PA+DI had a relatively large sample population compared with the total sample), which may make our initial results produce bias elsewhere than a single intervention. Furthermore, our study was concluded 1 year after the initial search and some relevant trials may have been published during this period. Such publications could affect the validity of our results. Thus, our findings should be interpreted with caution and updated evidence should be included in further research to confirm our findings. It is also noted that the inclusion and exclusion criteria in our study were consistent with a previous protocol, although we added more analyses, such as subgroup and sensitivity analyses, which differed from the previous protocol. Finally, the heterogeneity between the included studies can partly be explained by methodological differences, especially the study design, such as differences in the primary outcome, intervention, and participants.

## Conclusions

Based on a relatively sufficient number of available trials, we found that FTF-PA+DI, FTF-MLI, and MH-delivered MLI had beneficial contributions to managing obesity among children and adolescents. However, with the limited quality of evidence and limited direct evidence, we suggest cautious interpretation of our finding that FTF-PA+DI is likely the best choice among the interventions analyzed. Future high-quality trials should consider including PA and DI, with a focus on PA+DI in particular, to establish an evidence base for intervention strategies for children and adolescents with obesity.

## Supplementary Information

**Additional file 1: Appendix. S1** Inclusion and Exclusion criteria; Appendix. S2 Main analysis OpenBUGS code; Appendix. S3 PRISMA NMA Checklist; Appendix. S4 Search strategies for all databases; Appendix. S5 The references for 118 included studies.

**Additional file 2.** INPLASY Protocol.

**Additional file 3: Table S1**. Baseline characteristics of the 118 included trials; **Table. S2**. Relative effect sizes of treatments efficacy at post-treatment based on network meta-analysis (WC and PBF outcomes); **Table. S3-6.** Subgroup analyses for varied treatments based on the outcome of BMI; **Table. S7-8.** Sensitivity analyses of outcomes assessment of interventions duration for varied treatments based on the outcome of BMI; **Table. S9-12.** The confidence in MD for mean overall change in four outcomes by GRADE system; **Table. S13.** Risk of bias for individual study.

**Additional file 4: Figure. S1-2** Risk of Bias graph; **Figure. S3-4** Network of evidence of all the trials based on the outcomes of WC and PBF, and the Efficacy of varied treatments compared with named control group; **Figure. S5-8** Funnel plots based on the four outcomes; **Figure. S9-12** SUCRA plots based on the four outcomes; **Figure. S13-16** Inconsistency plots based on the four outcomes; **Figure. S17-23** Network evidence of sensitivity analysis based on the four outcomes(12 and 24 months); **Figure. S24-30** SUCRA plots of sensitivity analysis based on the four outcomes(12 and 24 months); **Figure. S31-37** Funnel plots of sensitivity analysis based on the four outcomes(12 and 24 months).

## Data Availability

No ethical approval or patient consent was required in that all analyses were conducted based on previously published studies.

## Abbreviations

AEs: Adverse effects
BMI: Body mass index
CI: Confidence interval
CrI: Credible Interval
DI: Dietary intervention
FTF: Face-to-face
GRADE: Grades of Recommendations Assessment Development and Evaluation
MD: Mean difference
MeSH: Medical subject headings
MH-delivered: Mobile health-delivered
MLI: Multi-lifestyle intervention
MVPA: Moderate-to-vigorous-intensity PA
NCG: Named control group
NMA: Network meta-analysis
RCTs: Randomized controlled trials
ROB: Risk of bias
SBI: School-based intervention
SDs: Standard deviation
SOE: Strength of evidence
SUCRA: Surface under the cumulative ranking curve
PBF: Percent body fat
PRISMA-NMA: Preferred Reporting Items for Systematic Reviews and Meta-Analyses extension statement for systematic reviews incorporating network meta-analyses for healthcare
PA: Physical activity
WC: Waist circumference
WMDs: Weight mean differences
