# Supplementary material for: Face-to-face physical activity incorporated into dietary intervention for overweight/obesity in children and adolescents: a Bayesian network meta-analysis
Source: BMC Med. 2022 Sep 2;20:325. doi: 10.1186/s12916-022-02462-6 (PMC9438135; doi:10.1186/s12916-022-02462-6)
Supplement: Supplementary file 1 — Additional file 1: Appendix. S1 Inclusion and Exclusion criteria; Appendix. S2 Main analysis OpenBUGS code; Appendix. S3 PRISMA NMA Checklist; Appendix. S4 Search strategies for all databases; Appendix. S5 The references for 118 included studies. [file 12916_2022_2462_MOESM1_ESM.docx]

**Appendix. S1 PRISMA NMA Checklist of Items to Include When Reporting A Systematic Review Involving a Network Meta-analysis**

| **Section/Topic** | **Item**  **#** | **Checklist Item** | **Reported on Page #** |
| --- | --- | --- | --- |
| **TITLE** | | |  |
| Title | 1 | Identify the report as a systematic review *incorporating a network meta-analysis (or related form of meta-analysis).* | **Page. 1**  **And Title page** |
| **ABSTRACT** |  |  | **Page. 2-3** |
| Structured summary | 2 | Provide a structured summary including, as applicable:  **Background:** main objectives  **Methods:** data sources; study eligibility criteria, participants, and interventions; study appraisal; and *synthesis methods, such as network meta-analysis.*  **Results:** number of studies and participants identified; summary estimates with corresponding confidence/credible intervals; *treatment rankings may also be discussed. Authors may choose to summarize pairwise comparisons against a chosen treatment included in their analyses for brevity.*  **Discussion/Conclusions:** limitations; conclusions and implications of findings.  **Other:** primary source of funding; systematic review registration number with registry name. |  |
| **INTRODUCTION** |  |  |  |
| Rationale | 3 | Describe the rationale for the review in the context of what is already known*, including mention of why a network meta- analysis has been conducted.* | **Page. 3-4** |
| Objectives | 4 | Provide an explicit statement of questions being addressed, with reference to participants, interventions, comparisons, outcomes, and study design (PICOS). | **Page. 4** |
| **METHODS** |  |  |  |
| Protocol and registration | 5 | Indicate whether a review protocol exists and if and where it can be accessed (e.g., Web address); and, if available, provide registration information, including registration number. | **INPLASY**  **202120072**  **Additional file 4** |
| Eligibility criteria | 6 | Specify study characteristics (e.g., PICOS, length of follow-up) and report characteristics (e.g., years considered, language, publication status) used as criteria for eligibility, giving rationale. *Clearly describe eligible treatments included in the treatment network, and note whether any have been clustered or merged into the same node (with justification).* | **Page. 5-6**  **And Additional file 1: Appendix. S1.** |
| Information sources | 7 | Describe all information sources (e.g., databases with dates of coverage, contact with study authors to identify additional studies) in the search and date last searched. | **Page. 5** |
| Search | 8 | Present full electronic search strategy for at least one database, including any limits used, such that it could be repeated. | **Additional file 1: Appendix. S4** |
| Study selection | 9 | State the process for selecting studies (i.e., screening, eligibility, included in systematic review, and, if applicable, | **Page. 5** |

| included in the meta-analysis). | | |  |
| --- | --- | --- | --- |
| Data collection process | 10 | Describe method of data extraction from reports (e.g., piloted forms, independently, in duplicate) and any processes for obtaining and confirming data from investigators. | **Page. 5** |
| Data items | 11 | List and define all variables for which data were sought (e.g., PICOS, funding sources) and any assumptions and simplifications made. | **Page.5-6** |
| **Geometry of the network** | **S1** | Describe methods used to explore the geometry of the treatment network under study and potential biases related to it. This should include how the evidence base has been graphically summarized for presentation, and what characteristics were compiled and used to describe the evidence base to readers. | **Page. 8** |
| Risk of bias within individual studies | 12 | Describe methods used for assessing risk of bias of individual studies (including specification of whether this was done at the study or outcome level), and how this information is to be used in any data synthesis. | **Page. 7-8** |
| Summary measures | 13 | State the principal summary measures (e.g., risk ratio, difference in means). *Also describe the use of additional summary measures assessed, such as treatment rankings and surface under the cumulative ranking curve (SUCRA) values, as well as modified approaches used to present summary findings from meta-analyses.* | **Page. 8-9** |
| Planned methods of analysis | 14 | Describe the methods of handling data and combining results of studies for each network meta-analysis. This should include, but not be limited to:   - *Handling of multi-arm trials;* - *Selection of variance structure;* - *Selection of prior distributions in Bayesian analyses; and* - *Assessment of model fit.* | **Page. 8-10** |
| **Assessment of Inconsistency** | **S2** | Describe the statistical methods used to evaluate the agreement of direct and indirect evidence in the treatment network(s) studied. Describe efforts taken to address its presence when found. | **Page. 9-10** |
| Risk of bias across studies | 15 | Specify any assessment of risk of bias that may affect the cumulative evidence (e.g., publication bias, selective reporting within studies). | **Page. 7-8** |
| Additional analyses | 16 | Describe methods of additional analyses if done, indicating which were pre-specified. This may include, but not be limited to, the following:   - Sensitivity or subgroup analyses; - Meta-regression analyses; - *Alternative formulations of the treatment network; and* - *Use of alternative prior distributions for Bayesian analyses (if applicable).* | **Page. 8 and Page.10** |

| **RESULTS†** | | |  |
| --- | --- | --- | --- |
| Study selection | 17 | Give numbers of studies screened, assessed for eligibility, and included in the review, with reasons for exclusions at each stage, ideally with a flow diagram. | **Page.10**  **And Figure.1** |
| **Presentation of network structure** | **S3** | Provide a network graph of the included studies to enable visualization of the geometry of the treatment network. | **Figure.2,3 and**  **Additional file 3: Fig. S3-4** |
| **Summary of network geometry** | **S4** | Provide a brief overview of characteristics of the treatment network. This may include commentary on the abundance of trials and randomized patients for the different interventions and pairwise comparisons in the network, gaps of evidence in the treatment network, and potential biases reflected by the network structure. | **Page. 11** |
| Study characteristics | 18 | For each study, present characteristics for which data were extracted (e.g., study size, PICOS, follow-up period) and provide the citations. | **Page.10-11**  **Table.1 and Additional file 2: Tab. S1** |
| Risk of bias within studies | 19 | Present data on risk of bias of each study and, if available, any outcome level assessment. | **eFigure.1, eFigure.2** |
| Results of individual studies | 20 | For all outcomes considered (benefits or harms), present, for each study: 1) simple summary data for each intervention group, and 2) effect estimates and confidence intervals.  *Modified approaches may be needed to deal with information from larger networks.* | **Page.11**  **Additional file 3: Fig. S1 , Additional file 3: Fig. S2 and Additional file 2: Tab. S13** |
| Synthesis of results | 21 | Present results of each meta-analysis done, including confidence/credible intervals. *In larger networks, authors may focus on comparisons versus a particular comparator (e.g. placebo or standard care), with full findings presented in an appendix. League tables and forest plots may be considered to summarize pairwise comparisons.* If additional summary measures were explored (such as treatment rankings), these should also be presented. | **Page.12-13,**  **Figure.2,3, Table.2,**  **Additional file 3: Fig. S3-4** |
| **Exploration for inconsistency** | **S5** | Describe results from investigations of inconsistency. This may include such information as measures of model fit to compare consistency and inconsistency models, *P* values from statistical tests, or summary of inconsistency estimates from different parts of the treatment network. | **Page.12-13 and Additional file 3: Fig. S13-16** |
| Risk of bias across studies | 22 | Present results of any assessment of risk of bias across studies for the evidence base being studied. | **Page.11, Additional file 3: Fig. S1 , Additional file 3: Fig. S2, Additional file 2: Tab. S13 and Additional file 2: Tab. S9-12**. |
| Results of additional analyses | 23 | Give results of additional analyses, if done (e.g., sensitivity or subgroup analyses, meta-regression analyses*, alternative network geometries studied, alternative choice of prior distributions for Bayesian analyses,* and so forth). | **Page.14,**  **Additional file 2: Tab. S7-8, and Additional file 3: Fig. S17-37** |
| **DISCUSSION** |  |  |  |
| Summary of evidence | 24 | Summarize the main findings, including the strength of evidence for each main outcome; consider their relevance to key groups (e.g., healthcare providers, users, and policy- makers). | **Page.14-19** |
| Limitations | 25 | Discuss limitations at study and outcome level (e.g., risk of bias), and at review level (e.g., incomplete retrieval of identified research, reporting bias). *Comment on the validity of the assumptions, such as transitivity and consistency. Comment* | **Page.18-19** |

| *on any concerns regarding network geometry (e.g., avoidance of certain comparisons).* | | |  |
| --- | --- | --- | --- |
| Conclusions | 26 | Provide a general interpretation of the results in the context of other evidence, and implications for future research. | **Page.19-20** |
| **FUNDING** |  |  | The work was supported by the National Natural Science Foundation of China (No. 81673139) |
| Funding | 27 | Describe sources of funding for the systematic review and other  support (e.g., supply of data); role of funders for the systematic review. This should also include information regarding whether funding has been received from manufacturers of treatments in the network and/or whether some of the authors are content experts with professional conflicts of interest that could affect use of treatments in the network. |  |

PICOS = population, intervention, comparators, outcomes, study design.

* Text in italics indicate wording specific to reporting of network meta-analyses that has been added to guidance from the PRISMA statement.

† Authors may wish to plan for use of appendices to present all relevant information in full detail for items in this section.

**Appendix. S2 Search strategies**

**PubMed**

1. "Exercise"[Mesh]
2. (((((((((((((((((((((((((Exercises[Title/Abstract]) OR (Physical Activity[Title/Abstract])) OR (Activities, Physical[Title/Abstract])) OR (Activity, Physical[Title/Abstract])) OR (Physical Activities[Title/Abstract])) OR (Exercise, Physical[Title/Abstract])) OR (Exercises, Physical[Title/Abstract])) OR (Physical Exercise[Title/Abstract])) OR (Physical Exercises[Title/Abstract])) OR (Acute Exercise[Title/Abstract])) OR (Acute Exercises[Title/Abstract])) OR (Exercise, Acute[Title/Abstract])) OR (Exercises, Acute[Title/Abstract])) OR (Exercise, Isometric[Title/Abstract])) OR (Exercises, Isometric[Title/Abstract])) OR (Isometric Exercises[Title/Abstract])) OR (Isometric Exercise[Title/Abstract])) OR (Exercise, Aerobic[Title/Abstract])) OR (Aerobic Exercise[Title/Abstract])) OR (Aerobic Exercises[Title/Abstract])) OR (Exercises, Aerobic[Title/Abstract])) OR (Exercise Training[Title/Abstract])) OR (Exercise Trainings[Title/Abstract])) OR (Training, Exercise[Title/Abstract])) OR (Trainings, Exercise[Title/Abstract]))
3. "Diet"[Mesh]
4. Diets[Title/Abstract]
5. "Healthy Lifestyle"[Mesh]
6. "Life Style"[Mesh]
7. "Adolescent"[MeSH Terms])
8. ("Adolescents"[Title/Abstract] OR "Adolescence Teens"[Title/Abstract] OR "Teen"[Title/Abstract] OR "Teenagers"[Title/Abstract] OR "Teenager"[Title/Abstract] OR "Youth"[Title/Abstract] OR "Youths"[Title/Abstract] OR "Adolescents, Female"[Title/Abstract] OR "Adolescent, Female"[Title/Abstract] OR "Female Adolescent"[Title/Abstract] OR "Female Adolescents"[Title/Abstract] OR "Adolescents, Male"[Title/Abstract] OR "Adolescent, Male"[Title/Abstract] OR "Male Adolescent"[Title/Abstract] OR "Male Adolescents"[Title/Abstract])) OR ("Child"[MeSH Terms])) OR ("Children"[Title/Abstract])
9. "Pediatric Obesity"[MeSH Terms])
10. ("Obesity, Pediatric"[Title/Abstract] OR "Childhood Onset Obesity"[Title/Abstract] OR "Obesity, Childhood Onset"[Title/Abstract] OR "Obesity in Childhood"[Title/Abstract] OR "Child Obesity"[Title/Abstract] OR "Obesity, Child"[Title/Abstract] OR "Childhood Obesity"[Title/Abstract] OR "Obesity, Childhood"[Title/Abstract] OR "Infant Overweight"[Title/Abstract] OR "Overweight, Infant"[Title/Abstract] OR "Infantile Obesity"[Title/Abstract] OR "Obesity, Infantile"[Title/Abstract] OR "Infant Obesity"[Title/Abstract] OR "Obesity, Infant"[Title/Abstract] OR "Childhood Overweight"[Title/Abstract] OR "Overweight, Childhood"[Title/Abstract] OR "Adolescent Overweight"[Title/Abstract] OR "Overweight, Adolescent"[Title/Abstract] OR "Adolescent Obesity"[Title/Abstract] OR "Obesity, Adolescent"[Title/Abstract] OR "Obesity in Adolescence"[Title/Abstract] OR "Male Adolescent"[Title/Abstract] OR "Adolescent, Male"[Title/Abstract] OR "Male Adolescent"[Title/Abstract])
11. (Life Styles) OR (Lifestyle) OR (Lifestyles) OR (Life Style Induced Illness) OR (Lifestyle Factors) OR (Factor, Lifestyle) OR (Lifestyle Factor)
12. (Lifestyle, Healthy) OR (Lifestyles, Healthy) OR (Healthy Life Styles) OR (Healthy Lifestyles) OR (Healthy Life Style) OR (Life Style, Healthy) OR (Life Styles, Healthy)
13. (randomized controlled trial[pt] OR controlled clinical trial[pt] OR randomized[tiab] OR placebo[tiab] OR clinical trials as topic[mesh:noexp] OR randomly[tiab] OR trial[ti]) NOT (animals [mh] NOT (humans [mh] AND animals[mh]))
14. ("Exercise"[Mesh]) OR (((((((((((((((((((((((((Exercises[Title/Abstract]) OR (Physical Activity[Title/Abstract])) OR (Activities, Physical[Title/Abstract])) OR (Activity, Physical[Title/Abstract])) OR (Physical Activities[Title/Abstract])) OR (Exercise, Physical[Title/Abstract])) OR (Exercises, Physical[Title/Abstract])) OR (Physical Exercise[Title/Abstract])) OR (Physical Exercises[Title/Abstract])) OR (Acute Exercise[Title/Abstract])) OR (Acute Exercises[Title/Abstract])) OR (Exercise, Acute[Title/Abstract])) OR (Exercises, Acute[Title/Abstract])) OR (Exercise, Isometric[Title/Abstract])) OR (Exercises, Isometric[Title/Abstract])) OR (Isometric Exercises[Title/Abstract])) OR (Isometric Exercise[Title/Abstract])) OR (Exercise, Aerobic[Title/Abstract])) OR (Aerobic Exercise[Title/Abstract])) OR (Aerobic Exercises[Title/Abstract])) OR (Exercises, Aerobic[Title/Abstract])) OR (Exercise Training[Title/Abstract])) OR (Exercise Trainings[Title/Abstract])) OR (Training, Exercise[Title/Abstract])) OR (Trainings, Exercise[Title/Abstract]))
15. ("Diet"[Mesh]) OR (Diets[Title/Abstract])
16. ((("Healthy Lifestyle"[Mesh]) OR ((Life Styles) OR (Lifestyle) OR (Lifestyles) OR (Life Style Induced Illness) OR (Lifestyle Factors) OR (Factor, Lifestyle) OR (Lifestyle Factor))) OR ("Life Style"[Mesh])) OR ((Lifestyle, Healthy) OR (Lifestyles, Healthy) OR (Healthy Life Styles) OR (Healthy Lifestyles) OR (Healthy Life Style) OR (Life Style, Healthy) OR (Life Styles, Healthy))
17. ("Exercise"[Mesh]) OR (((((((((((((((((((((((((Exercises[Title/Abstract]) OR (Physical Activity[Title/Abstract])) OR (Activities, Physical[Title/Abstract])) OR (Activity, Physical[Title/Abstract])) OR (Physical Activities[Title/Abstract])) OR (Exercise, Physical[Title/Abstract])) OR (Exercises, Physical[Title/Abstract])) OR (Physical Exercise[Title/Abstract])) OR (Physical Exercises[Title/Abstract])) OR (Acute Exercise[Title/Abstract])) OR (Acute Exercises[Title/Abstract])) OR (Exercise, Acute[Title/Abstract])) OR (Exercises, Acute[Title/Abstract])) OR (Exercise, Isometric[Title/Abstract])) OR (Exercises, Isometric[Title/Abstract])) OR (Isometric Exercises[Title/Abstract])) OR (Isometric Exercise[Title/Abstract])) OR (Exercise, Aerobic[Title/Abstract])) OR (Aerobic Exercise[Title/Abstract])) OR (Aerobic Exercises[Title/Abstract])) OR (Exercises, Aerobic[Title/Abstract])) OR (Exercise Training[Title/Abstract])) OR (Exercise Trainings[Title/Abstract])) OR (Training, Exercise[Title/Abstract])) OR (Trainings, Exercise[Title/Abstract])) OR ("Diet"[Mesh]) OR (Diets[Title/Abstract]) OR ((("Healthy Lifestyle"[Mesh]) OR ((Life Styles) OR (Lifestyle) OR (Lifestyles) OR (Life Style Induced Illness) OR (Lifestyle Factors) OR (Factor, Lifestyle) OR (Lifestyle Factor))) OR ("Life Style"[Mesh])) OR ((Lifestyle, Healthy) OR (Lifestyles, Healthy) OR (Healthy Life Styles) OR (Healthy Lifestyles) OR (Healthy Life Style) OR (Life Style, Healthy) OR (Life Styles, Healthy))
18. "Pediatric Obesity"[MeSH Terms]) OR ("Adolescents"[Title/Abstract] OR "Adolescence Teens"[Title/Abstract] OR "Teen"[Title/Abstract] OR "Teenagers"[Title/Abstract] OR "Teenager"[Title/Abstract] OR "Youth"[Title/Abstract] OR "Youths"[Title/Abstract] OR "Adolescents, Female"[Title/Abstract] OR "Adolescent, Female"[Title/Abstract] OR "Female Adolescent"[Title/Abstract] OR "Female Adolescents"[Title/Abstract] OR "Adolescents, Male"[Title/Abstract] OR "Adolescent, Male"[Title/Abstract] OR "Male Adolescent"[Title/Abstract] OR "Male Adolescents"[Title/Abstract])) OR ("Child"[MeSH Terms])) OR ("Children"[Title/Abstract])
19. "Pediatric Obesity"[MeSH Terms]) OR ("Obesity, Pediatric"[Title/Abstract] OR "Childhood Onset Obesity"[Title/Abstract] OR "Obesity, Childhood Onset"[Title/Abstract] OR "Obesity in Childhood"[Title/Abstract] OR "Child Obesity"[Title/Abstract] OR "Obesity, Child"[Title/Abstract] OR "Childhood Obesity"[Title/Abstract] OR "Obesity, Childhood"[Title/Abstract] OR "Infant Overweight"[Title/Abstract] OR "Overweight, Infant"[Title/Abstract] OR "Infantile Obesity"[Title/Abstract] OR "Obesity, Infantile"[Title/Abstract] OR "Infant Obesity"[Title/Abstract] OR "Obesity, Infant"[Title/Abstract] OR "Childhood Overweight"[Title/Abstract] OR "Overweight, Childhood"[Title/Abstract] OR "Adolescent Overweight"[Title/Abstract] OR "Overweight, Adolescent"[Title/Abstract] OR "Adolescent Obesity"[Title/Abstract] OR "Obesity, Adolescent"[Title/Abstract] OR "Obesity in Adolescence"[Title/Abstract] OR "Male Adolescent"[Title/Abstract] OR "Adolescent, Male"[Title/Abstract] OR "Male Adolescent"[Title/Abstract])
20. ("Exercise"[Mesh]) OR (((((((((((((((((((((((((Exercises[Title/Abstract]) OR (Physical Activity[Title/Abstract])) OR (Activities, Physical[Title/Abstract])) OR (Activity, Physical[Title/Abstract])) OR (Physical Activities[Title/Abstract])) OR (Exercise, Physical[Title/Abstract])) OR (Exercises, Physical[Title/Abstract])) OR (Physical Exercise[Title/Abstract])) OR (Physical Exercises[Title/Abstract])) OR (Acute Exercise[Title/Abstract])) OR (Acute Exercises[Title/Abstract])) OR (Exercise, Acute[Title/Abstract])) OR (Exercises, Acute[Title/Abstract])) OR (Exercise, Isometric[Title/Abstract])) OR (Exercises, Isometric[Title/Abstract])) OR (Isometric Exercises[Title/Abstract])) OR (Isometric Exercise[Title/Abstract])) OR (Exercise, Aerobic[Title/Abstract])) OR (Aerobic Exercise[Title/Abstract])) OR (Aerobic Exercises[Title/Abstract])) OR (Exercises, Aerobic[Title/Abstract])) OR (Exercise Training[Title/Abstract])) OR (Exercise Trainings[Title/Abstract])) OR (Training, Exercise[Title/Abstract])) OR (Trainings, Exercise[Title/Abstract])) OR ("Diet"[Mesh]) OR (Diets[Title/Abstract]) OR ((("Healthy Lifestyle"[Mesh]) OR ((Life Styles) OR (Lifestyle) OR (Lifestyles) OR (Life Style Induced Illness) OR (Lifestyle Factors) OR (Factor, Lifestyle) OR (Lifestyle Factor))) OR ("Life Style"[Mesh])) OR ((Lifestyle, Healthy) OR (Lifestyles, Healthy) OR (Healthy Life Styles) OR (Healthy Lifestyles) OR (Healthy Life Style) OR (Life Style, Healthy) OR (Life Styles, Healthy)) AND "Pediatric Obesity"[MeSH Terms]) OR ("Adolescents"[Title/Abstract] OR "Adolescence Teens"[Title/Abstract] OR "Teen"[Title/Abstract] OR "Teenagers"[Title/Abstract] OR "Teenager"[Title/Abstract] OR "Youth"[Title/Abstract] OR "Youths"[Title/Abstract] OR "Adolescents, Female"[Title/Abstract] OR "Adolescent, Female"[Title/Abstract] OR "Female Adolescent"[Title/Abstract] OR "Female Adolescents"[Title/Abstract] OR "Adolescents, Male"[Title/Abstract] OR "Adolescent, Male"[Title/Abstract] OR "Male Adolescent"[Title/Abstract] OR "Male Adolescents"[Title/Abstract])) OR ("Child"[MeSH Terms])) OR ("Children"[Title/Abstract]) AND "Pediatric Obesity"[MeSH Terms]) OR ("Obesity, Pediatric"[Title/Abstract] OR "Childhood Onset Obesity"[Title/Abstract] OR "Obesity, Childhood Onset"[Title/Abstract] OR "Obesity in Childhood"[Title/Abstract] OR "Child Obesity"[Title/Abstract] OR "Obesity, Child"[Title/Abstract] OR "Childhood Obesity"[Title/Abstract] OR "Obesity, Childhood"[Title/Abstract] OR "Infant Overweight"[Title/Abstract] OR "Overweight, Infant"[Title/Abstract] OR "Infantile Obesity"[Title/Abstract] OR "Obesity, Infantile"[Title/Abstract] OR "Infant Obesity"[Title/Abstract] OR "Obesity, Infant"[Title/Abstract] OR "Childhood Overweight"[Title/Abstract] OR "Overweight, Childhood"[Title/Abstract] OR "Adolescent Overweight"[Title/Abstract] OR "Overweight, Adolescent"[Title/Abstract] OR "Adolescent Obesity"[Title/Abstract] OR "Obesity, Adolescent"[Title/Abstract] OR "Obesity in Adolescence"[Title/Abstract] OR "Male Adolescent"[Title/Abstract] OR "Adolescent, Male"[Title/Abstract] OR "Male Adolescent"[Title/Abstract]) AND (randomized controlled trial[pt] OR controlled clinical trial[pt] OR randomized[tiab] OR placebo[tiab] OR clinical trials as topic[mesh:noexp] OR randomly[tiab] OR trial[ti]) NOT (animals [mh] NOT (humans [mh] AND animals[mh]))

**Embase**

1. 'exercise'/exp OR 'exercise'
2. 'physical activity':ab,ti OR 'activities, physical':ab,ti OR 'activity, physical':ab,ti OR 'exercises':ab,ti OR 'physical activities':ab,ti OR 'exercise, physical':ab,ti OR 'exercises, physical':ab,ti OR 'physical exercise':ab,ti OR 'acute exercise':ab,ti OR 'acute exercises':ab,ti OR 'exercise, acute':ab,ti OR 'exercises, acute':ab,ti OR 'exercise, isometric':ab,ti OR 'exercises, isometric':ab,ti OR 'isometric exercises':ab,ti OR 'isometric exercise':ab,ti OR 'exercise, aerobic':ab,ti OR 'aerobic exercise':ab,ti OR 'aerobic exercises':ab,ti OR 'exercises, aerobic':ab,ti OR 'exercise training':ab,ti OR 'exercise trainings':ab,ti OR 'training, exercise':ab,ti OR 'heart attack':ab,ti OR 'trainings, exercise':ab,ti
3. 'resistance training'/exp
4. 'training, resistance':ab,ti OR 'strength training':ab,ti OR 'training, strength':ab,ti OR 'weight-lifting strengthening program':ab,ti OR 'strengthening program, weight-lifting':ab,ti OR 'strengthening programs, weight-lifting':ab,ti OR 'weight lifting strengthening program':ab,ti OR 'weight-lifting strengthening programs':ab,ti OR 'physical activities':ab,ti OR 'exercise program, weight-lifting':ab,ti OR 'weight-lifting exercise program':ab,ti OR 'exercise programs, weight-lifting':ab,ti OR 'weight lifting exercise program':ab,ti OR 'weight-lifting exercise programs':ab,ti OR 'strengthening programs, weight-bearing':ab,ti OR 'strengthening program, weight-bearing':ab,ti OR 'weight-bearing strengthening program':ab,ti OR 'weight bearing strengthening program':ab,ti OR 'weight-bearing strengthening programs':ab,ti OR 'weight-bearing exercise program':ab,ti OR 'exercise program, weight-bearing':ab,ti OR 'exercise programs, weight-bearing':ab,ti OR 'weight bearing exercise program':ab,ti OR 'weight-bearing exercise programs':ab,ti
5. 'endurance'/exp
6. 'stamina, physical':ab,ti OR 'physical stamina':ab,ti OR 'endurance, physical':ab,ti
7. 'stretching exercise'/exp
8. 'exercise, muscle stretching':ab,ti OR 'exercises, muscle stretching':ab,ti OR 'muscle stretching exercise':ab,ti OR 'static stretching':ab,ti OR 'stretching, static':ab,ti OR 'stretching, relaxed':ab,ti OR 'stretching, passive':ab,ti OR 'relaxed stretching':ab,ti OR 'static-passive stretching':ab,ti OR 'static passive stretching':ab,ti OR 'stretching, static-passive':ab,ti OR 'isometric stretching':ab,ti OR 'stretching, isometric':ab,ti OR 'active stretching':ab,ti OR 'stretching, active':ab,ti OR 'static-active stretching':ab,ti OR 'static active stretching':ab,ti OR 'stretching, static-active':ab,ti OR 'ballistic stretching':ab,ti OR 'stretching, ballistic':ab,ti OR 'dynamic stretching':ab,ti OR 'stretching, dynamic':ab,ti OR 'proprioceptive neuromuscular facilitation (pnf) stretching':ab,ti
9. 'diet'/exp
10. 'diet':ab,ti
11. 'lifestyle'/exp
12. 'healthy lifestyle':ab,ti OR 'life styles':ab,ti OR 'lifestyles':ab,ti OR 'life style induced illness':ab,ti OR 'lifestyle factors':ab,ti OR 'factor, lifestyle':ab,ti OR 'life style':ab,ti OR 'lifestyle':ab,ti OR 'lifestyles, healthy':ab,ti OR 'healthy life styles':ab,ti OR 'healthy lifestyles':ab,ti OR 'healthy life style':ab,ti OR 'life style, healthy':ab,ti OR 'lifestyle factor':ab,ti OR 'life styles, healthy':ab,ti
13. 'child'/exp
14. 'children':ab,ti
15. 'childhood obesity'/exp
16. 'obesity, pediatric':ab,ti OR 'childhood onset obesity':ab,ti OR 'obesity, childhood onsets':ab,ti OR 'obesity in childhood':ab,ti OR 'child obesity':ab,ti OR 'obesity, child':ab,ti OR 'childhood obesity':ab,ti OR 'obesity, childhood':ab,ti OR 'infant overweight':ab,ti OR 'overweight, infant':ab,ti OR 'infantile obesity':ab,ti OR 'obesity in adolescence':ab,ti OR 'obesity, infantile':ab,ti OR 'infant obesity':ab,ti OR 'obesity, adolescent':ab,ti OR 'obesity, infant':ab,ti OR 'childhood overweight':ab,ti OR 'adolescent obesity':ab,ti OR 'overweight, childhood':ab,ti OR 'adolescent overweight':ab,ti OR 'overweight, adolescent':ab,ti OR 'teen':ab,ti
17. 'crossover procedure':de OR 'double-blind procedure':de OR 'randomized controlled trial':de OR 'single-blind procedure':de OR random*:de,ab,ti OR factorial*:de,ab,ti OR crossover*:de,ab,ti OR ((cross NEXT/1 over*):de,ab,ti) OR placebo*:de,ab,ti OR ((doubl* NEAR/1 blind*):de,ab,ti) OR ((singl* NEAR/1 blind*):de,ab,ti) OR assign*:de,ab,ti OR allocat*:de,ab,ti OR volunteer*:de,ab,ti
18. #1 OR #2 OR #3 OR #4 OR #5 OR #6 OR #7 OR #8
19. #9 OR #10
20. #11 OR #12
21. #13 OR #14
22. #15 OR #16
23. #18 OR #19 OR #20
24. #17 AND #21 AND #22 AND #23

**Cochrane Library**

1. MeSH descriptor: [Exercise] explode all trees
2. MeSH descriptor: [Resistance Training] explode all trees
3. MeSH descriptor: [Physical Endurance] explode all trees
4. MeSH descriptor: [Muscle Stretching Exercises] explode all trees
5. (Exercises):ti,ab,kw OR (Physical Activity):ti,ab,kw OR (Activities, Physical):ti,ab,kw OR (Activity, Physical):ti,ab,kw OR (Physical Activities):ti,ab,kw OR (Exercise, Physical):ti,ab,kw OR (Exercises, Physical):ti,ab,kw OR (Acute Exercises):ti,ab,kw OR (Exercise, Acute):ti,ab,kw OR (Exercises, Acute):ti,ab,kw OR (Exercise, Isometric):ti,ab,kw OR (Exercises, Isometric):ti,ab,kw OR (Isometric Exercises):ti,ab,kw OR (Isometric Exercise):ti,ab,kw OR (Exercise, Aerobic):ti,ab,kw OR (Aerobic Exercise):ti,ab,kw OR (Aerobic Exercises):ti,ab,kw OR (Exercises, Aerobic):ti,ab,kw OR (Exercise Training):ti,ab,kw OR (Exercise Trainings):ti,ab,kw OR (Training, Exercise):ti,ab,kw OR (Trainings, Exercise):ti,ab,kw
6. (Training, Resistance):ti,ab,kw OR (Strength Training):ti,ab,kw OR (Training, Strength):ti,ab,kw OR (Weight-Lifting Strengthening Program):ti,ab,kw OR (Strengthening Program, Weight-Lifting):ti,ab,kw OR (Strengthening Programs, Weight-Lifting):ti,ab,kw OR (Weight Lifting Strengthening Program):ti,ab,kw OR (Weight-Lifting Strengthening Programs):ti,ab,kw OR (Weight-Lifting Exercise Program):ti,ab,kw OR (Exercise Program, Weight-Lifting):ti,ab,kw OR (Exercise Programs, Weight-Lifting):ti,ab,kw OR (Weight Lifting Exercise Program):ti,ab,kw OR (Weight-Lifting Exercise Programs):ti,ab,kw OR (Weight-Bearing Strengthening Program):ti,ab,kw OR (Strengthening Program, Weight-Bearing):ti,ab,kw OR (Strengthening Programs, Weight-Bearing):ti,ab,kw OR (Weight Bearing Strengthening Program):ti,ab,kw OR (Weight-Bearing Strengthening Programs):ti,ab,kw OR (Weight-Bearing Exercise Program):ti,ab,kw OR (Exercise Program, Weight-Bearing):ti,ab,kw OR (Exercise Programs, Weight-Bearing):ti,ab,kw OR (Weight Bearing Exercise Program):ti,ab,kw OR (Weight-Bearing Exercise Programs):ti,ab,kw
7. (Stamina, Physical):ti,ab,kw OR (Physical Stamina):ti,ab,kw OR (Endurance, Physical):ti,ab,kw
8. (Exercise, Muscle Stretching):ti,ab,kw OR (Exercises, Muscle Stretching):ti,ab,kw OR (Muscle Stretching Exercise):ti,ab,kw OR (Static Stretching):ti,ab,kw OR (Stretching, Static):ti,ab,kw OR (Passive Stretching):ti,ab,kw OR (Stretching, Passive):ti,ab,kw OR (Relaxed Stretching):ti,ab,kw OR (Stretching, Relaxed):ti,ab,kw OR (Static-Passive Stretching):ti,ab,kw OR (Endurance, Physical):ti,ab,kw OR (Static Passive Stretching):ti,ab,kw OR (Stretching, Static-Passive):ti,ab,kw OR (Isometric Stretching):ti,ab,kw OR (Stretching, Isometric):ti,ab,kw OR (Active Stretching):ti,ab,kw OR (Stretching, Active):ti,ab,kw OR (Static-Active Stretching):ti,ab,kw OR (Static Active Stretching):ti,ab,kw OR (Stretching, Static-Active):ti,ab,kw OR (Ballistic Stretching):ti,ab,kw OR (Stretching, Ballistic):ti,ab,kw OR (Dynamic Stretching):ti,ab,kw OR (Stretching, Dynamic):ti,ab,kw OR (Proprioceptive Neuromuscular Facilitation (PNF) Stretching):ti,ab,kw
9. (diets):ti,ab,kw
10. (Healthy Lifestyle):ti,ab,kw OR (Life Styles):ti,ab,kw OR (Lifestyle):ti,ab,kw OR (Lifestyles):ti,ab,kw OR (Life Style Induced Illness):ti,ab,kw OR (Lifestyle Factors):ti,ab,kw OR (Factor, Lifestyle):ti,ab,kw OR (Lifestyle Factor):ti,ab,kw OR (Lifestyle, Healthy):ti,ab,kw OR (Lifestyles, Healthy):ti,ab,kw OR (Healthy Life Styles):ti,ab,kw OR (Healthy Lifestyles):ti,ab,kw OR (Healthy Life Style):ti,ab,kw OR (Life Style, Healthy):ti,ab,kw OR (Life Styles, Healthy):ti,ab,kw
11. MeSH descriptor: [Adolescent] explode all trees
12. MeSH descriptor: [Child] explode all trees
13. (Adolescents):ti,ab,kw OR (Adolescence):ti,ab,kw OR (Teens):ti,ab,kw OR (Teen):ti,ab,kw OR (Teenagers):ti,ab,kw OR (Teenager):ti,ab,kw OR (Youth):ti,ab,kw OR (Youths):ti,ab,kw OR (Adolescents, Female):ti,ab,kw OR (Adolescent, Female):ti,ab,kw OR (Female Adolescent):ti,ab,kw OR (Female Adolescents):ti,ab,kw OR (Adolescents, Male):ti,ab,kw OR (Adolescent, Male):ti,ab,kw OR (Male Adolescent):ti,ab,kw OR (Male Adolescents):ti,ab,kw
14. (Children):ti,ab,kw
15. MeSH descriptor: [Pediatric Obesity] explode all trees
16. (Obesity, Pediatric):ti,ab,kw OR (Childhood Onset Obesity):ti,ab,kw OR (Obesity, Childhood Onset):ti,ab,kw OR (Obesity in Childhood):ti,ab,kw OR (Child Obesity):ti,ab,kw OR (Obesity, Child):ti,ab,kw OR (Childhood Obesity):ti,ab,kw OR (Obesity, Childhood):ti,ab,kw OR (Infant Overweight):ti,ab,kw OR (Overweight, Infant):ti,ab,kw OR (Infantile Obesity):ti,ab,kw OR (Obesity, Infantile):ti,ab,kw OR (Infant Obesity):ti,ab,kw OR (Obesity, Infant):ti,ab,kw OR (Childhood Overweight):ti,ab,kw OR (Overweight, Childhood):ti,ab,kw OR (Adolescent Overweight):ti,ab,kw OR (Overweight, Adolescent):ti,ab,kw OR (Adolescent Obesity):ti,ab,kw OR (Obesity, Adolescent):ti,ab,kw OR (Obesity in Adolescence):ti,ab,kw
17. #1 OR #2 OR #3 OR #4 OR #5 OR #6 OR #7 OR #8 OR #9 OR #10
18. #11 OR #12 OR #13 OR #14
19. #15 OR #16
20. #17 AND #18 AND #19

**Web of Science**

1. TS=(Exercise OR Exercises OR Physical Activity OR Activities, Physical OR Activity, Physical OR Physical Activities OR Exercise, Physical OR Exercises, Physical OR Physical Exercise OR Physical Exercises OR Acute Exercise, Physical OR Acute Exercises OR Exercise, Acute OR Exercises, Acute OR Exercise, Isometric OR Exercises, Isometric OR Isometric Exercises OR Isometric Exercise OR Exercise, Aerobic OR Aerobic Exercise OR Aerobic Exercises OR Exercises, Aerobic OR Exercise Training OR Exercise Trainings OR Training, Exercise OR Trainings, Exercise)
2. TS=(Resistance Training OR Acute Exercise, Physical OR Training, Resistance OR Strength Training OR Training, Strength OR Weight-Lifting Strengthening Program OR Strengthening Program, Weight-Lifting OR Strengthening Programs, Weight-Lifting OR Weight Lifting Strengthening Program OR Weight-Lifting Strengthening Programs OR Weight-Lifting Exercise Program OR Exercise Program, Weight-Lifting OR Exercise Programs, Weight-Lifting OR Weight Lifting Exercise Program OR Weight-Lifting Exercise Programs OR Weight-Bearing Strengthening Program OR Strengthening Program, Weight-Bearing OR Strengthening Programs, Weight-Bearing OR Weight Bearing Strengthening Program OR Weight-Bearing Strengthening Programs OR Weight-Bearing Exercise Program OR Exercise Program, Weight-Bearing OR Exercise Programs, Weight-Bearing OR Weight Bearing Exercise Program OR Weight-Bearing Exercise Programs)
3. TS=(Physical Endurance OR Stamina, Physical OR Physical Stamina OR Endurance, Physical) TS=(Muscle Stretching Exercises OR Exercise, Muscle Stretching OR Exercises, Muscle Stretching OR Muscle Stretching Exercise OR Static Stretching OR Stretching, Static OR Passive Stretching OR Stretching, Passive OR Relaxed Stretching OR Stretching, Relaxed OR Static-Passive Stretching OR Static Passive Stretching OR Stretching, Static-Passive OR Isometric Stretching OR Stretching, Isometric OR Active Stretching OR Stretching, Active OR Static-Active Stretching OR Static Active Stretching OR Stretching, Static-Active OR Ballistic Stretching OR Stretching, Ballistic OR Dynamic Stretching OR Stretching, Dynamic OR Proprioceptive Neuromuscular Facilitation (PNF) Stretching)
4. TS=(Diet OR Diets)
5. TS=(Healthy Lifestyle OR Life Styles OR Lifestyle OR Lifestyles OR Life Style Induced Illness OR Lifestyle Factors OR Factor, Lifestyle OR Lifestyle Factor OR Lifestyle, Healthy OR Lifestyles, Healthy OR Healthy Life Styles OR Healthy Lifestyles OR Healthy Life Style OR Life Style, Healthy OR Life Styles, Healthy)
6. TS=(Child OR Children)
7. TS=(Pediatric Obesity OR Obesity, Pediatric OR Childhood Onset Obesity OR Obesity, Childhood Onset OR Obesity in Childhood OR Child Obesity OR Obesity, Child OR Childhood Obesity OR Obesity, Childhood OR Infant Overweight OR Obesity, Infantile OR Infant Obesity OR Overweight, Infant OR Infantile Obesity OR Obesity, Infant OR Childhood Overweight OR Overweight, Childhood OR Adolescent Overweight OR Overweight, Adolescent OR Adolescent Obesity OR Obesity, Adolescent OR Obesity in Adolescence)
8. TS= clinical trial* OR TS=research design OR TS=comparative stud* OR TS=evaluation stud* OR TS=controlled trial* OR TS=follow-up stud* OR TS=prospective stud* OR TS=random* OR TS=placebo* OR TS=(single blind*) OR TS=(double blind*)
9. #6 OR #5 OR #4 OR #3 OR #2 OR #1

#10 AND #9 AND #8 AND #

**Appendix. S3 Inclusion and Exclusion criteria**

| Items | Inclusion criteria | Exclusion criteria |
| --- | --- | --- |
| P (population) | Studies recruited participants that were children or adolescents aged from 6 to 18 years old with their standardized diagnostic measures of obesity. | If most enrolled individuals were substance withdrawal. |
| I (Interventions) | Interventions were any type of PA(e.g., aerobic exercise, resistance training, endurance exercise), DI(e.g, Very low-carbohydrate diet, Very low-energy diet, Low-fat diet), MLI, or any the abovementioned interventions in combinatorial or multicomponent, whether they were delivered by MH technology or FTF approach. | 1) Any intervention in combinatorial or multicomponent, the definition of intervention in study is vague; 2) Any intervention study that combines or compares with a pharmacological. |
| C (Comparator) | Comparators were various interventions themselves or NCG alone such as wait-list control group, treatment as usual. | NCG in any combination with any existing interventions. |
| O (Outcome) | Children with obesity or any prevalent subtypes, which was assessed by measurable instruments or the quantifiable indicators with quadratic transformation, such as BMI, BMI Z-score and WC. | Outcomes were presented with biological indicators or other measures that cannot produce the intersecting endpoint for our analyses. |
| S (Study design) | Any type of RCTs whether they have designed into parallel or cross-over setting. Without any race, region, publication year and language restriction on the above items. | Non-randomized trials, such as protocols, population-based observational studies, or studies did not contain the indispensable data. |
| T (Time stamp and database) | PubMed, Embase, the Cochrane Central Register of Controlled Trials, Web of Science, PsycINFO, CINAHL the Chinese Biomedical Literature Database and Clinical trials were used to perform an exhaustive search to identify relevant studies from their inception to December 1, 2020, in accordance with the Cochrane Handbook for Systematic Reviews of Interventions. | |

BMI, Body mass index; DI, Dietary intervention; FTF, Face-to-face; MH, Mobile health-based; MLI, Muti-lifestyle intervention; NCG, Named control group; PA, Physical activity; WC, Waist circumference.

**Appendix. S4 Main analysis OpenBUGS code**

**model{ # *** PROGRAM STARTS**

**for(i in 1:ns){ # LOOP THROUGH STUDIES**

**w[i,1] <- 0 # adjustment for multi-arm trials is zero for control arm**

**delta[i,1] <- 0 # treatment effect is zero for control arm**

**mu[i] ~ dnorm(0,.0001) # vague priors for all trial baselines**

**for (k in 1:na[i]) { # LOOP THROUGH ARMS**

**var[i,k] <- pow(se[i,k],2) # calculate variances**

**prec[i,k] <- 1/var[i,k] # set precisions**

**y[i,k] ~ dnorm(theta[i,k],prec[i,k]) # normal likelihood**

**theta[i,k] <- mu[i] + delta[i,k] # model for linear predictor**

**dev[i,k] <- (y[i,k]-theta[i,k])*(y[i,k]-theta[i,k])*prec[i,k] #Deviance contribution**

**}**

**resdev[i] <- sum(dev[i,1:na[i]]) # summed residual deviance contribution for this trial**

**for (k in 2:na[i]) { # LOOP THROUGH ARMS**

**delta[i,k] ~ dnorm(md[i,k],taud[i,k]) # trial-specific MD distributions**

**md[i,k] <- d[t[i,k]] - d[t[i,1]] + sw[i,k] # mean of treat effects distributions (with multi-arm trial correction)**

**taud[i,k] <- tau *2*(k-1)/k # precision of treat effects distributions (with multi-arm trial correction)**

**w[i,k] <- (delta[i,k] - d[t[i,k]] + d[t[i,1]]) # adjustment for multi-arm RCTs**

**sw[i,k] <- sum(w[i,1:k-1])/(k-1) # cumulative adjustment for multi-arm trials**

**}**

**}**

**totresdev <- sum(resdev[]) #Total Residual Deviance**

**d[1]<-0 # treatment effect is zero for reference treatment**

**for (k in 2:nt){ d[k] ~ dnorm(0,.0001) } # vague priors for treatment effects**

**sd ~ dunif(0,5) # vague prior for between-trial SD.**

**tau <- pow(sd,-2) # between-trial precision = (1/between-trial variance)**

**# Ranking and probabilities for treatment**

**for(k in 1:nt) {**

**order[k]<- rank(d[],k)**

**most.effective[k]<-equals(order[k],1)**

**for(j in 1:nt) {**

**effectiveness[k,j]<- equals(order[k],j)**

**cumeffectiveness[k,j]<- sum(effectiveness[k,1:j])**

**}**

**}**

**#SUCRA**

**for(k in 1:nt) {**

**SUCRA[k]<- sum(cumeffectiveness[k,1:(nt-1)]) /(nt-1)**

**}**

**# all MDs for each treatment level comparison**

**for (c in 1:(nt-1)) {**

**for (k in (c+1):nt) {**

**MD[c,k] <- (d[k]-d[c]) } }**

**}**

1. **Subgroup analysis**

**model{ # *** PROGRAM STARTS**

**for(i in 1:ns){ # LOOP THROUGH STUDIES**

**w[i,1] <- 0 # adjustment for multi-arm trials is zero for control arm**

**delta[i,1] <- 0 # treatment effect is zero for control arm**

**mu[i] ~ dnorm(0,.0001) # vague priors for all trial baselines**

**for (k in 1:na[i]) { # LOOP THROUGH ARMS**

**var[i,k] <- pow(se[i,k],2) # calculate variances**

**prec[i,k] <- 1/var[i,k] # set precisions**

**y[i,k] ~ dnorm(theta[i,k],prec[i,k]) # normal likelihood**

**theta[i,k] <- mu[i] + delta[i,k]+ (beta[t[i,k]]-beta[t[i,1]]) * x[i]# model for linear predictor**

**dev[i,k] <- (y[i,k]-theta[i,k])*(y[i,k]-theta[i,k])*prec[i,k] #Deviance contribution**

**}**

**resdev[i] <- sum(dev[i,1:na[i]]) # summed residual deviance contribution for this trial**

**for (k in 2:na[i]) { # LOOP THROUGH ARMS**

**delta[i,k] ~ dnorm(md[i,k],taud[i,k]) # trial-specific LOR distributions**

**md[i,k] <- d[t[i,k]] - d[t[i,1]] + sw[i,k] # mean of treat effects distributions (with multi-arm trial correction)**

**taud[i,k] <- tau *2*(k-1)/k # precision of treat effects distributions (with multi-arm trial correction)**

**w[i,k] <- (delta[i,k] - d[t[i,k]] + d[t[i,1]]) # adjustment for multi-arm RCTs**

**sw[i,k] <- sum(w[i,1:k-1])/(k-1) # cumulative adjustment for multi-arm trials**

**}**

**}**

**totresdev <- sum(resdev[]) #Total Residual Deviance**

**d[1]<-0 # treatment effect is zero for reference treatment**

**beta[1] <- 0 # covariate effect is zero for reference treatment**

**for (k in 2:nt){**

**d[k] ~ dnorm(0,.0001) # vague priors for treatment effects**

**beta[k] <- B # common covariate effect**

**}**

**B ~ dnorm(0,.0001) # vague prior for covariate effect**

**sd ~ dunif(0,5) # vague prior for between-trial SD.**

**tau <- pow(sd,-2) # between-trial precision = (1/between-trial variance)**

**for (k in 1:nt){**

**for (j in 1:nz) { dz[j,k] <- d[k] + (beta[k]-beta[1])*z[j] } # treatment effect when covariate = z[j]**

**}**

**# MD for all possible pair-wise comparisons**

**for (c in 1:(nt-1)) {**

**for (k in (c+1):nt) {**

**# when covariate is zero**

**MD[c,k] <- (d[k]-d[c])**

**# at covariate=z[j]**

**for (j in 1:nz) {**

**MDz[j,c,k] <- (dz[j,k]-dz[j,c])**

**}**

**}**

**}**

**# Ranking and probabilities for treatment when covariate=0**

**for(k in 1:nt) {**

**order[k]<- rank(d[],k)**

**most.effective[k]<-equals(order[k],1)**

**for(j in 1:nt) {**

**effectiveness[k,j]<- equals(order[k],j)**

**cumeffectiveness[k,j]<- sum(effectiveness[k,1:j])**

**}**

**}**

**#SUCRA**

**for(k in 1:nt) {**

**SUCRA[k]<- sum(cumeffectiveness[k,1:(nt-1)]) /(nt-1)**

**}**

**# Ranking and probabilities for treatment when covariate=1**

**for(k in 1:nt) {**

**orderz[k]<- rank(dz[,],k)**

**most.effectivez[k]<-equals(orderz[k],1)**

**for(j in 1:nt) {**

**effectivenessz[k,j]<- equals(orderz[k],j)**

**cumeffectivenessz[k,j]<- sum(effectivenessz[k,1:j])**

**}**

**}**

**#SUCRA when covariate=1**

**Appendix. S5 The references for 118 included studies**

1. Andrade S, Lachat C, Ochoa-Aviles A, Verstraeten R, Huybregts L, Roberfroid D, Andrade D, Camp J, Rojas R, Donoso S *et al*: **A school-based intervention improves physical fitness in Ecuadorian adolescents: a cluster-randomized controlled trial**. *The international journal of behavioral nutrition physical activity* 2014, **11**:153.

2. Arauz Boudreau A, Kurowski D, Gonzalez W, Dimond M, Oreskovic N: **Latino families, primary care, and childhood obesity: a randomized controlled trial**. *Am J Prev Med* 2013, **44**:S247-257.

3. Balagopal P, Bayne E, Sager B, Russell L, Patton N, George D: **Effect of lifestyle changes on whole-body protein turnover in obese adolescents**. *International journal of obesity related metabolic disorders : journal of the International Association for the Study of Obesity* 2003, **27**(10):1250-1257.

4. Black M, Hager E, Le K, Anliker J, Arteaga S, Diclemente C, Gittelsohn J, Magder L, Papas M, Snitker S *et al*: **Challenge! Health promotion/obesity prevention mentorship model among urban, black adolescents**. *Pediatrics* 2010, **126**(2):280-288.

5. Boodai S, McColl J, Reilly J: **National Adolescent Treatment Trial for Obesity in Kuwait (NATTO): project design and results of a randomised controlled trial of a good practice approach to treatment of adolescent obesity in Kuwait**. *Trial* 2014, **15**:234.

6. Brown B, Noonan C, Harris K, Parker M, Gaskill S, Ricci C, Cobbs G, Gress S: **Developing and piloting the Journey to Native Youth Health program in Northern Plains Indian communities**. *The Diabetes educator* 2013, **39**(1):109-118.

7. Cao Z, Wang S, Chen Y: **A randomized trial of multiple interventions for childhood obesity in China**. *Am J Prev Med* 2015, **48**(5):552-560.

8. Chen J, Weiss S, Heyman M, Lustig R: **Efficacy of a child-centred and family-based program in promoting healthy weight and healthy behaviors in Chinese American children: a randomized controlled study**. *Journal of public health* 2010, **32**(2):219-229.

9. Christiansen L, Toftager M, Boyle E, Kristensen P, Troelsen J: **Effect of a school environment intervention on adolescent adiposity and physical fitness**. *Scandinavian journal of medicine science in sports* 2013, **23**(6):e381-389.

10. Christiansen LB, Toftager M, Boyle E, Kristensen PL, Troelsen J: **Effect of a school environment intervention on adolescent adiposity and physical fitness**. *Scand J Med Sci Sports* 2013, **23**(6):e381-389.

11. Croker H, Viner R, Nicholls D, Haroun D, Chadwick P, Edwards C, Wells J, Wardle J: **Family-based behavioural treatment of childhood obesity in a UK National Health Service setting: randomized controlled trial**. *Int J Obes* 2012, **36**(1):16-26.

12. Davis A, Sampilo M, Gallagher K, Landrum Y, Malone B: **Treating rural pediatric obesity through telemedicine: outcomes from a small randomized controlled trial**. *J Pediatr Psychol* 2013, **38**(9):932-943.

13. Davis J, Tung A, Chak S, Ventura E, Byrd-Williams C, Alexander K, Lane C, Weigensberg M, Spruijt-Metz D, Goran M: **Aerobic and strength training reduces adiposity in overweight Latina adolescents**. *Medicine science in sports exercise* 2009, **41**(7):1494-1503.

14. Davoli A, Broccoli S, Bonvicini L, Fabbri A, Ferrari E, D'Angelo S, Di Buono A, Montagna G, Panza C, Pinotti M *et al*: **Pediatrician-led motivational interviewing to treat overweight children: an RCT**. *Pediatrics* 2013, **132**(5):e1236-1246.

15. de Heer H, Koehly L, Pederson R, Morera O: **Effectiveness and spillover of an after-school health promotion program for Hispanic elementary school children**. *Am J Public Health* 2011, **101**(10):1907-1913.

16. de Niet J, Timman R, Bauer S, van den Akker E, Buijks H, de Klerk C, Kordy H, Passchier J: **The effect of a short message service maintenance treatment on body mass index and psychological well-being in overweight and obese children: a randomized controlled trial**. *Pediatr Obes* 2012, **7**(3):205-219.

17. DeBar L, Stevens V, Perrin N, Wu P, Pearson J, Yarborough B, Dickerson J, Lynch F: **A primary care-based, multicomponent lifestyle intervention for overweight adolescent females**. *Pediatrics* 2012, **129**(3):e611-620.

18. Dewar D, Morgan P, Plotnikoff R, Okely A, Collins C, Batterham M, Callister R, Lubans D: **The nutrition and enjoyable activity for teen girls study: a cluster randomized controlled trial**. *Am J Prev Med* 2013, **45**(3):313-317.

19. Díaz R, Esparza-Romero J, Moya-Camarena S, Robles-Sardín A, Valencia M: **Lifestyle intervention in primary care settings improves obesity parameters among Mexican youth**. *J Am Diet Assoc* 2010, **110**(2):285-290.

20. Ebbeling C, Feldman H, Osganian S, Chomitz V, Ellenbogen S, Ludwig D: **Effects of decreasing sugar-sweetened beverage consumption on body weight in adolescents: a randomized, controlled pilot study**. *Pediatrics* 2006, **117**(3):673-680.

21. Ezendam N, Brug J, Oenema A: **Evaluation of the Web-based computer-tailored FATaintPHAT intervention to promote energy balance among adolescents: results from a school cluster randomized trial**. *Archives of pediatrics adolescent medicine* 2012, **166**(3):248-255.

22. Ford A, Bergh C, Södersten P, Sabin M, Hollinghurst S, Hunt L, Shield J: **Treatment of childhood obesity by retraining eating behaviour: randomised controlled trial**. *BMJ* 2009, **340**:b5388.

23. Gentile D, Welk G, Eisenmann J, Reimer R, Walsh D, Russell D, Callahan R, Walsh M, Strickland S, Fritz K: **Evaluation of a multiple ecological level child obesity prevention program: Switch what you Do, View, and Chew**. *BMC Med* 2009, **7**:49.

24. Gong L, Yuan F, Teng J, Li X, Zheng S, Lin L, Deng H, Ma G, Sun C, Li Y: **Weight loss, inflammatory markers, and improvements of iron status in overweight and obese children**. *The Journal of pediatrics* 2014, **164**(4):795-800.e792.

25. Gourlan M, Sarrazin P, Trouilloud D: **Motivational interviewing as a way to promote physical activity in obese adolescents: a randomised-controlled trial using self-determination theory as an explanatory framework**. *Psychology health Technology Assessment* 2013, **28**(11):1265-1286.

26. Grey M, Jaser S, Holl M, Jefferson V, Dziura J, Northrup V: **A multifaceted school-based intervention to reduce risk for type 2 diabetes in at-risk youth**. *Prev Med* 2009, **49**:122-128.

27. Grydeland M, Bjelland M, Anderssen S, Klepp K, Bergh I, Andersen L, Ommundsen Y, Lien N: **Effects of a 20-month cluster randomised controlled school-based intervention trial on BMI of school-aged boys and girls: the HEIA study**. *Br J Sports Med* 2014, **48**(9):768-773.

28. Hofsteenge G, Chinapaw M, Delemarre-van de Waal H, Weijs P: **Long-term effect of the Go4it group treatment for obese adolescents: a randomised controlled trial**. *Clin Nutr* 2014, **33**(3):385-391.

29. Howe C, Harris R, Gutin B: **A 10-month physical activity intervention improves body composition in young black boys**. *J Obes* 2011, **2011**:358581.

30. Hrafnkelsson H, Magnusson K, Thorsdottir I, Johannsson E, Sigurdsson E: **Result of school-based intervention on cardiovascular risk factors**. *Scand J Prim Health Care* 2014, **32**(4):149-155.

31. Hughes A, Stewart L, Chapple J, McColl J, Donaldson M, Kelnar C, Zabihollah M, Ahmed F, Reilly J: **Randomized, controlled trial of a best-practice individualized behavioral program for treatment of childhood overweight: Scottish Childhood Overweight Treatment Trial (SCOTT)**. *Pediatrics* 2008, **121**(3):e539-546.

32. James J, Thomas P, Cavan D, Kerr D: **Preventing childhood obesity by reducing consumption of carbonated drinks: cluster randomised controlled trial**. *BMJ* 2004, **328**(7450):1237.

33. Janicke D, Sallinen B, Perri M, Lutes L, Huerta M, Silverstein J, Brumback B: **Comparison of parent-only vs family-based interventions for overweight children in underserved rural settings: outcomes from project STORY**. *Archives of pediatrics adolescent medicine* 2008, **162**(12):1119-1125.

34. Jansen W, Borsboom G, Meima A, Zwanenburg E, Mackenbach J, Raat H, Brug J: **Effectiveness of a primary school-based intervention to reduce overweight**. *International journal of pediatric obesity : IJPO : an official journal of the International Association for the Study of Obesity* 2011, **6**:e70-77.

35. Jiang J, Xia X, Greiner T, Lian G, Rosenqvist U: **A two year family based behaviour treatment for obese children**. *Arch Dis Child* 2005, **90**(12):1235-1238.

36. Johnston C, Moreno J, El-Mubasher A, Gallagher M, Tyler C, Woehler D: **Impact of a school-based pediatric obesity prevention program facilitated by health professionals**. *The Journal of school health* 2013, **83**(3):171-181.

37. Johnston C, Tyler C, Fullerton G, Poston W, Haddock C, McFarlin B, Reeves R, Foreyt J: **Results of an intensive school-based weight loss program with overweight Mexican American children**. *International journal of pediatric obesity : IJPO : an official journal of the International Association for the Study of Obesity* 2007, **2**(3):144-152.

38. Kain J, Concha F, Moreno L, Leyton B: **School-based obesity prevention intervention in Chilean children: effective in controlling, but not reducing obesity**. *J Obes* 2014, **2014**:618293.

39. Kalarchian M, Levine M, Arslanian S, Ewing L, Houck P, Cheng Y, Ringham R, Sheets C, Marcus M: **Family-based treatment of severe pediatric obesity: randomized, controlled trial**. *Pediatrics* 2009, **124**(4):1060-1068.

40. Kalavainen M, Korppi M, Nuutinen O: **Clinical efficacy of group-based treatment for childhood obesity compared with routinely given individual counseling**. *Int J Obes* 2007, **31**(10):1500-1508.

41. Kalavainen M, Utriainen P, Vanninen E, Korppi M, Nuutinen O: **Impact of childhood obesity treatment on body composition and metabolic profile**. *World J Pediatr* 2012, **8**(1):31-37.

42. Kitzman-Ulrich H, Hampson R, Wilson D, Presnell K, Brown A, O'Boyle M: **An adolescent weight-loss program integrating family variables reduces energy intake**. *J Am Diet Assoc* 2009, **109**(3):491-496.

43. Klesges R, Obarzanek E, Kumanyika S, Murray D, Klesges L, Relyea G, Stockton M, Lanctot J, Beech B, McClanahan B *et al*: **The Memphis Girls' health Enrichment Multi-site Studies (GEMS): an evaluation of the efficacy of a 2-year obesity prevention program in African American girls**. *Archives of pediatrics adolescent medicine* 2010, **164**(11):1007-1014.

44. Kriemler S, Zahner L, Schindler C, Meyer U, Hartmann T, Hebestreit H, Brunner-La Rocca H, van Mechelen W, Puder J: **Effect of school based physical activity programme (KISS) on fitness and adiposity in primary schoolchildren: cluster randomised controlled trial**. *BMJ* 2010, **340**:c785.

45. Kriemler S, Zahner L, Schindler C, Meyer U, Hartmann T, Hebestreit H, Brunner-La Rocca HP, van Mechelen W, Puder JJ: **Effect of school based physical activity programme (KISS) on fitness and adiposity in primary schoolchildren: cluster randomised controlled trial**. *BMJ* 2010, **340**:c785.

46. Li Y, Hu X, Schouten E, Liu A, Du S, Li L, Cui Z, Wang D, Kok F, Hu F *et al*: **Report on childhood obesity in China (8): effects and sustainability of physical activity intervention on body composition of Chinese youth**. *Biomedical environmental sciences : BES* 2010, **23**(3):180-187.

47. Li YP, Hu XQ, Schouten EG, Liu AL, Du SM, Li LZ, Cui ZH, Wang D, Kok FJ, Hu FB *et al*: **Report on childhood obesity in China (8): effects and sustainability of physical activity intervention on body composition of Chinese youth**. *Biomed Environ Sci* 2010, **23**(3):180-187.

48. Llargues E, Franco R, Recasens A, Nadal A, Vila M, Pérez M, Manresa J, Recasens I, Salvador G, Serra J *et al*: **Assessment of a school-based intervention in eating habits and physical activity in school children: the AVall study**. *Journal of epidemiology community health* 2011, **65**(10):896-901.

49. Llargués E, Recasens A, Franco R, Nadal A, Vila M, Pérez M, Recasens I, Salvador G, Serra J, Roure E *et al*: **Medium-term evaluation of an educational intervention on dietary and physical exercise habits in schoolchildren: the Avall 2 study**. *Endocrinologia y nutricion : organo de la Sociedad Espanola de Endocrinologia y Nutricion* 2012, **59**(5):288-295.

50. Lubans D, Morgan P, Aguiar E, Callister R: **Randomized controlled trial of the Physical Activity Leaders (PALs) program for adolescent boys from disadvantaged secondary schools**. *Prev Med* 2011, **52**:239-246.

51. Lubans D, Morgan P, Callister R: **Potential moderators and mediators of intervention effects in an obesity prevention program for adolescent boys from disadvantaged schools**. *Journal of science medicine in sport* 2012, **15**(6):519-525.

52. Lubans D, Morgan P, Okely A, Dewar D, Collins C, Batterham M, Callister R, Plotnikoff R: **Preventing Obesity Among Adolescent Girls: One-Year Outcomes of the Nutrition and Enjoyable Activity for Teen Girls (NEAT Girls) Cluster Randomized Controlled Trial**. *Archives of pediatrics adolescent medicine* 2012, **166**(9):821-827.

53. Lubans D, Smith J, Plotnikoff R, Dally K, Okely A, Salmon J, Morgan P: **Assessing the sustained impact of a school-based obesity prevention program for adolescent boys: the ATLAS cluster randomized controlled trial**. *The international journal of behavioral nutrition physical activity* 2016, **13**:92.

54. Macias-Cervantes M, Malacara J, Garay-Sevilla M, Díaz-Cisneros F: **Effect of recreational physical activity on insulin levels in Mexican/Hispanic children**. *Eur J Pediatr* 2009, **168**(10):1195-1202.

55. Macias-Cervantes MH, Malacara JM, Garay-Sevilla ME, Díaz-Cisneros FJ: **Effect of recreational physical activity on insulin levels in Mexican/Hispanic children**. *Eur J Pediatr* 2009, **168**(10):1195-1202.

56. Maddison R, Marsh S, Foley L, Epstein L, Olds T, Dewes O, Heke I, Carter K, Jiang Y, Mhurchu C: **Screen-Time Weight-loss Intervention Targeting Children at Home (SWITCH): a randomized controlled trial**. *The international journal of behavioral nutrition physical activity* 2014, **11**:111.

57. Magnusson K, Hrafnkelsson H, Sigurgeirsson I, Johannsson E, Sveinsson T: **Limited effects of a 2-year school-based physical activity intervention on body composition and cardiorespiratory fitness in 7-year-old children**. *Health Educ Res* 2012, **27**(3):484-494.

58. Martínez Vizcaíno V, Salcedo Aguilar F, Franquelo Gutiérrez R, Solera Martínez M, Sánchez López M, Serrano Martínez S, López García E, Rodríguez Artalejo F: **Assessment of an after-school physical activity program to prevent obesity among 9- to 10-year-old children: a cluster randomized trial**. *Int J Obes* 2008, **32**(1):12-22.

59. Martínez-Vizcaíno V, Sánchez-López M, Notario-Pacheco B, Salcedo-Aguilar F, Solera-Martínez M, Franquelo-Morales P, López-Martínez S, García-Prieto J, Arias-Palencia N, Torrijos-Niño C *et al*: **Gender differences on effectiveness of a school-based physical activity intervention for reducing cardiometabolic risk: a cluster randomized trial**. *The international journal of behavioral nutrition physical activity* 2014, **11**:154.

60. McCallum Z, Wake M, Gerner B, Baur L, Gibbons K, Gold L, Gunn J, Harris C, Naughton G, Riess C *et al*: **Outcome data from the LEAP (Live, Eat and Play) trial: a randomized controlled trial of a primary care intervention for childhood overweight/mild obesity**. *Int J Obes* 2007, **31**(4):630-636.

61. Meng L, Xu H, Liu A, van Raaij J, Bemelmans W, Hu X, Zhang Q, Du S, Fang H, Ma J *et al*: **The costs and cost-effectiveness of a school-based comprehensive intervention study on childhood obesity in China**. *PLoS One* 2013, **8**(10):e77971.

62. Mihas C, Mariolis A, Manios Y, Naska A, Arapaki A, Mariolis-Sapsakos T, Tountas Y: **Evaluation of a nutrition intervention in adolescents of an urban area in Greece: short- and long-term effects of the VYRONAS study**. *Public Health Nutr* 2010, **13**(5):712-719.

63. Morgan P, Lubans D, Callister R, Okely A, Burrows T, Fletcher R, Collins C: **The 'Healthy Dads, Healthy Kids' randomized controlled trial: efficacy of a healthy lifestyle program for overweight fathers and their children**. *Int J Obes* 2011, **35**(3):436-447.

64. Morgan P, Saunders K, Lubans D: **Improving physical self-perception in adolescent boys from disadvantaged schools: psychological outcomes from the Physical Activity Leaders randomized controlled trial**. *Pediatr Obes* 2012, **7**(3):e27-32.

65. Muckelbauer R, Libuda L, Clausen K, Toschke A, Reinehr T, Kersting M: **Promotion and provision of drinking water in schools for overweight prevention: randomized, controlled cluster trial**. *Pediatrics* 2009, **123**(4):e661-667.

66. Nemet D, Barkan S, Epstein Y, Friedland O, Kowen G, Eliakim A: **Short- and long-term beneficial effects of a combined dietary-behavioral-physical activity intervention for the treatment of childhood obesity**. *Pediatrics* 2005, **115**(4):e443-449.

67. Nguyen B, Shrewsbury V, O'Connor J, Steinbeck K, Lee A, Hill A, Shah S, Kohn M, Torvaldsen S, Baur L: **Twelve-month outcomes of the loozit randomized controlled trial: a community-based healthy lifestyle program for overweight and obese adolescents**. *Archives of pediatrics adolescent medicine* 2012, **166**(2):170-177.

68. Nollen N, Mayo M, Carlson S, Rapoff M, Goggin K, Ellerbeck E: **Mobile technology for obesity prevention: a randomized pilot study in racial- and ethnic-minority girls**. *Am J Prev Med* 2014, **46**(4):404-408.

69. Paineau D, Beaufils F, Boulier A, Cassuto D, Chwalow J, Combris P, Couet C, Jouret B, Lafay L, Laville M *et al*: **Family dietary coaching to improve nutritional intakes and body weight control: a randomized controlled trial**. *Archives of pediatrics adolescent medicine* 2008, **162**(1):34-43.

70. Pakpour A, Gellert P, Dombrowski S, Fridlund B: **Motivational interviewing with parents for obesity: an RCT**. *Pediatrics* 2015, **135**(3):e644-652.

71. Pbert L, Druker S, Gapinski M, Gellar L, Magner R, Reed G, Schneider K, Osganian S: **A school nurse-delivered intervention for overweight and obese adolescents**. *The Journal of school health* 2013, **83**(3):182-193.

72. Peralta L, Jones R, Okely A: **Promoting healthy lifestyles among adolescent boys: the Fitness Improvement and Lifestyle Awareness Program RCT**. *Prev Med* 2009, **48**(6):537-542.

73. Prado D, Silva A, Trombetta I, Ribeiro M, Guazzelli I, Matos L, Santos M, Nicolau C, Negrão C, Villares S: **Exercise training associated with diet improves heart rate recovery and cardiac autonomic nervous system activity in obese children**. *Int J Sports Med* 2010, **31**(12):860-865.

74. Reed K, Warburton D, Macdonald H, Naylor P, McKay H: **Action Schools! BC: a school-based physical activity intervention designed to decrease cardiovascular disease risk factors in children**. *Prev Med* 2008, **46**(6):525-531.

75. Reinehr T, Schaefer A, Winkel K, Finne E, Toschke A, Kolip P: **An effective lifestyle intervention in overweight children: findings from a randomized controlled trial on "Obeldicks light"**. *Clin Nutr* 2010, **29**(3):331-336.

76. Robbins L, Gretebeck K, Kazanis A, Pender N: **Girls on the move program to increase physical activity participation**. *Nurs Res* 2006, **55**(3):206-216.

77. Robinson T, Killen J, Kraemer H, Wilson D, Matheson D, Haskell W, Pruitt L, Powell T, Owens A, Thompson N *et al*: **Dance and reducing television viewing to prevent weight gain in African-American girls: the Stanford GEMS pilot study**. *Ethnicity disease* 2003, **13**:S65-77.

78. Rosário R, Oliveira B, Araújo A, Lopes O, Padrão P, Moreira A, Teixeira V, Barros R, Pereira B, Moreira P: **The impact of an intervention taught by trained teachers on childhood overweight**. *International journal of environmental research public health nutrition* 2012, **9**(4):1355-1367.

79. Sacher P, Kolotourou M, Chadwick P, Cole T, Lawson M, Lucas A, Singhal A: **Randomized controlled trial of the MEND program: a family-based community intervention for childhood obesity**. *Obesity* 2010:S62-68.

80. Saelens B, Grow H, Stark L, Seeley R, Roehrig H: **Efficacy of increasing physical activity to reduce children's visceral fat: a pilot randomized controlled trial**. *International journal of pediatric obesity : IJPO : an official journal of the International Association for the Study of Obesity* 2011, **6**(2):102-112.

81. Saelens B, Sallis J, Wilfley D, Patrick K, Cella J, Buchta R: **Behavioral weight control for overweight adolescents initiated in primary care**. *Obes Res* 2002, **10**(1):22-32.

82. Safdie M, Jennings-Aburto N, Lévesque L, Janssen I, Campirano-Núñez F, López-Olmedo N, Aburto T, Rivera J: **Impact of a school-based intervention program on obesity risk factors in Mexican children**. *Salud Publica Mex* 2013:374-387.

83. Santos R, Durksen A, Rabbanni R, Chanoine J, Lamboo Miln A, Mayer T, McGavock J: **Effectiveness of peer-based healthy living lesson plans on anthropometric measures and physical activity in elementary school students: a cluster randomized trial**. *JAMA pediatrics* 2014, **168**(4):330-337.

84. Savoye M, Caprio S, Dziura J, Camp A, Germain G, Summers C, Li F, Shaw M, Nowicka P, Kursawe R *et al*: **Reversal of early abnormalities in glucose metabolism in obese youth: results of an intensive lifestyle randomized controlled trial**. *Diabetes Care* 2014, **37**(2):317-324.

85. Savoye M, Nowicka P, Shaw M, Yu S, Dziura J, Chavent G, O'Malley G, Serrecchia J, Tamborlane W, Caprio S: **Long-term results of an obesity program in an ethnically diverse pediatric population**. *Pediatrics* 2011, **127**(3):402-410.

86. Savoye M, Shaw M, Dziura J, Tamborlane W, Rose P, Guandalini C, Goldberg-Gell R, Burgert T, Cali A, Weiss R *et al*: **Effects of a weight management program on body composition and metabolic parameters in overweight children: a randomized controlled trial**. *JAMA* 2007, **297**(24):2697-2704.

87. Shaw M, Savoye M, Cali A, Dziura J, Tamborlane W, Caprio S: **Effect of a successful intensive lifestyle program on insulin sensitivity and glucose tolerance in obese youth**. *Diabetes Care* 2009, **32**(1):45-47.

88. Shelton D, Le Gros K, Norton L, Stanton-Cook S, Morgan J, Masterman P: **Randomised controlled trial: A parent-based group education programme for overweight children**. *Journal of paediatrics child health* 2007, **43**(12):799-805.

89. Sichieri R, Paula Trotte A, de Souza R, Veiga G: **School randomised trial on prevention of excessive weight gain by discouraging students from drinking sodas**. *Public Health Nutr* 2009, **12**(2):197-202.

90. Siegrist M, Lammel C, Haller B, Christle J, Halle M: **Effects of a physical education program on physical activity, fitness, and health in children: the JuvenTUM project**. *Scandinavian journal of medicine science in sports* 2013, **23**(3):323-330.

91. Singh A, Chin A Paw M, Brug J, van Mechelen W: **Dutch obesity intervention in teenagers: effectiveness of a school-based program on body composition and behavior**. *Archives of pediatrics adolescent medicine* 2009, **163**(4):309-317.

92. Smith J, Morgan P, Plotnikoff R, Dally K, Salmon J, Okely A, Finn T, Lubans D: **Smart-phone obesity prevention trial for adolescent boys in low-income communities: the ATLAS RCT**. *Pediatrics* 2014, **134**(3):e723-731.

93. Story M, Sherwood N, Himes J, Davis M, Jacobs D, Cartwright Y, Smyth M, Rochon J: **An after-school obesity prevention program for African-American girls: the Minnesota GEMS pilot study**. *Ethnicity disease* 2003, **13**:S54-64.

94. Tsiros M, Sinn N, Brennan L, Coates A, Walkley J, Petkov J, Howe P, Buckley J: **Cognitive behavioral therapy improves diet and body composition in overweight and obese adolescents**. *The American journal of clinical nutrition* 2008, **87**(5):1134-1140.

95. Velez A, Golem D, Arent S: **The impact of a 12-week resistance training program on strength, body composition, and self-concept of Hispanic adolescents**. *Journal of strength conditioning research* 2010, **24**(4):1065-1073.

96. Vos R, Wit J, Pijl H, Houdijk E: **Long-term effect of lifestyle intervention on adiposity, metabolic parameters, inflammation and physical fitness in obese children: a randomized controlled trial**. *Nutrition diabetes care* 2011, **1**:e9.

97. Wafa S, Talib R, Hamzaid N, McColl J, Rajikan R, Ng L, Ramli A, Reilly J: **Randomized controlled trial of a good practice approach to treatment of childhood obesity in Malaysia: Malaysian Childhood Obesity Treatment Trial (MASCOT)**. *International journal of pediatric obesity : IJPO : an official journal of the International Association for the Study of Obesity* 2011, **6**:e62-69.

98. Wake M, Baur L, Gerner B, Gibbons K, Gold L, Gunn J, Levickis P, McCallum Z, Naughton G, Sanci L *et al*: **Outcomes and costs of primary care surveillance and intervention for overweight or obese children: the LEAP 2 randomised controlled trial**. *BMJ* 2009, **339**:b3308.

99. Waling M, Lind T, Hernell O, Larsson C: **A one-year intervention has modest effects on energy and macronutrient intakes of overweight and obese Swedish children**. *The Journal of nutrition* 2010, **140**(10):1793-1798.

100. Weeks B, Beck B: **Twice-weekly, in-school jumping improves lean mass, particularly in adolescent boys**. *Pediatr Obes* 2012, **7**(3):196-204.

101. Williamson D, Martin P, White M, Newton R, Walden H, York-Crowe E, Alfonso A, Gordon S, Ryan D: **Efficacy of an internet-based behavioral weight loss program for overweight adolescent African-American girls**. *Eating weight disorders : EWD* 2005, **10**(3):193-203.

102. Wright K, Suro Z: **Using community--academic partnerships and a comprehensive school-based program to decrease health disparities in activity in school-aged children**. *Journal of prevention intervention in the community* 2014, **42**(2):125-139.

103. Ochoa-Avilés A, Verstraeten R, Huybregts L, Andrade S, Van Camp J, Donoso S, Ramírez PL, Lachat C, Maes L, Kolsteren P: **A school-based intervention improved dietary intake outcomes and reduced waist circumference in adolescents: a cluster randomized controlled trial**. *Nutrition journal* 2017, **16**(1):79.

104. Kim HS, Park J, Park KY, Lee MN, Ham OK: **Parent Involvement Intervention in Developing Weight Management Skills for both Parents and Overweight/Obese Children**. *Asian Nurs Res (Korean Soc Nurs Sci)* 2016, **10**(1):11-17.

105. Ahmad N, Shariff ZM, Mukhtar F, Lye MS: **Family-based intervention using face-to-face sessions and social media to improve Malay primary school children's adiposity: a randomized controlled field trial of the Malaysian REDUCE programme**. *Nutrition journal* 2018, **17**.

106. Bai, Miranda, Pallan, Wei, Jia, Liu, Karla, Hemming, Emma, Frew: **The CHIRPY DRAGON intervention in preventing obesity in Chinese primary-school--aged children: Acluster-randomised controlled trial**. *PLoS Med* 2019, **16**(11):e1002971.

107. Bru?Ó A, Escobar P, Cebolla A, álvarez-Pitti J, Guixeres J, Lurbe E, Ba?Os R, Lisón JF: **Home-exercise Childhood Obesity Intervention: A Randomized Clinical Trial Comparing Print Versus Web-based (Move It) Platforms**. *J Pediatr Nurs* 2018:S0882596317307030.

108. Chen JL, Guedes CM, Lung AE: **Smartphone-Based Healthy Weight Management Intervention for Chinese American Adolescents: Short-Term Efficacy and Factors Associated With Decreased Weight**. *J Adolesc Health* 2018, **64**(4).

109. Hao M, Han W, Yamauchi T: **Short-Term and Long-Term Effects of a Combined Intervention of Rope Skipping and Nutrition Education for Overweight Children in Northeast China**. *Asia Pac J Public Health* 2019, **31**(4):101053951984827.

110. Jenny Lloyd Ph Da, Siobhan Creanor Cc, Prof Stuart Logan MDa, Prof Colin Green Ph Da, Sarah GDPa, Melvyn Hillsdon Ph Db, Prof Charles Abraham Ph Da, Richard Tomlinson MDd, Virginia Pearson MDe, Prof Rod STPa: **Effectiveness of the Healthy Lifestyles Programme (HeLP) to prevent obesity in UK primary-school children: a cluster randomised controlled trial**. *Lancet Child & Adolescent Health* 2018, **2**(1):35-45.

111. Larsen KT, Huang T, Riedlarsen M, Andersen LB, Heidemann M, Møller NC: **A Multi-Component Day-Camp Weight-Loss Program Is Effective in Reducing BMI in Children after One Year: A Randomized Controlled Trial**. *PLoS One* 2016, **11**(6):e0157182.

112. Liu Z, Li Q, Maddison R, Ni Mhurchu C, Jiang Y, Wei D-M, Cheng L, Cheng Y, Wang D, Wang H-J: **A School-Based Comprehensive Intervention for Childhood Obesity in China: A Cluster Randomized Controlled Trial**. *Childhood Obesity* 2018.

113. Md. Yusop NB, Mohd Shariff Z, Hwu TT, Abd. Talib R, Spurrier N: **The effectiveness of a stage-based lifestyle modification intervention for obese children**. *BMC Public Health* 2018, **18**(1):299.

114. Morell-Azanza L, Ojeda-Rodríguez A, Ochotorena-Elicegui A, Martín-Calvo N, Chueca M, Marti A, Julian AS: **Changes in objectively measured physical activity after a multidisciplinary lifestyle intervention in children with abdominal obesity: a randomized control trial**. *BMC Pediatr* 2019, **19**.

115. Nayak BS, Bhat VH: **School Based Multicomponent Intervention for Obese Children in Udupi District, South India  A Randomized Controlled Trial**. *Journal of Clinical & Diagnostic Research Jcdr* 2016, **10**(5):BC05.

116. Pbert L, Druker S, Barton B, Schneider KL, Olendzki B, Gapinski MA, Kurtz S, Osganian S: **A School-Based Program for Overweight and Obese Adolescents: A Randomized Controlled Trial**. *J Sch Health* 2016, **86**(10):699-708.

117. Staiano AE, Beyl RA, Guan W, Hendrick CA, Hsia DS, Newton RL: **Home-based exergaming among children with overweight and obesity: a randomized clinical trial: Home exergaming in children with obesity**. *Pediatr Obes* 2018, **13**.

118. Xu H, Li Y, Zhang Q, Hu X, Liu A, Du S, Li T, Guo H, Li Y, Xu G: **Comprehensive school-based intervention to control overweight and obesity in China: a cluster randomized controlled trial**. *Asia Pac J Clin Nutr* 2017, **26**.
