# Supplementary material for: Face-to-face physical activity incorporated into dietary intervention for overweight/obesity in children and adolescents: a Bayesian network meta-analysis
Source: BMC Med. 2022 Sep 2;20:325. doi: 10.1186/s12916-022-02462-6 (PMC9438135; doi:10.1186/s12916-022-02462-6)
Supplement: Supplementary file 2 — Additional file 2. INPLASY Protocol. [file 12916_2022_2462_MOESM2_ESM.pdf]

INPLASY PROTOCOL

To cite: Liang et al. Face-to-face Physical activity incorporated with Dietary intervention may be the optimal intervention strategy for preventing obesity among children and adolescents — Evidence from Bayesian Network Meta-analysis of 118 randomized controlled trials with 71,064 individuals. Inplasy protocol 202120072. doi: 10.37766/inplasy2021.2.0072

Received: 21 February 2021

Published: 21 February 2021

Corresponding author:
Yajun Chen

chenyj68@126.com

Author Affiliation:
School of public Health, Medical College of Sun Yat-sen University

Support: **NSFC81673139.**

Review Stage at time of this submission: **Data analysis.**

Conflicts of interest:
None declared.

Face-to-face Physical activity incorporated with Dietary intervention may be the optimal intervention strategy for preventing obesity among children and adolescents — Evidence from Bayesian Network Meta-analysis of 118 randomized controlled trials with 71,064 individuals

Liang, J[1]; Zhao, Y[2]; Chen, Y[3]; Zhang, S[4]; Tan, K[5]; Zhang, J[6]; Kakaer, A[7]; Chen, Y[8].

Review question / Objective: **Obesity has been highlighted to induce deleterious consequences among adolescents and be considered as one of the promising modifiable risk factors. We aimed at investigating the optimal intervention for obese and overweight children and adolescents.**
Condition being studied: **Several databases along with additional manual-search were implemented for identifying the relevant Randomized controlled trials(RCTs) that reported the association between various interventions and obese children and adolescents. A Bayesian network meta-analysis was conducted to summarize comparative effectiveness of 8 interventions based on the primary outcome.**

INPLASY registration number: This protocol was registered with the International Platform of Registered Systematic Review and Meta-Analysis Protocols (INPLASY) on 21 February 2021 and was last updated on 21 February 2021 (registration number INPLASY202120072).

INTRODUCTION

Review question / Objective: **Obesity has been highlighted to induce deleterious consequences among adolescents and be considered as one of the promising modifiable risk factors. We aimed at investigating the optimal intervention for**

Liang et al. Inplasy protocol 202120072. doi:10.37766/inplasy2021.2.0072 Downloaded from https://inplasy.com/inplasy-2021-2-0072/

obese and overweight children and adolescents.

Condition being studied: Several databases along with additional manual-search were implemented for identifying the relevant Randomized controlled trials(RCTs) that reported the association between various interventions and obese children and adolescents. A Bayesian network meta-analysis was conducted to summarize comparative effectiveness of 8 interventions based on the primary outcome.

METHODS

Search strategy: Without any limitation on language and publication data, two investigators independently screening several databases including Medline(via Pubmed), PsycINFO(via Ovid) Cochrane, Web of science, Embase, CINAHL, the Chinese Biomedical Literature Database, Clinical trials(www.clinicaltrials.gov) and the other meta-analysis search strategies for identifying eligible RCTs that investigated the association between various prevention strategies and obesity in children and adolescents from their inception to the end of December, 1st 2020. The following Medical subject headings[MeSH] terms and keywords items incorporating with Boolean operators were applied: “Children”, “Adolescents”, “Students”, “Youth”, “Treatment”, “Diet”, “Physical activity”, “Telemedicine”, “Healthy lifestyle”, “Obesity”, “Adiposity”, “Overweight” and “Randomized controlled trials”.

Participant or population: Studies recruited participants that were children or adolescents aged from 6 to 18 years old with their standardized diagnostic measures of obesity.

Intervention: Interventions were any type of PA(e.g., aerobic exercise, resistance training, endurance exercise), DI(e.g, Very low-carbohydrate diet, Very low-energy diet, Low-fat diet), MLI, or any the above mentioned interventions in combinatorial or multicomponent, whether they were delivered by Mobile-health(MH) technology or face-to-face approach.

Comparator: Comparators were various interventions themselves or Named control group(NCG) alone such as wait-list control group, treatment as usual.

Study designs to be included: Any type of RCTs whether they have designed into parallel or cross-over setting. Without any race, region, publication year and language restriction on the above items.

Eligibility criteria: (1) Interventions were any type of PA(e.g., aerobic exercise, resistance training, endurance exercise), DI(e.g, Very low-carbohydrate diet, Very low-energy diet, Low-fat diet), MLI, or any the above mentioned interventions in combinatorial or multicomponent, whether they were delivered by Mobile-health(MH) technology or FTF approach; (2) Studies recruited participants that were children or adolescents aged from 6 to 18 years old with their standardized diagnostic measures of obesity; (3) Comparators were various interventions themselves or Named control group(NCG) alone such as wait-list control group, treatment as usual; (4) Children with obesity or any prevalent subtypes, which was assessed by measurable instruments or the quantifiable indicators with quadratic transformation, such as Body Mass Index(BMI), BMI Z-score and Waist circumference(WC); (5) Any type of RCTs whether they have designed into parallel or cross-over setting.

Information sources: Without any limitation on language and publication data, two investigators independently screening several databases including Medline(via Pubmed), PsycINFO(via Ovid) Cochrane, Web of science, Embase, CINAHL, the Chinese Biomedical Literature Database, Clinical trials(www.clinicaltrials.gov) and the other meta-analysis search strategies for identifying eligible RCTs that investigated the association between various prevention strategies and obesity in children and adolescents from their inception to the end of December, 1st 2020. The following Medical subject

Liang et al. Inplasy protocol 202120072. doi:10.37766/inplasy2021.2.0072 Downloaded from https://inplasy.com/inplasy-2021-2-0072/

headings[MeSH] terms and keywords items incorporating with Boolean operators were applied: “Children”, “Adolescents”, “Students”, “Youth”, “Treatment”, “Diet”, “Physical activity”, “Telemedicine”, “Healthy lifestyle”, “Obesity”, “Adiposity”, “Overweight” and “Randomized controlled trials”. Further searches were conducted by hand-search which included screening the bibliographies of the relevantly published systematic review or meta-analysis, and executed a search review on the key journals, major conferences for omitting the qualified studies which may be missed at the initial search.

Main outcome(s): The pre-customized primary obesity-related outcomes were BMI and BMI Z-score.

Additional outcome(s): The secondary outcomes of interest were the Percent body fat(%PBF), WC.

Quality assessment / Risk of bias analysis: Quality of the included individual study was judged by two independent reviewers using Cochrane Collaboration Risk of Bias Tool(ROB), which consisted of seven items, namely, random sequence generation, allocation concealment, blinding of participants and personnel, blinding of outcome assessors, incomplete outcome data, incomplete outcome reporting, other bias. Low, unclear and high risk of bias were rated as quality grade of studies respectively. What is more, the GRADE system was considered for rating the summary of findings, as outlined in the GRADE handbook.

Strategy of data synthesis: The Bayesian hierarchical random effects were ever conducted to contrast diverse treatments, in which a connective network was generated and meanwhile direct and indirect assessment was integrated using the method of multivariate meta-analysis to compare the diverse treatment at the same time. Three Markov chains in parallel were created randomly at first to be a simulation of an exact appraisal in statistic modes. 50000 iterations were generated per chain, referring to the period of burn-in, when the chain came to its eventual distribution, in order to make the beginning figure deviate at a minimum, the former 10000 iterations were given up. The model convergence was estimated by the Brooks-Gelman-Rubin diagnostic, in which the historical trajectory was observed directly combining the trace plot and the density plot. As an appraisable likelihood which gave grade of treatments in target, the surface under the cumulative ranking curve(SUCRA) summarized individual treatment by providing a brief numerical statistic cumulative ranking probability plot. If the value of SUCRA is higher, it’s more probable that the provided treatment ranks or takes effect at the top, on the contrary, if it’s zero, the treatment gets the worst result. The “node-splitting” technique was used to clarify the existence possibility of an underlying source difference contrasting the evidence direct and indirect from all the network (consistency appears when P-value surpasses 0.05).

Subgroup analysis: Sequential of areas were considered into our model shown below: Grade(Children vs. Adolescents); Region (Developed countries vs. developing & undeveloped countries); Publication year (Year ≥2010 vs. Year< 2010); Treatment cycle(Duration weeks≥24 vs. Duration weeks<24); Boy to girl ratio(Ratio≥1 vs. Ratio<1); Total sample size(Sample size ≥100 vs. Sample size< 100); Intervention site(School vs. Family); Treatment setting (Group vs. Individual). Discrepancies generated during the analyses process were reconciled through consultation or equitable equitable judgement by an experienced author.

Sensitivity analysis: NA.

Country(ies) involved: China.

Keywords: Multiple interventions, Obesity, Children and adolescents, Bayesian network meta-analysis.

Contributions of each author:

Author 1 - Jinghong Liang.
Email: liangjh78@mail2.sysu.edu.cn

Liang et al. Inplasy protocol 202120072. doi:10.37766/inplasy2021.2.0072 Downloaded from https://inplasy.com/inplasy-2021-2-0072/

Author 2 - Yu Zhao.
Email: zhaoy369@mail2.sysu.edu.cn
Author 3 - Yican Chen.
Email: chenyc55@mail2.sysu.edu.cn
Author 4 - Shuxin Zhang.
Email: zhangshx26@mail2.sysu.edu.cn
Author 5 - Kaiyun Tan.
Email: tanky3@mail2.sysu.edu.cn
Author 6 - Jingshu Zhang.
Email: zhangjsh29@mail2.sysu.edu.cn
Author 7 - Aerziguli Kakaer.
Email: aezglkke@mail2.sysu.edu.cn
Author 8 - Yajun Chen.
Email: chenyj68@126.com

Liang et al. Inplasy protocol 202120072. doi:10.37766/inplasy2021.2.0072 Downloaded from https://inplasy.com/inplasy-2021-2-0072/
