# Supplementary material for: Face-to-face physical activity incorporated into dietary intervention for overweight/obesity in children and adolescents: a Bayesian network meta-analysis
Source: BMC Med. 2022 Sep 2;20:325. doi: 10.1186/s12916-022-02462-6 (PMC9438135; doi:10.1186/s12916-022-02462-6)
Supplement: Supplementary file 3 — Additional file 3: Table S1. Baseline characteristics of the 118 included trials; Table. S2. Relative effect sizes of treatments efficacy at post-treatment based on network meta-analysis (WC and PBF outcomes); Table. S3-6. Subgroup analyses for varied treatments based on the outcome of BMI; Table. S7-8. Sensitivity analyses of outcomes assessment of interventions duration for varied treatments based on the outcome of BMI; Table. S9-12. The confidence in MD for mean overall change in four outcomes by GRADE system; Table. S13. Risk of bias for individual study. [file 12916_2022_2462_MOESM3_ESM.docx]

**Table S1 Baseline characteristics of the 118 included trials**

| Publications | Treatment and sample size | Inclusion criteria | Age(mean±sd/range) | | Proportion of Boy(%) | Treatment period(weeks/months) | Format | Setting | Outcome | Type of RCT | Region |
| --- | --- | --- | --- | --- | --- | --- | --- | --- | --- | --- | --- |
|  |  |  | IG | NCG |  |  |  |  |  |  |  |
| Christiansen, 2013 | FTF-PA=623 vs. NCG=725 | Adiposity | 12.60 ±0.60 | 12.60 ± 0.60 | 51.56% | 2Ys | School | Group | C | Cluster | Denmark |
| Macias-Cervantes, 2009 | FTF-PA=38 vs. NCG=38 | Overweight or obesity | 7.50 ± 0.75 | 8.00 ± 0.38 | 56.46% | 12Ws | School | NR | A, C | NR | Mexico |
| Kriemler, 2010 | FTF-PA=297 vs. NCG=205 | Adiposity | 6.90 ± 0.30 | 6.90 ± 0.30 | NR | 1Y | School | NR | A, C | Cluster | Switzerland |
| Li, 2010 | FTF-PA=2329 vs. NCG=2371 | Obesity | 9.30 ± 0.70 | | 52.07% | 1Y | School | Group | A, B, C | Cluster | China |
| Lubans, 2011 | FTF-PA=50 vs. NCG=50 | Overweight or obesity | 14.40 ± 0.70 | 14.20 ± 0.40 | 100.00% | 6Ms | School | NR | A, B, C, D | NR | Australia |
| Reed, 2008 | FTF-PA=178 vs. NCG=90 | NR | NR | | NR | 9Ms | School | Group | A | Cluster | UK |
| Velez, 2010 | FTF-PA=15 vs. NCG=16 | Overweight or obesity | 16.14 ± 0.19 | | 57.14% | 12Ws | School | NR | A,D | NR | US |
| Weeks, 2012 | FTF-PA=52 vs. NCG=47 | Overweight or obesity | 13.80 ± 0.40 | | 45.68% | 8Ms | School | NR | A,D | NR | Australia |
| Howe, 2010 | FTF-PA=31 vs. NCG=44 | Overweight or obesity | 9.70 ± 0.20 | 9.80 ± 0.20 | 62.78% | 10Ms | School | NR | A, C, D | NR | Georgia |
| Andrade, 2014 | FTF-PA=700 vs. NCG=740 | NR | 12.90 ± 0.80 | 12.90 ± 0.80 | 62.78% | 24Ms | School | NR | B | Cluster | Spain |
| Staiano, 2018 | FTF-PA=23 vs. NCG=23 | Overweight or obesity | 11.20 ± 0.80 | | 54.35% | 24Ws | Family | NR | D | Parallel | US |
| Peralta, 2009 | FTF-PA=16 vs. NCG=17 | Obesity | 12.50 ± 0.40 | | 100.00% | 6Ms | School | Group | A, C, D | Parallel | Australia |
| Robbins, 2006* | FTF-PA=22 vs. NCG=8 | NR | 11.45 ± 0.80 | 11.25± 0.46 | 0.00% | 12Ws | School | Individual | A | NR | US |
| Robbins, 2006* | FTF-PA=19 vs. NCG=15 | NR | 12.37 ± 0.50 | 12.27 ± 0.59 | 0.00% | 12Ws | School | Individual | A | NR | US |
| Robbins, 2006* | FTF-PA=4 vs. NCG=9 | NR | 13.00 ± 0.00 | 13.44 ± 0.53 | 0.00% | 12Ws | School | Individual | A | NR | US |
| Vizcaíno, 2008* | FTF-PA=260 vs. NCG=296 | Obesity | 9.40 ± 0.70 | 9.50 ± 0.70 | 100.00% | 24Ws | School | Group | A, D | Cluster | Spain |
| Vizcaíno, 2008* | FTF-PA=253 vs. NCG=310 | Obesity | 9.40 ± 0.70 | 9.40 ± 0.60 | 0.00% | 24Ws | School | Group | A, D | Cluster | Spain |
| Martínez-Vizcaíno, 2014* | FTF-PA=232 vs. NCG=307 | Overweight or obesity | 9.40 ± 0.70 | 9.50 ± 0.70 | 100.00% | 1Y | School | NR | A, C, D | Cluster | Spain |
| Martínez-Vizcaíno, 2014* | FTF-PA=257 vs. NCG=274 | Overweight or obesity | 9.40 ± 0.70 | 9.50 ± 0.70 | 0.00% | 1Y | School | NR | A, C, D | Cluster | Spain |
| Mihas, 2009 | FTF-DI=108 vs. NCG=105 | Obesity | 13.10 ± 0.80 | 13.30 ± 0.90 | 51.31% | 12Ws | School | NR | A | NR | Greece |
| Rosário, 2012 | FTF-DI=233 vs. NCG=231 | Overweight or obesity | 8.30 ± 1.20 | 8.20 ± 1.20 | 48.49% | 6Ms | School | NR | B | NR | Portugal |
| Sichieri, 2009 | FTF-DI=526 vs. NCG=608 | Overwight | 10.90 ± 0.81 | 10.90 ± 0.75 | NR | 7Ms | School | NR | A | Cluster | Brazil |
| Ebbeling, 2006 | FTF-DI=53 vs. NCG=50 | NR | 16.00 ± 1.10 | 15.80 ± 1.10 | 45.63% | 25Ws | Family | Individual | A | NR | US |
| James, 2004 | FTF-DI=325 vs. NCG=319 | Obesity | 8.70 ± 1.00 | 8.70 ± 0.90 | 50.32% | 12Ms | School | NR | A | Cluster | UK |
| Muckelbauer, 2009 | FTF-DI=1978 vs. NCG=1839 | Overwight | 8.26 ± 0.73 | 8.34 ± 0.76 | 50.24% | 1Y | School | NR | B | Cluster | Germany |
| Ochoa-Avilés, 2017 | FTF-DI=702 vs. NCG=728 | NR | 12.90 ± 0.80 | 12.90 ± 0.80 | 37.76% | 17Ms | School | Group | C | Cluster | Ecuador |
| Black, 2010 | FTF-PA+DI=96 vs. NCG=37 | obesity | 13.30 ± 1.00 | 13.30 ± 1.00 | 51.49% | 10Ms | NR | NR | B | NR | US |
| Bohnert, 2013 | FTF-PA+DI=96 vs. NCG=37 | NR | 9.02 ± 0.93 | 9.38 ± 1.13 | 0.00% | 30Ms | School | Group | B | NR | US |
| Brown, 2013 | FTF-PA+DI=38 vs. NCG=38 | Overweight or obesity | 10-14 | | NR | 12Ws | NR | Individual | A,B | NR | US |
| Cao, 2015 | FTF-PA+DI=965 vs. NCG=889 | Obesity | 7.01 ± 0.44 | 6.81 ± 0.24 | 52.99% | 10Ms | NR | Individual | B | Culster | China |
| Chen, 2010 | FTF-PA+DI=35 vs. NCG=32 | NR | 9.14 ± 0.85 | 8.78 ± 0.91 | NR | 8Ws | Family | Group | A D | NR | US |
| Dewar, 2013 | FTF-PA+DI=178 vs. NCG=179 | Obesity | 13.20 ± 0.50 | | 52.04% | 12Ms | School | Group | A, B, D | Cluster | Australia |
| Gentile, 2009 | FTF-PA+DI=670 vs. NCG=653 | Obesity | 9.60 ± 0.90 | 9.60 ± 0.90 | 47.17% | 6Ms | School | NR | A | NR | US |
| Grydeland, 2014 | FTF-PA+DI=527 vs. NCG=958 | Overweight and obesity | 11.20 ± 0.30 | 11.20 ± 0.30 | 51.44% | 20Ms | School | NR | A, B, C | Cluster | Norway |
| Mourad, 2013 | FTF-PA+DI=193 vs. NCG=181 | Obesity | 10.30 ± 0.96 | 10 .10± 1.00 | 54.55% | 3Ms | School | NR | A, C | Cluster | Lebanon |
| Johnston, 2013 | FTF-PA+DI=186 vs. NCG=135 | Overweight and obesity | 7.80 ± 0.40 | 7.70 ± 0.40 | 55.14% | 24Ms | School | NR | A, B | NR | US |
| Klesges, 2010 | FTF-PA+DI=153 vs. NCG=150 | Obesity | 9.30 ± 0.90 | 9.30 ± 0.90 | 0.00% | 2Ys | NR | NR | A, C, D | NR | US |
| Llargues, 2011 | FTF-PA+DI=364 vs. NCG=329 | Overweight and obesity | NR | | 54.03% | 2Ys | School | NR | A | Cluster | Spain |
| Llargués, 2012 | FTF-PA+DI=225 vs. NCG=201 | Overweight and obesity | 6.03 ± 0.30 | | 47.89% | 2Ys | School | NR | A | Parallel | Spain |
| Hrafnkelsson, 2014 | FTF-PA+DI=151 vs. NCG=170 | NR | 7.30 ± 0.30 | 7.30 ± 0.30 | 42.86% | 2Ys | School | NR | D | Cluster | Iceland |
| Magnusson, 2012 | FTF-PA+DI=151 vs. NCG=170 | NR | 7.40 ± 0.30 | 7.30 ± 0.30 | 42.86% | 2Ys | School | NR | A, C, D | Cluster | Iceland |
| Story, 2003 | FTF-PA+DI=26 vs. NCG=28 | Obesity | 9.30 ± 0.90 | 9.40 ± 0.90 | 0.00% | 12Ws | NR | Group | A, C | Parallel | US |
| Heer, 2011 | FTF-PA+DI=292 vs. NCG=354 | Overwight | 9.10 ± 1.08 | 9.27 ± 0.84 | 54.93% | 12Ws | School | NR | A | NR | US |
| Safdie, 2013 | FTF-PA+DI=262 vs. NCG=354 | Overweight and obesity | 9.70 ± 0.70 | 9.80 ± 0.80 | 50.00% | 7Ms | School | NR | A | Cluster | Mexico |
| Waling, 2010 | FTF-PA+DI=43 vs. NCG=40 | Overweight and obesity | 10.40 ± 1.09 | 10.50 ± 1.06 | 49.32% | 1Y | Family | Group | A | NR | Sweden |
| Pbert, 2016 | FTF-PA+DI=58 vs. NCG=68 | Overweight and obesity | 16.50 ± 1.23 | 16.30 ± 1.20 | 37.84% | 8Ms | School | Group | A, B, C, D | Cluster | US |
| Li, 2019 | FTF-PA+DI=826 vs. NCG=804 | Obesity | 6.15 ± 0.36 | 6.15 ± 0.35 | 54.48% | 12Ms | NR | NR | B, C, D | Cluster | China |
| Larsen, 2016 | FTF-PA+DI=55 vs. NCG=51 | Overweight and obesity | 12.0 ± 0.40 | 12.0 ± 0.40 | 44.34% | 6Ws | NR | Group | A, B, D | Parallel | Denmark |
| Kim, 2016 | FTF-PA+DI=23 vs. NCG=19 | Overweight or obesity | 9.70 ± 1.49 | 9.79± 1.62 | 57.14% | 5Ws | NR | NR | A | NR | South Korea |
| Siegrist, 2013 | FTF-PA+DI=486 vs. NCG=340 | Overwight | 8.40 ± 0.70 | | NR | 1Y | School | NR | A, B, C | NR | Germany |
| Jansen, 2011* | FTF-PA+DI=657 vs. NCG=729 | Overweight and obesity | 7.70 ± 1.00 | 7.80 ± 1.00 | 49.21% | 8Ms | School | NR | A, C | Cluster | Netherlands |
| Jansen, 2011* | FTF-PA+DI=583 vs. NCG=653 | Overweight and obesity | 10.80 ± 1.00 | 10.80 ± 1.00 | 49.19% | 8Ms | School | NR | A, C | Cluster | Netherlands |
| Kain, 2014* | FTF-PA+DI=346 vs. NCG=423 | Obesity | 6.50 ± 1.10 | 6.70 ± 1.10 | 100.00% | 12Ms | School | NR | A | Cluster | Chile |
| Kain, 2014* | FTF-PA+DI=287 vs. NCG=400 | Obesity | 6.50 ± 1.00 | 6.70 ± 1.00 | 0.00% | 12Ms | School | NR | A | Cluster | Chile |
| Santos, 2014* | FTF-PA+DI=158 vs. NCG=156 | Overweight and obesity | 9.30 ± 0.10 | 8.80 ± 0.10 | 47.76% | 1Y | School | NR | B, C | Cluster | Canada |
| Santos, 2014* | FTF-PA+DI=182 vs. NCG=151 | Overweight and obesity | 9.30 ± 0.10 | 8.80 ± 0.10 |  | 1Y | School | NR | B, C | Cluster | Canada |
| Smith, 2014 | MH-delivered PA=181 vs. NCG=180 | Obesity | 12.70 ± 0.50 | 12.70 ± 0.50 | 100.00% | 20Ws | School | NR | A, C, D | Cluster | Australia |
| Paineau, 2008 | MH-delivered DI=298 vs. NCG=418 | Obesity | 7.80 ± 0.60 | 7.60 ± 0.60 | 47.21% | 10Ms | Family | NR | A, B, C | Parallel | France |
| Ezendam, 2012 | MH-delivered PA+DI=485 vs. NCG=398 | Overweight or obesity | 12.70 ± 0.70 | 12.60 ± 0.60 | 54.77% | 4Ms | School | Group | A, C | Cluster | Netherlands |
| Lubans, 2014 | MH-delivered PA+DI=181 vs. NCG=180 | Obesity | 12.70 ± 0.50 | 12.70 ± 0.50 | 100.00% | 8Ms | School | NR | A, B, C | Cluster | Australia |
| Nollen, 2014 | MH-delivered PA+DI=26 vs. NCG=25 | Obesity | 11.30 ± 1.50 | 11.30 ± 1.70 | 0.00% | 12Ws | NR | NR | A | NR | US |
| Morgan, 2011 | FTF-MLI=26 vs. NCG=26 | Overwight | 8.40 ± 2.10 | 7.90 ± 1.90 | 53.52% | 3Ms | NR | NR | A, C | NR | Australia |
| Robinson, 2003 | FTF-MLI=28 vs. NCG=33 | Obesity | 9.50 ± 0.80 | 9.50 ± 0.90 | 0.00% | 12Ws | NR | Group | A, C | Parallel | US |
| Morgan, 2012 | FTF-MLI=50 vs. NCG=50 | Obesity | 14.40 ± 0.70 | 14.20 ± 0.40 | 100.00% | 6Ms | School | NR | A, D | NR | Australia |
| Lubans, 2012 | FTF-MLI=50 vs. NCG=50 | Obesity | 14.40 ± 0.70 | | 100.00% | 6Ms | School | NR | A | NR | Australia |
| Kalarchian, 2009 | FTF-MLI=97 vs. NCG=95 | Overweight and obesity | 10.07 ± 1.19 | 10.30 ± 1.21 | 43.23% | 12Ms | Family | Group | A, B | NR | US |
| Wafa, 2011 | FTF-MLI=52 vs. NCG=55 | Obesity | 9.70 ± 1.40 | 9.90 ± 1.60 | 50.47% | 26Ws | Family | Group | B | NR | Malaysia |
| Kalavainen, 2007 | FTF-MLI=35 vs. NCG=35 | Obesity | 8.10 ± 0.90 | 8.00 ± 0.80 | 40.00% | 6Ms | School | Group | A, B | NR | Finland |
| Sacher, 2010 | FTF-MLI=60 vs. NCG=56 | Obesity | 10.30 ± 1.30 | 10.20 ± 1.30 | 45.69% | 6Ms | NR | NR | A, B, C, D | NR | UK |
| Janicke, 2008 | FTF-MLI=33 vs. NCG=26 | Overweight or obesity | NR | | 31.11% | 4Ms | Family | Group | B | NR | US |
| Nemet, 2005 | FTF-MLI=24 vs. NCG=22 | Obesity | 10.90 ± 1.90 | 11.30 ± 2.80 | 56.52% | 3Ms | NR | NR | A, D | NR | Israel |
| Savoye, 2007 | FTF-MLI=105 vs. NCG=69 | Overwight | 12.00 ± 2.40 | 12.20 ± 2.10 | 41.53% | 6Ms | NR | Group | A, B | Parallel | US |
| Shaw, 2009 | FTF-MLI=13 vs. NCG=10 | Obesity | NR | | 39.13% | 12Ms | NR | NR | A, B, C, D | NR | US |
| Hughes, 2008 | FTF-MLI=69 vs. NCG=65 | Overweight and obesity | 9.10 ± 1.70 | 8.50 ± 1.90 | 44.03% | 6Ms | NR | Individual | A, B, D | NR | UK |
| Balagopal, 2003 | FTF-MLI=7 vs. NCG=8 | Obesity | 15.60 ± 0.30 | 15.90 ± 0.50 | NR | 3Ms | NR | NR | A, D | NR | US |
| Jiang, 2005 | FTF-MLI=36 vs. NCG=39 | Obesity | 13.30 ± 0.60 | 13.20 ± 0.70 | 61.19% | 2Ys | Family | Individual | A | NR | China |
| McCallum, 2007 | FTF-MLI=82 vs. NCG=81 | Overweight or obesity | 7.50 ± 1.60 | 7.40 ± 1.60 | 48.47% | 9Ms | NR | Individual | A | NR | Australia |
| Shelton, 2007 | FTF-MLI=28 vs. NCG=15 | Overwight | 7.89 ± 1.77 | 7.33± 2.16 | 46.51% | 3Ms | NR | NR | A | NR | Australia |
| Tsiros, 2008 | FTF-MLI=25 vs. NCG=22 | Overweight and obesity | 14.50 ± 1.60 | | 34.04% | 10Ws | NR | NR | A, C, D | NR | Australia |
| Kitzman-Ulrich, 2009 | FTF-MLI=14 vs. NCG=8 | Overwight | NR | | NR | 16Ws | Family | NR | B | NR | US |
| Wake, 2009 | FTF-MLI=139 vs. NCG=119 | Overweight or obesity | 7.40 ± 1.40 | 7.60 ± 1.40 | 39.53% | 6Ms | Family | NR | A | NR | Australia |
| Reinehr, 2010 | FTF-MLI=39 vs. NCG=32 | Overwight | 11.60 ± 1.60 | 11.40 ± 1.70 | 39.39% | 6Ms | Family | NR | A, C, D | NR | Germany |
| Boudreau, 2013 | FTF-MLI=23 vs. NCG=18 | Obesity | 10.20± 1.30 | 10.40 ± 1.20 | 38.46% | 6Ms | Family | NR | B | NR | US |
| Backlund, 2011 | FTF-MLI=46 vs. NCG=44 | overweight and obesity | NR | | NR | 2Ys | Family | Individual | A | NR | Sweden |
| Boodai, 2014 | FTF-MLI=41 vs. NCG=41 | Obesity | 12.40 ± 1.20 | 12.40 ± 1.20 | 51.22% | 6Ms | NR | NR | B | NR | Kuwait |
| Croker, 2012 | FTF-MLI=37 vs. NCG=35 | Obesity | 10.80 ± 1.60 | 9.80 ± 1.40 | 30.56% | 6Ms | NR | Group | A, C | Parallel | UK |
| Davoli, 2013 | FTF-MLI=187 vs. NCG=185 | Overwight | 6.60 ± 1.02 | 6.50 ± 115 | 38.44% | 12Ms | Family | Group | A, B | Parallel | Italy |
| DeBar, 2012 | FTF-MLI=105 vs. NCG=103 | Overwight | 14.12 ± 1.48 | 14.03 ± 1.50 | NR | 6Ms | NR | NR | B | NR | US |
| Díaz, 2010 | FTF-MLI=21 vs. NCG=20 | Obesity | 11.60 ± 2.10 | 11.70 ± 2.20 | 48.84% | 12Ms | NR | Group | A, B, C | NR | Mexico |
| Ford, 2009 | FTF-MLI=54 vs. NCG=52 | Obesity | 12.70 ± 2.20 | 12.50 ± 2.30 | 44.34% | 12Ms | NR | Individual | D | NR | Australia |
| Gong, 2014 | FTF-MLI=160 vs. NCG=166 | Overweight and obesity | 8.80 ± 1.30 | 8.90 ± 1.30 | 65.03% | 1Ys | NR | NR | A, B, C, D | Parallel | China |
| Gourlan, 2013 | FTF-MLI=28 vs. NCG=34 | Obesity | NR | | NR | 6Ms | NR | NR | A | NR | Franch |
| Grey, 2009 | FTF-MLI=112 vs. NCG=86 | NR | 12.80 ± 0.70 | 12.60 ± 0.70 | 46.46% | 12Ms | School | NR | A, C, D | NR | US |
| Hofsteenge, 2014 | FTF-MLI=71 vs. NCG=51 | Overweight and obesity | 14.50 ± 1.70 | 14.40 ± 1.80 | 44.26% | 6Ms | NR | Group | A, B, C, D | NR | Netherlands |
| Johnston, 2007 | FTF-MLI=46 vs. NCG=25 | overwight | 12.20 ± 0.80 | 8.90 ± 1.30 | 45.07% | 3Ms | School | Individual | A, D | NR | US |
| Kalavainen, 2012 | FTF-MLI=35 vs. NCG=35 | Obesity | 8.80 ± 1.30 | 12.20 ± 0.70 | 20.97% | 6Ms | NR | Group | C | NR | China |
| Maddison, 2014 | FTF-MLI=127 vs. NCG=124 | Overweight and obesity | NR | | 56.57% | 24Ws | Family | NR | A, B, C, D | Parallel | New Zealand |
| Nguyen, 2012 | FTF-MLI=73 vs. NCG=78 | overwight | 14.00 ± 0.90 | 14.20 ± 1.00 | 48.34% | 12Ms | NR | NR | A, B, C | NR | Australia |
| Pakpour, 2015 | FTF-MLI=119 vs. NCG=119 | Obesity | 15.57 ± 1.38 | 15.78 ± 1.19 | 50.42% | 12Ms | School | NR | A, B, C, D | NR | Iran |
| Pbert, 2013 | FTF-MLI=42 vs. NCG=40 | Overweight and obesity | 15.90 ± 1.03 | 15.70 ± 1.01 | 30.49% | 2Ms | School | NR | A, B, C, D | Culster | UK |
| Savoye, 2011 | FTF-MLI=105 vs. NCG=69 | Obesity | 12.00 ± 2.50 | 12.50 ± 2.30 | 39.66% | 12Ms | NR | Group | A, B, D | Parallel | US |
| Savoye, 2014 | FTF-MLI=38 vs. NCG=37 | Obesity | 12.90 ± 2.00 | 12.90 ± 1.50 | 32.76% | 6Ms | NR | Group | A, B, D | Parallel | US |
| Vos, 2011 | FTF-MLI=40 vs. NCG=39 | Obesity | 13.30 ± 2.00 | 13.10 ± 1.90 | 46.84% | 3Ms | NR | Group | B | NR | Netherlands |
| Wright, 2014 | FTF-MLI=118 vs. NCG=123 | Obesity | NR | | NR | 4Ms | NR | Group | A, B, C | Parallel | US |
| Nayak, 2016 | FTF-MLI=120 vs. NCG=131 | Obesity | NR | | 51.39% | 6Ms | School | Group | A | NR | India |
| Morell-Azanza, 2019 | FTF-MLI=89 vs. NCG=32 | Abdominal obesity | 11.50 ± 2.48 | 10.74 ± 2.39 | 38.46% | 8Ws | Family | NR | A, B, C | NR | Spain |
| Lloyd, 2017 | FTF-MLI=676 vs. NCG=648 | Obesity | 9.30 ± 0.30 | 9.70 ± 0.30 | 48.72% | 18Ms | School | Group | B | Cluster | UK |
| Liu, 2018 | FTF-MLI=930 vs. NCG=959 | Obesity | 9.15 ± 0.75 | 9.06 ± 0.58 | 51.72% | 1Ys | School | Group | A, B | Cluster | China |
| Ahmad, 2018 | FTF-MLI=67 vs. NCG=67 | Overweight or obesity | 9.60 ± 1.20 | 9.60 ± 1.20 | NR | 4Ws | Family | NR | B, C, D | Parallel | Malaysia |
| Yusop, 2018 | FTF-MLI=25 vs. NCG=25 | Obesity | NR | | 52.50% | 6Ms | NR | NR | C, D | NR | Malaysia |
| Singh, 2009* | FTF-MLI=295 vs. NCG=222 | Overweight and obesity | 12.80 ± 0.50 | 12.90 ± 0.50 | 46.66% | 8Ms | NR | NR | A, C | NR | Netherlands |
| Singh, 2009* | FTF-MLI=337 vs. NCG=254 | Overweight and obesity | 12.60 ± 0.50 | 12.70 ± 0.50 |  | 8Ms | NR | NR | A, C | NR | Netherlands |
| Saelens, 2002 | MH-delivered MLI=23 vs. NCG=21 | Overweight or obesity | 14.20 ± 1.20 | | 59.09% | 4Ms | NR | Individual | A | NR | US |
| Lubans, 2012 | MH-delivered MLI=178 vs. NCG=179 | Obesity | 13.15 ± 0.44 | 13.20 ± 0.45 | 0.00% | 12Ms | School | Group | A, B, D | Cluster | Australia |
| Niet, 2012 | MH-delivered MLI=73 vs. NCG=68 | Overweight and obesity | 10.00 ± 1.30 | 9.80 ± 1.30 | 33.77% | 9Ms | NR | Individual | B | Parallel | Netherlands |
| Davis, 2013 | MH-delivered MLI=31 vs. NCG=27 | Obesity | 8.48 ± 1.73 | 8.69 ± 1.78 | 70.69% | 8Ws | Family | NR | B | NR | US |
| Williamson, 2005 | MH-delivered MLI=28 vs. NCG=29 | Overwight | 13.14 ± 1.59 | 13.23 ± 1.16 | NR | 6Ms | NR | NR | A, D | NR | US |
| Chen, 2019 | MH-delivered MLI=23 vs. NCG=17 | Overweight or obesity | 14.90 ± 1.67 | | 57.50% | 3Ms | NR | NR | A | NR | US |
| Bruñól, 2018 | MH-delivered PA=18 vs. FTF-PA=16 | Overweight or obesity | 12.10 ± 1.40 | 12.60 ± 1.60 | 44.00% | 5Ws | Family | Individual | D | NR | Spain |
| Saelens, 2011 | PA+DI=15 vs. DI=14 | Overwight | 10.10 ± 1.10 | 9.80 ± 1.20 | 44.83% | 15Ws | School | Group | A, B, C, D | Parallel | US |
| Prado, 2010 | FTF-PA+DI=18 vs. FTF-DI=15 | Obesity | 10.30 ± 0.20 | 10.20 ± 0.30 | NR | 4Ms | NR | NR | A, B, D | NR | US |
| Xu, 2017* | FTF-PA+DI=3773 vs. NCG=3944 | Overweight and obesity | NR | | 50.72% | 1Y | School | Group | A, B, C, D | Cluster | China |
| Xu, 2017* | FTF-PA=778 vs. FTF-DI=792 vs. NCG=580 | Overweight and obesity | NR | | 55.12% | 1Y | School | Group | A, B, C, D | Cluster | China |
| DAVIS, 2009 | FTF-DI=10 vs. FTF-PA+DI=9 vs. NCG=7 | Adiposity | FTF-DI:15.30 ± 1.10 FTF-PA+DI:15.7 ± 1.20 NCG:15.10 ± 1.10 | | 0.00% | 16Ws | NR | Individual | A | NR | US |
| Meng, 2013 | FTF-PA=590 vs. FTF-DI=615, FTF-PA+DI=3358 vs. NCG=460 | Overweight and obesity | NR | | 54.10% | 1Y | School | NR | A, B | Cluster | China |
| SEVİNÇ, 2011 | FTF-DI=1989 vs. PA+DI=1932 vs. NCG=2926 | Obesity | NR | | 50.40% | 8Ms | School | NR | A | NR | Turkey |
| Hao, 2019* | FTF-PA=25 vs. FTF-DI=27 vs. FTF-PA+DI=25 vs. NCG=27 | Overwight | NR | | 0.00% | 2Ms | School | Group | A | NR | China |
| Hao, 2019* | FTF-PA=32 vs. FTF-DI=33 vs. FTF-PA+DI=31 vs. NCG=29 | Overwight | NR | | 100.00% | 2Ms | School | Group | A | NR | China |

A, BMI; B, BMI Z-score; C, Waist circumference; D, Percent body fat;*, Studies were split based on the gender, grade and other subgroup.

DI, Dietary intervention; FTF, Face-to-face; IG, Intervention group; MH-delivered, Mobile health-delivered; MLI, Muti-lifestyle intervention; Ms, Months; NCG, Named control group; NR, Not reported; PA, Physical activity; Ws, Weeks; Ys, Years.

**Table. S2(A) Relative effect sizes of treatments efficacy at post-treatment based on network meta-analysis(WC outcome).**

**Table. S2(B) Relative effect sizes of treatments efficacy at post-treatment based on network meta-analysis(PBF outcome).**

Treatments are orders in the rank of their chance of being the best treatment. Numbers in black boxes are SUCRA(the surface under the cumulative ranking curve) values and their CrIs(credible intervals), which represented the rank of the treatment. Significant pairwise comparisons are highlighted in grey and in bold. For efficacy in post-treatment, standardized mean differences(MDs) less than 0 favor the column-defining treatment. DI, Dietary intervention; FTF, Face-to-face; MH-delivered, Mobile health-based; MLI, Muti-lifestyle intervention; NCG, Named control group; PA, Physical activity.

**Table. S3(A) Subgroup analyses of grade for varied treatments based on the outcome of BMI.**

Numbers in light blue boxes are MD values and their CrIs, which represented the efficacy of varied treatments based on the Grade of children. On the contrary, numbers in green

boxes are relative effects based on the Grade of adolescents. All significant pairwise comparisons are in bold. For efficacy of Grade of children, MDs less than 0 favor the column-defining treatment. For efficacy of Grade of adolescents, MD lower than 0 favor the row-defining treatment. DI, Dietary intervention; FTF, Face-to-face; MH-delivered, Mobile health-based; MLI, Muti-lifestyle intervention; NCG, Named control group; PA, Physical activity.

**Table. S3(B) Subgroup analyses of region for varied treatments based on the outcome of BMI.**

Numbers in light blue boxes are MD values and their CrIs, which represented the efficacy of varied treatments based on the Developed countries. On the contrary, numbers in green

boxes are relative effects based on the Developing or undeveloped countries. All significant pairwise comparisons are in bold. For efficacy of Developed countries, MDs less than 0 favor the column-defining treatment. For efficacy of Developing or undeveloped countries, MD lower than 0 favor the row-defining treatment. DI, Dietary intervention; FTF, Face-to-face; MH-delivered, Mobile health-based; MLI, Muti-lifestyle intervention; NCG, Named control group; PA, Physical activity.

**Table. S4(A) Subgroup analyses of publication year for varied treatments based on the outcome of BMI.**

Numbers in light blue boxes are MD values and their CrIs, which represented the efficacy of varied treatments based on the Publication year equal or greater than 2010. On the contrary, numbers in green boxes are relative effects based on the Publication year less than 2010. All significant pairwise comparisons are in bold. For efficacy of Publication year equal or greater than 2010, MDs less than 0 favor the column-defining treatment. For efficacy of Publication year less than 2010, MD lower than 0 favor the row-defining treatment. DI, Dietary intervention; FTF, Face-to-face; MH-delivered, Mobile health-based; MLI, Muti-lifestyle intervention; NCG, Named control group; PA, Physical activity.

**Table. S4(B) Subgroup analyses of total sample size for varied treatments based on the outcome of BMI.**

Numbers in light blue boxes are MD values and their CrIs, which represented the efficacy of varied treatments based on the Total sample size equal or greater than 100. On the contrary, numbers in green boxes are relative effects based on the Total sample size less than 100. All significant pairwise comparisons are in bold. For efficacy of Total sample size equal or greater than 100, MDs less than 0 favor the column-defining treatment. For efficacy of Total sample size less than 100, MD lower than 0 favor the row-defining treatment. DI, Dietary intervention; FTF, Face-to-face; MH-delivered, Mobile health-based; MLI, Muti-lifestyle intervention; NCG, Named control group; PA, Physical activity.

**Table. S5(A) Subgroup analyses of gender ratio for varied treatments based on the outcome of BMI.**

Numbers in light blue boxes are MD values and their CrIs, which represented the efficacy of varied treatments based on the Gender ratio equal or greater than 1. On the contrary, numbers in green boxes are relative effects based on the Gender ratio less than 1. All significant pairwise comparisons are in bold. For efficacy of Gender ratio equal or greater than 1, MDs less than 0 favor the column-defining treatment. For efficacy of Gender ratio less than 1, MD lower than 0 favor the row-defining treatment. DI, Dietary intervention; FTF, Face-to-face; MH-delivered, Mobile health-based; MLI, Muti-lifestyle intervention; NCG, Named control group; PA, Physical activity.

**Table. S5(B) Subgroup analyses of intervention context for varied treatments based on the outcome of BMI.**

Numbers in light blue boxes are MD values and their CrIs, which represented the efficacy of varied treatments were conducted based on the School. On the contrary, numbers in green boxes are relative effects based on the Family. All significant pairwise comparisons are in bold. For efficacy of treatment conducted in School, MDs less than 0 favor the column-defining treatment. For efficacy of treatment conducted with Family, MD lower than 0 favor the row-defining treatment. DI, Dietary intervention; FTF, Face-to-face; MH-delivered, Mobile health-based; MLI, Muti-lifestyle intervention; NCG, Named control group; PA, Physical activity.

**Table. S6(A) Subgroup analyses of setting for varied treatments based on the outcome of BMI.**

Numbers in light blue boxes are MD values and their CrIs, which represented the efficacy of varied treatments based on the Group setting. On the contrary, numbers in green boxes are relative effects based on the Individual setting. All significant pairwise comparisons are in bold. For efficacy of the Group setting, MDs less than 0 favor the column-defining treatment. For efficacy of the Individual setting, MD lower than 0 favor the row-defining treatment. DI, Dietary intervention; FTF, Face-to-face; MH-delivered, Mobile health-based; MLI, Muti-lifestyle intervention; NCG, Named control group; PA, Physical activity.

**Table. S6(B) Subgroup analyses of treatment duration for varied treatments based on the outcome of BMI.**

Numbers in light blue boxes are MD values and their CrIs, which represented the efficacy of varied treatments were conducted based on the duration greater than or equal to 12months. On the contrary, numbers in green boxes are relative effects the treatment duration less than 12months. All significant pairwise comparisons are in bold. For efficacy of treatment duration greater than or equal to 12months, MDs less than 0 favor the column-defining treatment. For efficacy of treatment duration less than 12months, MD lower than 0 favor the row-defining treatment. DI, Dietary intervention; FTF, Face-to-face; MH-delivered, Mobile health-based; MLI, Muti-lifestyle intervention; NCG, Named control group; PA, Physical activity.

**Table. S7(A) Sensitivity analyses of outcome assessment of interventions duration for varied treatments based on the outcome of BMI.**

Numbers in light blue boxes are MD values and their CrIs, which represented the efficacy of varied treatments based on the intervention duration above or equal 12 months. On the contrary, numbers in green boxes are relative effects based on the above or equal 24 months. All significant pairwise comparisons are in bold. For efficacy of Developed countries, MDs less than 0 favor the column-defining treatment. For efficacy of Developing or undeveloped countries, MD lower than 0 favor the row-defining treatment. DI, Dietary intervention; FTF, Face-to-face; MH-delivered, Mobile health-based; MLI, Muti-lifestyle intervention; NCG, Named control group; PA, Physical activity.

**Table. S7(B) Sensitivity analyses of outcome assessment of interventions duration for varied treatments based on the outcome of BMI Z-score.**

Numbers in light blue boxes are MD values and their CrIs, which represented the efficacy of varied treatments based on the intervention duration above or equal 12 months. On the contrary, numbers in green boxes are relative effects based on the above or equal 24 months. All significant pairwise comparisons are in bold. For efficacy of Developed countries, MDs less than 0 favor the column-defining treatment. For efficacy of Developing or undeveloped countries, MD lower than 0 favor the row-defining treatment. DI, Dietary intervention; FTF, Face-to-face; MH-delivered, Mobile health-based; MLI, Muti-lifestyle intervention; NCG, Named control group; PA, Physical activity.

**Table. S8(A) Sensitivity analyses of outcome assessment of interventions duration for varied treatments based on the outcome of BMI Z-score.**

Numbers in light blue boxes are MD values and their CrIs, which represented the efficacy of varied treatments based on the intervention duration above or equal 12 months. On the contrary, numbers in green boxes are relative effects based on the above or equal 24 months. All significant pairwise comparisons are in bold. For efficacy of Developed countries, MDs less than 0 favor the column-defining treatment. For efficacy of Developing or undeveloped countries, MD lower than 0 favor the row-defining treatment. DI, Dietary intervention; FTF, Face-to-face; MH-delivered, Mobile health-based; MLI, Muti-lifestyle intervention; NCG, Named control group; PA, Physical activity.

**Table. S8(B) Sensitivity analyses of outcome assessment of interventions duration for varied treatments based on the outcome of BMI Z-score.**

Numbers in light blue boxes are MD values and their CrIs, which represented the efficacy of varied treatments based on the intervention duration above or equal 12 months. On the contrary, numbers in green boxes are relative effects based on the above or equal 24 months. All significant pairwise comparisons are in bold. For efficacy of Developed countries, MDs less than 0 favor the column-defining treatment. For efficacy of Developing or undeveloped countries, MD lower than 0 favor the row-defining treatment. DI, Dietary intervention; FTF, Face-to-face; MH-delivered, Mobile health-based; MLI, Muti-lifestyle intervention; NCG, Named control group; PA, Physical activity.

**Table. S9 The confidence in MD for mean overall change in BMI by GRADE system.**

| **Comparison** | **Downgrade elements** | | | | | **Upgrade elements** | **GRADE** |
| --- | --- | --- | --- | --- | --- | --- | --- |
|  | **Study limitation** | **Imprecision** | **Inconsistency** | **Indirectness** | **Publication bias** |  |  |
| FTF-PA+DI vs. NCG | Downgrade(-) | No downgrade | No downgrade | Downgrade(-) | Downgrade(-) | Upgrade(+) | Low(++) |
| FTF-MLI vs. NCG | Downgrade(-) | No downgrade | No downgrade | Downgrade(-) | Downgrade(-) | Upgrade(+) | Low(++) |
| MH-delivered MLI vs. NCG | No downgrade | Downgrade(-) | No downgrade | Downgrade(-) | No downgrade | Upgrade(+) | Moderate(+++) |
| MH-delivered PA vs. NCG | No downgrade | Downgrade(-) | No downgrade | No downgrade | No downgrade | No upgrade | Moderate(+++) |
| MH-delivered PA+DI vs. NCG | No downgrade | Downgrade(-) | No downgrade | No downgrade | No downgrade | No upgrade | Moderate(+++) |
| FTF-PA vs. NCG | Downgrade(-) | No downgrade | No downgrade | No downgrade | Downgrade(-) | No upgrade | Low(++) |
| FTF-DI vs. NCG | Downgrade(-) | No downgrade | No downgrade | Downgrade(-) | Downgrade(-) | No upgrade | Very low(+) |
| MH-delivered DI vs. NCG | No downgrade | Downgrade(-) | No downgrade | No downgrade | No downgrade | No upgrade | Moderate(+++) |
| FTF-PA+DI vs. MH-delivered DI | No downgrade | Downgrade(-) | No downgrade | No downgrade | No downgrade | No upgrade | Moderate(+++) |
| FTF-MLI vs. MH-delivered DI | No downgrade | Downgrade(-) | No downgrade | No downgrade | No downgrade | No upgrade | Moderate(+++) |
| MH-delivered MLI vs. MH-delivered DI | No downgrade | Downgrade(-) | No downgrade | No downgrade | No downgrade | No upgrade | Moderate(+++) |
| MH-delivered PA vs. MH-delivered DI | No downgrade | Downgrade(-) | No downgrade | No downgrade | No downgrade | No upgrade | Moderate(+++) |
| MH-delivered PA+DI vs. MH-delivered DI | No downgrade | Downgrade(-) | No downgrade | No downgrade | No downgrade | No upgrade | Moderate(+++) |
| FTF-PA vs. MH-delivered DI | No downgrade | Downgrade(-) | No downgrade | No downgrade | No downgrade | No upgrade | Moderate(+++) |
| FTF-DI vs. MH-delivered DI | No downgrade | Downgrade(-) | No downgrade | No downgrade | No downgrade | No upgrade | Moderate(+++) |
| FTF-PA+DI vs. FTF-DI | Downgrade(-) | No downgrade | Downgrade(-) | Downgrade(-) | Downgrade(-) | No upgrade | Very low |
| FTF-MLI vs. FTF-DI | No downgrade | No downgrade | No downgrade | Downgrade(-) | No downgrade | No upgrade | Moderate(+++) |
| MH-delivered MLI vs. FTF-DI | No downgrade | No downgrade | No downgrade | No downgrade | No downgrade | No upgrade | High(++++) |
| MH-delivered PA vs. FTF-DI | No downgrade | Downgrade(-) | No downgrade | No downgrade | No downgrade | No upgrade | Moderate(+++) |
| MH-delivered PA+DI vs. FTF-DI | No downgrade | Downgrade(-) | No downgrade | Downgrade(-) | No downgrade | No upgrade | Low(++) |
| FTF-PA vs. FTF-DI | Downgrade(-) | No downgrade | Downgrade(-) | Downgrade(-) | No downgrade | No upgrade | Very low(+) |
| FTF-PA+DI vs. FTF-PA | Downgrade(-) | No downgrade | Downgrade(-) | Downgrade(-) | No downgrade | No upgrade | Very low(+) |
| FTF-MLI vs. FTF-PA | No downgrade | No downgrade | No downgrade | Downgrade(-) | No downgrade | No upgrade | Moderate(+++) |
| MH-delivered MLI vs. FTF-PA | No downgrade | No downgrade | No downgrade | No downgrade | No downgrade | No upgrade | High(++++) |
| MH-delivered PA vs. FTF-PA | No downgrade | Downgrade(-) | No downgrade | No downgrade | No downgrade | No upgrade | Moderate(+++) |
| MH-delivered PA+DI vs. FTF-PA | No downgrade | Downgrade(-) | No downgrade | No downgrade | No downgrade | No upgrade | Moderate(+++) |
| FTF-PA+DI vs. MH-delivered PA+DI | No downgrade | Downgrade(-) | No downgrade | No downgrade | No downgrade | No upgrade | Moderate(+++) |
| FTF-MLI vs.MH-delivered PA+DI | No downgrade | Downgrade(-) | No downgrade | No downgrade | No downgrade | No upgrade | Moderate(+++) |
| MH-delivered MLI vs. MH-delivered PA+DI | No downgrade | Downgrade(-) | No downgrade | No downgrade | No downgrade | No upgrade | Moderate(+++) |
| MH-delivered PA vs. MH-delivered PA+DI | No downgrade | Downgrade(-) | No downgrade | No downgrade | No downgrade | No upgrade | Moderate(+++) |
| FTF-PA+DI vs. MH-delivered PA | No downgrade | Downgrade(-) | No downgrade | No downgrade | No downgrade | No upgrade | Moderate(+++) |
| FTF-MLI vs. MH-delivered PA | No downgrade | Downgrade(-) | No downgrade | No downgrade | No downgrade | No upgrade | Moderate(+++) |
| MH-delivered MLI vs. MH-delivered PA | No downgrade | Downgrade(-) | No downgrade | No downgrade | No downgrade | No upgrade | Moderate(+++) |
| FTF-PA+DI vs. MH-delivered MLI | No downgrade | No downgrade | No downgrade | No downgrade | No downgrade | No upgrade | High(++++) |
| FTF-MLI vs. MH-delivered MLI | No downgrade | No downgrade | No downgrade | No downgrade | No downgrade | No upgrade | High(++++) |
| FTF-PA+DI vs. FTF-MLI | No downgrade | No downgrade | No downgrade | No downgrade | No downgrade | No upgrade | High(++++) |

Comparisons were initially rated as high-quality evidence(four plus:++++) and were downgraded accordingly, based on study limitations, imprecision, inconsistency, indirectness, and publication bias.

a, study limitation: concerns about selection bias, performance bias, detection bias, attrition bias, reporting bias or other bias;

b, imprecision: concerns about sample size was insufficient or if imprecise estimates of wide confidence interval were generated in this comparison;

c, inconsistency: concerns about the study heterogeneity, especially the local inconsistency was found between direct and indirect evidence among the comparisons;

d, indirectness: concerns about there were heterogeneity observed based on four domains, namely, differences in populations, interventions, outcome measures, indirect comparisons and the combined effect of the four types of indirectness;

e, publication bias: concerns about publication bias due to an asymmetrical funnel plot was generated.

Each item was rated as no downgrade, downgrade one level(serious) or downgrade two levels(very serious). Upgrade due to large effect(large: upgrade one level, +1; very large: upgrade two level, +2), dose response(evidence of a gradient: upgrade one level, +1), or all plausible residual confounding(would reduce a demonstrated effect: upgrade one level, +1; would suggest a spurious effect if no effect was observed: upgrade two level, +2). All comparison were rated as four levels of evidence: High, We are very confident that the true effect lies close to that of the estimate of the effect(four plus:++++); Moderate, We are moderately confident in the effect estimate: The true effect is likely to be close to the estimate of the effect, but there is a possibility that it is substantially different(three plus:+++); Low, Our confidence in the effect estimate is limited: The true effect may be substantially different from the estimate of the effect(two plus:++); Very low, We have very little confidence in the effect estimate: The true effect is likely to be substantially different from the estimate of effect(one plus:+).

Reference available from: Balshem H, Helfand M, HJ Schünemann, et al. GRADE guidelines: 3. Rating the quality of evidence[J]. Journal of clinical epidemiology, 2011, 64(4):401-406.

**Table. S10 The confidence in MD for mean overall change in BMI Z-score by GRADE system.**

| **Comparison** | **Downgrade elements** | | | | | **Upgrade elements** | **GRADE** |
| --- | --- | --- | --- | --- | --- | --- | --- |
|  | **Study limitation** | **Imprecision** | **Inconsistency** | **Indirectness** | **Publication bias** |  |  |
| FTF-PA+DI vs. NCG | Downgrade(-) | No downgrade | No downgrade | Downgrade(-) | Downgrade(-) | Upgrade(+) | Low(++) |
| MH-delivered PA+DI vs. NCG | No downgrade | No downgrade | No downgrade | Downgrade(-) | Downgrade(-) | Upgrade(+) | Moderate(+++) |
| FTF-MLI vs. NCG | Downgrade(-) | No downgrade | No downgrade | Downgrade(-) | No downgrade | No upgrade | Low(++) |
| FTF-PA vs. NCG | Downgrade(-) | No downgrade | No downgrade | No downgrade | Downgrade(-) | No upgrade | Low(++) |
| MH-delivered DI vs. NCG | No downgrade | No downgrade | No downgrade | No downgrade | Downgrade | No upgrade | High(++++) |
| MH-delivered PA vs. NCG | No downgrade | Downgrade(-) | No downgrade | No downgrade | No downgrade | No upgrade | Moderate(+++) |
| FTF-DI vs. NCG | Downgrade(-) | Downgrade(-) | No downgrade | No downgrade | No downgrade | No upgrade | Low(++) |
| FTF-PA+DI vs. FTF-DI | Downgrade(-) | No downgrade | Downgrade(-) | Downgrade(-) | No downgrade | No upgrade | Very low(+) |
| MH-delivered PA+DI vs.FTF-DI | No downgrade | No downgrade | No downgrade | No downgrade | No downgrade | No upgrade | High(++++) |
| FTF-MLI vs. FTF-DI | No downgrade | No downgrade | No downgrade | No downgrade | Downgrade(-) | No upgrade | Moderate(+++) |
| FTF-PA vs. FTF-DI | Downgrade(-) | No downgrade | Downgrade(-) | Downgrade(-) | No downgrade | No upgrade | Very low(+) |
| MH-delivered DI vs. FTF-DI | No downgrade | Downgrade(-) | No downgrade | No downgrade | No downgrade | No upgrade | Moderate(+++) |
| MH-delivered PA vs. FTF-DI | No downgrade | No downgrade | No downgrade | No downgrade | No downgrade | No upgrade | High(++++) |
| FTF-PA+DI vs. MH-delivered PA | No downgrade | No downgrade | No downgrade | No downgrade | No downgrade | No upgrade | High(++++) |
| MH-delivered PA+DI vs.MH-delivered PA | No downgrade | No downgrade | No downgrade | No downgrade | No downgrade | No upgrade | High(++++) |
| FTF-MLI vs. MH-delivered PA | No downgrade | No downgrade | No downgrade | No downgrade | No downgrade | No upgrade | High(++++) |
| FTF-PA vs. MH-delivered PA | No downgrade | No downgrade | No downgrade | No downgrade | No downgrade | No upgrade | High(++++) |
| MH-delivered DI vs. MH-delivered PA | No downgrade | Downgrade(-) | No downgrade | No downgrade | No downgrade | No upgrade | Moderate(+++) |
| FTF-PA+DI vs. MH-delivered DI | No downgrade | No downgrade | No downgrade | No downgrade | No downgrade | No upgrade | High(++++) |
| MH-delivered PA+DI vs. MH-delivered DI | No downgrade | Downgrade(-) | No downgrade | No downgrade | No downgrade | No upgrade | Moderate(+++) |
| FTF-MLI vs. MH-delivered DI | No downgrade | No downgrade | No downgrade | No downgrade | No downgrade | No upgrade | High(++++) |
| FTF-PA vs. MH-delivered DI | No downgrade | No downgrade | No downgrade | No downgrade | No downgrade | No upgrade | High(++++) |
| FTF-PA+DI vs. FTF-PA | No downgrade | No downgrade | Downgrade | Downgrade(-) | No downgrade | No upgrade | Moderate(+++) |
| MH-delivered PA+DI vs. FTF-PA | No downgrade | No downgrade | No downgrade | No downgrade | No downgrade | No upgrade | High(++++) |
| FTF-MLI vs. FTF-PA | No downgrade | No downgrade | No downgrade | No downgrade | No downgrade | No upgrade | High(++++) |
| FTF-PA+DI vs. FTF-MLI | No downgrade | No downgrade | No downgrade | No downgrade | No downgrade | No upgrade | High(++++) |
| MH-delivered PA+DI vs. FTF-MLI | No downgrade | No downgrade | No downgrade | No downgrade | No downgrade | No upgrade | High(++++) |
| FTF-PA+DI vs. MH-delivered PA+DI | No downgrade | No downgrade | No downgrade | No downgrade | No downgrades | No upgrade | High(++++) |

Comparisons were initially rated as high-quality evidence(four plus:++++) and were downgraded accordingly, based on study limitations, imprecision, inconsistency, indirectness, and publication bias.

a, study limitation: concerns about selection bias, performance bias, detection bias, attrition bias, reporting bias or other bias;

b, imprecision: concerns about sample size was insufficient or if imprecise estimates of wide confidence interval were generated in this comparison;

c, inconsistency: concerns about the study heterogeneity, especially the local inconsistency was found between direct and indirect evidence among the comparisons;

d, indirectness: concerns about there were heterogeneity observed based on four domains, namely, differences in populations, interventions, outcome measures, indirect comparisons and the combined effect of the four types of indirectness;

e, publication bias: concerns about publication bias due to an asymmetrical funnel plot was generated.

Each item was rated as no downgrade, downgrade one level(serious) or downgrade two levels(very serious). Upgrade due to large effect(large: upgrade one level, +1; very large: upgrade two level, +2), dose response(evidence of a gradient: upgrade one level, +1), or all plausible residual confounding(would reduce a demonstrated effect: upgrade one level, +1; would suggest a spurious effect if no effect was observed: upgrade two level, +2). All comparison were rated as four levels of evidence: High, We are very confident that the true effect lies close to that of the estimate of the effect(four plus:++++); Moderate, We are moderately confident in the effect estimate: The true effect is likely to be close to the estimate of the effect, but there is a possibility that it is substantially different(three plus:+++); Low, Our confidence in the effect estimate is limited: The true effect may be substantially different from the estimate of the effect(two plus:++); Very low, We have very little confidence in the effect estimate: The true effect is likely to be substantially different from the estimate of effect(one plus:+).

Reference available from: Balshem H, Helfand M, HJ Schünemann, et al. GRADE guidelines: 3. Rating the quality of evidence[J]. Journal of clinical epidemiology, 2011, 64(4):401-406.

**Table. S11 The confidence in MD for mean overall change in WC by GRADE system.**

| **Comparison** | **Downgrade elements** | | | | | **Upgrade elements** | **GRADE** |
| --- | --- | --- | --- | --- | --- | --- | --- |
|  | **Study limitation** | **Imprecision** | **Inconsistency** | **Indirectness** | **Publication bias** |  |  |
| FTF-PA+DI vs. NCG | Downgrade(-) | No downgrade | No downgrade | Downgrade(-) | No downgrade | Upgrade(+) | Moderate(+++) |
| FTF-MLI vs. NCG | Downgrade(-) | No downgrade | No downgrade | Downgrade(-) | Downgrade(-) | Upgrade(+) | Low(++) |
| MH-delivered PA+DI vs. NCG | No downgrade | Downgrade(-) | No downgrade | Downgrade(-) | No downgrade | No upgrade | Low(++) |
| MH-delivered PA vs. NCG | No downgrade | Downgrade(-) | No downgrade | No downgrade | No downgrade | No upgrade | Moderate(+++) |
| MH-delivered DI vs. NCG | No downgrade | No downgrade | No downgrade | No downgrade | No downgrade | No upgrade | High(++++) |
| FTF-DI vs. NCG | Downgrade(-) | No downgrade | No downgrade | No downgrade | No downgrade | No upgrade | Moderate(+++) |
| FTF-PA vs. NCG | Downgrade(-) | No downgrade | No downgrade | No downgrade | No downgrade | No upgrade | Moderate(+++) |
| FTF-PA+DI vs. FTF-PA | No downgrade | No downgrade | No downgrade | Downgrade(-) | No downgrade | Upgrade(+) | High(++++) |
| FTF-MLI vs. FTF-PA | No downgrade | No downgrade | No downgrade | No downgrade | No downgrade | No upgrade | High(++++) |
| MH-delivered PA+DI vs. FTF-PA | No downgrade | Downgrade(-) | No downgrade | No downgrade | No downgrade | No upgrade | Moderate(+++) |
| MH-delivered PA vs. FTF-PA | No downgrade | Downgrade(-) | No downgrade | No downgrade | No downgrade | No upgrade | Moderate(+++) |
| MH-delivered DI vs. FTF-PA | No downgrade | Downgrade(-) | No downgrade | No downgrade | No downgrade | No upgrade | Moderate(+++) |
| FTF-DI vs. FTF-PA | No downgrade | Downgrade(-) | Downgrade(-) | Downgrade(-) | No downgrade | No upgrade | Very low(+) |
| FTF-PA+DI vs. FTF-DI | No downgrade | Downgrade(-) | Downgrade(-) | Downgrade(-) | No downgrade | No upgrade | Very low(+) |
| FTF-MLI vs. FTF-DI | No downgrade | Downgrade(-) | No downgrade | No downgrade | No downgrade | No upgrade | Moderate(+++) |
| MH-delivered PA+DI vs. FTF-DI | No downgrade | Downgrade(-) | No downgrade | No downgrade | No downgrade | No upgrade | Moderate(+++) |
| MH-delivered PA vs. FTF-DI | No downgrade | Downgrade(-) | No downgrade | No downgrade | No downgrade | No upgrade | Moderate(+++) |
| MH-delivered DI vs. FTF-DI | No downgrade | Downgrade(-) | No downgrade | No downgrade | No downgrade | No upgrade | Moderate(+++) |
| FTF-PA+DI vs. MH-delivered DI | No downgrade | Downgrade(-) | No downgrade | No downgrade | No downgrade | No upgrade | Moderate(+++) |
| FTF-MLI vs. MH-delivered DI | No downgrade | No downgrade | No downgrade | No downgrade | No downgrade | No upgrade | High(++++) |
| MH-delivered PA+DI vs. MH-delivered DI | No downgrade | Downgrade(-) | No downgrade | No downgrade | No downgrade | No upgrade | Moderate(+++) |
| MH-delivered PA vs. MH-delivered DI | No downgrade | Downgrade(-) | No downgrade | No downgrade | No downgrade | No upgrade | Moderate(+++) |
| FTF-PA+DI vs. MH-delivered PA | No downgrade | Downgrade(-) | No downgrade | No downgrade | No downgrade | No upgrade | Moderate(+++) |
| FTF-MLI vs. MH-delivered PA | No downgrade | Downgrade(-) | No downgrade | No downgrade | No downgrade | No upgrade | Moderate(+++) |
| MH-delivered PA+DI vs. MH-delivered PA | No downgrade | Downgrade(-) | No downgrade | No downgrade | No downgrade | No upgrade | Moderate(+++) |
| FTF-PA+DI vs. MH-delivered PA+DI | No downgrade | Downgrade(-) | No downgrade | No downgrade | No downgrade | No upgrade | Moderate(+++) |
| FTF-MLI vs. MH-delivered PA+DI | No downgrade | Downgrade(-) | No downgrade | No downgrade | No downgrade | No upgrade | Moderate(+++) |
| FTF-PA+DI vs. FTF-MLI | No downgrade | No downgrade | No downgrade | No downgrade | No downgrade | No upgrade | High(++++) |

Comparisons were initially rated as high-quality evidence(four plus:++++) and were downgraded accordingly, based on study limitations, imprecision, inconsistency, indirectness, and publication bias.

a, study limitation: concerns about selection bias, performance bias, detection bias, attrition bias, reporting bias or other bias;

b, imprecision: concerns about sample size was insufficient or if imprecise estimates of wide confidence interval were generated in this comparison;

c, inconsistency: concerns about the study heterogeneity, especially the local inconsistency was found between direct and indirect evidence among the comparisons;

d, indirectness: concerns about there were heterogeneity observed based on four domains, namely, differences in populations, interventions, outcome measures, indirect comparisons and the combined effect of the four types of indirectness;

e, publication bias: concerns about publication bias due to an asymmetrical funnel plot was generated.

Each item was rated as no downgrade, downgrade one level(serious) or downgrade two levels(very serious). Upgrade due to large effect(large: upgrade one level, +1; very large: upgrade two level, +2), dose response(evidence of a gradient: upgrade one level, +1), or all plausible residual confounding(would reduce a demonstrated effect: upgrade one level, +1; would suggest a spurious effect if no effect was observed: upgrade two level, +2). All comparison were rated as four levels of evidence: High, We are very confident that the true effect lies close to that of the estimate of the effect(four plus:++++); Moderate, We are moderately confident in the effect estimate: The true effect is likely to be close to the estimate of the effect, but there is a possibility that it is substantially different(three plus:+++); Low, Our confidence in the effect estimate is limited: The true effect may be substantially different from the estimate of the effect(two plus:++); Very low, We have very little confidence in the effect estimate: The true effect is likely to be substantially different from the estimate of effect(one plus:+).

Reference available from: Balshem H, Helfand M, HJ Schünemann, et al. GRADE guidelines: 3. Rating the quality of evidence[J]. Journal of clinical epidemiology, 2011, 64(4):401-406.

**Table. S12 The confidence in MD for mean overall change in PBF by GRADE system.**

| **Comparison** | **Downgrade elements** | | | | | **Upgrade elements** | **GRADE** |
| --- | --- | --- | --- | --- | --- | --- | --- |
|  | **Study limitation** | **Imprecision** | **Inconsistency** | **Indirectness** | **Publication bias** |  |  |
| FTF-MLI vs. NCG | Downgrade(-) | Downgrade(-) | No downgrade | Downgrade(-) | No downgrade | Upgrade(+) | Low(++) |
| FTF-PA+DI vs. NCG | Downgrade(-) | Downgrade(-) | No downgrade | Downgrade(-) | Downgrade(-) | No upgrade | Very low |
| MH-delivered PA+DI vs. NCG | No downgrade | No downgrade | No downgrade | Downgrade(-) | No downgrade | No upgrade | Moderate(+++) |
| FTF-PA vs. NCG | Downgrade(-) | No downgrade | No downgrade | No downgrade | No downgrade | No upgrade | Moderate(+++) |
| MH-delivered DI vs. NCG | No downgrade | No downgrade | No downgrade | No downgrade | No downgrade | No upgrade | High(++++) |
| MH-delivered PA vs. NCG | No downgrade | No downgrade | No downgrade | No downgrade | No downgrade | Upgrade(+) | High(++++) |
| FTF-DI vs. NCG | Downgrade(-) | No downgrade | No downgrade | No downgrade | No downgrade | No upgrade | Moderate(+++) |
| FTF-MLI vs. FTF-DI | No downgrade | No downgrade | No downgrade | No downgrade | No downgrade | No upgrade | High(++++) |
| FTF-PA+DI vs. FTF-DI | No downgrade | No downgrade | Downgrade(-) | Downgrade(-) | No downgrade | No upgrade | Moderate(+++) |
| MH-delivered PA+DI vs. FTF-DI | No downgrade | No downgrade | No downgrade | No downgrade | No downgrade | No upgrade | High(++++) |
| FTF-PA vs. FTF-DI | No downgrade | No downgrade | Downgrade(-) | Downgrade(-) | No downgrade | No upgrade | Low(++) |
| MH-delivered DI vs. FTF-DI | No downgrade | No downgrade | No downgrade | No downgrade | No downgrade | No upgrade | High(++++) |
| MH-delivered PA vs. FTF-DI | No downgrade | No downgrade | No downgrade | No downgrade | No downgrade | No upgrade | High(++++) |
| FTF-MLI vs. MH-delivered PA | No downgrade | Downgrade(-) | No downgrade | No downgrade | No downgrade | No upgrade | Moderate(+++) |
| FTF-PA+DI vs. MH-delivered PA | No downgrade | No downgrade | No downgrade | No downgrade | No downgrade | No upgrade | High(++++) |
| MH-delivered PA+DI vs. MH-delivered PA | No downgrade | No downgrade | No downgrade | No downgrade | No downgrade | No upgrade | High(++++) |
| FTF-PA vs. MH-delivered PA | No downgrade | No downgrade | Downgrade(-) | No downgrade | Downgrade(-) | No upgrade | Low(++) |
| MH-delivered DI vs. MH-delivered PA | No downgrade | No downgrade | No downgrade | No downgrade | No downgrade | No upgrade | High(++++) |
| FTF-MLI vs. MH-delivered DI | No downgrade | No downgrade | No downgrade | No downgrade | No downgrade | No upgrade | High(++++) |
| FTF-PA+DI vs. MH-delivered DI | No downgrade | No downgrade | No downgrade | No downgrade | No downgrade | No upgrade | High(++++) |
| MH-delivered PA+DI vs. MH-delivered DI | No downgrade | No downgrade | No downgrade | No downgrade | No downgrade | No upgrade | High(++++) |
| FTF-PA vs. MH-delivered DI | No downgrade | No downgrade | No downgrade | No downgrade | No downgrade | No upgrade | High(++++) |
| FTF-MLI vs. FTF-PA | No downgrade | No downgrade | No downgrade | No downgrade | No downgrade | No upgrade | High(++++) |
| FTF-PA+DI vs.FTF- PA | No downgrade | No downgrade | No downgrade | Downgrade(-) | No downgrade | No upgrade | Moderate(+++) |
| MH-delivered PA+DI vs. FTF-PA | No downgrade | Downgrade(-) | No downgrade | No downgrade | No downgrade | No upgrade | Moderate(+++) |
| FTF-MLI vs. MH-delivered PA+DI | No downgrade | Downgrade(-) | No downgrade | No downgrade | No downgrade | No upgrade | Moderate(+++) |
| FTF-PA+DI vs. MH-delivered PA+DI | No downgrade | No downgrade | No downgrade | No downgrade | No downgrade | No upgrade | High(++++) |
| FTF-MLI vs. FTF-PA+DI | No downgrade | No downgrade | No downgrade | No downgrade | No downgrade | No upgrade | High(++++) |

Comparisons were initially rated as high-quality evidence(four plus:++++) and were downgraded accordingly, based on study limitations, imprecision, inconsistency, indirectness, and publication bias.

a, study limitation: concerns about selection bias, performance bias, detection bias, attrition bias, reporting bias or other bias;

b, imprecision: concerns about sample size was insufficient or if imprecise estimates of wide confidence interval were generated in this comparison;

c, inconsistency: concerns about the study heterogeneity, especially the local inconsistency was found between direct and indirect evidence among the comparisons;

d, indirectness: concerns about there were heterogeneity observed based on four domains, namely, differences in populations, interventions, outcome measures, indirect comparisons and the combined effect of the four types of indirectness;

e, publication bias: concerns about publication bias due to an asymmetrical funnel plot was generated.

Each item was rated as no downgrade, downgrade one level(serious) or downgrade two levels(very serious). Upgrade due to large effect(large: upgrade one level, +1; very large: upgrade two level, +2), dose response(evidence of a gradient: upgrade one level, +1), or all plausible residual confounding(would reduce a demonstrated effect: upgrade one level, +1; would suggest a spurious effect if no effect was observed: upgrade two level, +2). All comparison were rated as four levels of evidence: High, We are very confident that the true effect lies close to that of the estimate of the effect(four plus:++++); Moderate, We are moderately confident in the effect estimate: The true effect is likely to be close to the estimate of the effect, but there is a possibility that it is substantially different(three plus:+++); Low, Our confidence in the effect estimate is limited: The true effect may be substantially different from the estimate of the effect(two plus:++); Very low, We have very little confidence in the effect estimate: The true effect is likely to be substantially different from the estimate of effect(one plus:+).

Reference available from: Balshem H, Helfand M, HJ Schünemann, et al. GRADE guidelines: 3. Rating the quality of evidence[J]. Journal of clinical epidemiology, 2011, 64(4):401-406.

**Table. S13 Risk of bias for individual study**

| **ROB items    Publications** | Random sequence generation(Selection bias) | Allocation concealment(Selection bias) | Blinding of participants &personnel(Performance bias) | Blinding of outcome assessors (Detection bias) | Incomplete outcome data (Attrition bias) | Selective reporting (Reporting bias) | Other bias |
| --- | --- | --- | --- | --- | --- | --- | --- |
| Christiansen M, 2013 | Low | Unclear | Unclear | Unclear | High | Low | Unclear |
| Macias-Cervantes, 2009 | Unclear | Low | Low | Low | Low | Unclear | Low |
| Kriemler, 2010 | Low | Low | Low | Low | Low | Low | High |
| Li, 2010 | Low | Unclear | Unclear | Low | Low | Low | Low |
| Lubans, 2011 | Low | Unclear | Unclear | Unclear | Unclear | Low | Unclear |
| Reed, 2008 | Unclear | Low | Low | Unclear | Unclear | Low | Unclear |
| Robbins, 2006 | Low | Unclear | Low | Unclear | Unclear | Low | Unclear |
| Vizcaíno, 2008 | Low | Low | Unclear | Low | Low | Low | Low |
| Smith, 2014 | Low | Low | Unclear | Low | Low | Low | Low |
| Velez, 2010 | Low | Low | Low | Unclear | Low | Low | Low |
| Weeks, 2012 | Low | Unclear | Low | Low | Low | Low | High |
| Howe, 2010 | Low | Unclear | Low | Low | Low | Low | High |
| Vizcaíno, 2014 | Low | Low | Unclear | Unclear | Unclear | Low | Unclear |
| Mihas, 2009 | Low | Low | Low | Unclear | Low | Unclear | Unclear |
| Paineau, 2008 | Low | Low | Low | Low | Low | Low | High |
| Rosário, 2012 | Low | Unclear | Low | Low | Unclear | Low | Low |
| Sichieri, 2009 | Low | Low | Unclear | Unclear | Unclear | Unclear | Unclear |
| Ebbeling, 2006 | Low | Low | Low | Unclear | Unclear | Low | Unclear |
| James, 2004 | Low | High | Low | High | Low | Unclear | Unclear |
| Andrade, 2014 | Low | Low | High | Low | Low | Unclear | Unclear |
| Black, 2010 | Low | Unclear | Low | Unclear | Low | Low | Low |
| Bohnert, 2013 | Unclear | Unclear | Low | Low | Unclear | Low | Low |
| Brown, 2013 | Low | Unclear | Unclear | Unclear | Unclear | Low | Unclear |
| Cao, 2015 | Low | Unclear | Unclear | Low | Unclear | Low | Unclear |
| Chen, 2010 | Low | High | Unclear | Unclear | High | Low | Unclear |
| Dewar, 2013 | Low | Unclear | Unclear | Low | Low | Low | Unclear |
| Ezendam, 2012 | Low | Unclear | High | Low | Unclear | Unclear | Unclear |
| Gentile, 2009 | Low | Low | Unclear | Low | Low | Low | Low |
| Grydeland, 2014 | Low | Low | Low | Unclear | Low | Low | Unclear |
| Mourad, 2013 | Low | Unclear | Low | Unclear | Unclear | Low | Unclear |
| Jansen, 2011 | Low | Low | Unclear | Unclear | Low | Unclear | Unclear |
| Johnston, 2013 | Low | Low | Low | Unclear | Low | Unclear | Unclear |
| Kain, 2014 | Unclear | High | Unclear | Unclear | Unclear | Low | Unclear |
| Klesges, 2010 | Low | Unclear | Unclear | Unclear | Unclear | Low | Unclear |
| Llargues, 2011 | Low | High | High | Unclear | Unclear | Low | Unclear |
| Llargués, 2012 | Low | High | Unclear | Low | Unclear | Low | Unclear |
| Hrafnkelsson, 2014 | Low | Unclear | Unclear | Unclear | Unclear | Low | Unclear |
| Magnusson, 2012 | Low | Unclear | Unclear | Low | Unclear | Low | Unclear |
| Meng, 2013 | Low | Unclear | Low | Low | Low | Unclear | Low |
| Morgan, 2011 | Low | Unclear | Low | Unclear | Unclear | Low | Unclear |
| Nollen, 2014 | Low | High | Unclear | Low | Unclear | Low | Unclear |
| Heer, 2011 | Low | Low | Low | Unclear | Unclear | Low | Unclear |
| Safdie, 2013 | Low | Low | Low | Low | Low | Low | High |
| Santos, 2014 | Low | Low | Unclear | Low | Unclear | Unclear | Unclear |
| SEVİNÇ, 2011 | Low | Unclear | Unclear | Unclear | Unclear | Low | Unclear |
| Siegrist, 2013 | Low | Unclear | Unclear | Unclear | Low | Unclear | Unclear |
| Peralta, 2009 | Low | Low | Low | Unclear | Low | Low | Low |
| Singh, 2009 | Low | High | High | High | Unclear | Low | Unclear |
| Robinson, 2003 | Low | Unclear | Low | Low | Low | Low | High |
| Story, 2003 | Low | Unclear | Low | Low | Low | Low | High |
| Lubans, 2012 | Low | High | Low | Unclear | Low | Low | Low |
| Lubans, 2014 | Low | High | Unclear | Low | Unclear | Unclear | Unclear |
| Niet, 2012 | Low | Low | Unclear | Unclear | Low | Low | Unclear |
| Davis, 2013 | Low | Unclear | High | Low | Low | Low | Unclear |
| Morgan, 2012 | Low | High | Unclear | Low | Unclear | Low | Unclear |
| Muckelbauer, 2009 | Low | Low | Unclear | Low | Unclear | Unclear | Unclear |
| Lubans, 2012 | Low | Unclear | High | Low | Low | Unclear | Unclear |
| Kalarchian, 2009 | Low | Unclear | Unclear | Low | Unclear | Low | Unclear |
| Wafa, 2011 | Low | Unclear | Unclear | Low | Low | Low | Low |
| Kalavainen, 2007 | Low | Unclear | Unclear | High | Unclear | Low | Unclear |
| Sacher, 2010 | Low | Low | Unclear | Low | Low | Low | Low |
| Janicke, 2008 | Low | Unclear | Low | Low | Low | Low | High |
| Davis, 2009 | Low | Unclear | Unclear | Low | Low | Low | Low |
| Saelens, 2002 | Low | Unclear | Unclear | Low | Low | Unclear | Unclear |
| Nemet, 2005 | Low | Unclear | High | Unclear | Low | Low | Unclear |
| Savoye, 2007 | Low | High | High | Low | Low | Low | Unclear |
| Shaw, 2009 | Low | High | Low | Unclear | Low | Low | Low |
| Hughes, 2008 | Low | Low | Low | Low | Low | Unclear | Low |
| Balagopal, 2003 | Low | Low | Unclear | Low | Unclear | Low | Unclear |
| Jiang, 2005 | Low | Unclear | Low | Low | Low | Low | High |
| McCallum, 2007 | Low | Low | Unclear | Low | Unclear | Unclear | Low |
| Shelton, 2007 | Low | Unclear | High | Low | Low | Low | Unclear |
| Tsiros, 2008 | Low | High | Unclear | Unclear | Low | Unclear | Low |
| Kitzman-Ulrich, 2009 | Low | Unclear | Low | Unclear | Low | Low | Low |
| Wake, 2009 | Low | Low | Low | Low | Low | Unclear | Low |
| Reinehr, 2010 | Low | Low | Unclear | Low | Low | Unclear | Unclear |
| Boudreau, 2013 | Low | Unclear | Unclear | Unclear | Unclear | Low | Unclear |
| Backlund, 2011 | Low | Unclear | Low | Unclear | Low | Low | Unclear |
| Waling, 2010 | Low | Unclear | Unclear | Low | Low | Low | Low |
| Boodai, 2014 | Low | Low | Unclear | High | Low | Low | Unclear |
| Croker, 2012 | Low | Unclear | Unclear | Low | Low | Low | Unclear |
| Davoli, 2013 | Low | Unclear | Low | Low | Low | Low | High |
| DeBar, 2012 | Low | Unclear | Unclear | Low | Unclear | Low | Unclear |
| Díaz, 2010 | Low | Low | Unclear | Low | Low | Low | Low |
| Ford, 2009 | Low | Unclear | Unclear | Unclear | Unclear | Low | Unclear |
| Gong, 2014 | Low | Unclear | Unclear | Low | Low | Low | Low |
| Gourlan, 2013 | Low | Unclear | Unclear | Unclear | Low | Low | Unclear |
| Grey, 2009 | Low | Unclear | Unclear | Low | Unclear | Unclear | Unclear |
| Hofsteenge, 2014 | Low | Unclear | High | Low | Low | Unclear | Unclear |
| Johnston, 2007 | Low | Unclear | Low | High | Low | Low | Unclear |
| Kalavainen, 2012 | Low | High | High | Low | Unclear | Low | Unclear |
| Maddison, 2014 | Low | Low | Low | Low | Low | Low | High |
| Nguyen, 2012 | Low | High | Unclear | Unclear | Unclear | Unclear | Unclear |
| Pakpour, 2015 | Low | Low | Unclear | Low | Low | Low | Low |
| Pbert, 2013 | Low | Unclear | Low | High | Low | Low | Unclear |
| Savoye, 2011 | Low | Low | Low | Low | Unclear | Low | Low |
| Savoye, 2014 | Low | Low | Unclear | Low | Unclear | Unclear | Unclear |
| Vos, 2011 | Low | Low | Unclear | Unclear | Unclear | Low | Unclear |
| Williamson, 2005 | Low | Unclear | Unclear | Low | Unclear | Low | Unclear |
| Wright, 2014 | Low | Unclear | Low | Unclear | Low | Low | Low |
| Saelens, 2011 | Low | Low | Low | Low | Unclear | Low | Low |
| Prado, 2010 | Low | High | Low | Unclear | Low | Low | Low |
| Staiano, 2018 | Low | Low | Low | Low | Low | Low | High |
| Pbert, 2016 | Low | Low | High | High | Unclear | Low | Unclear |
| Ochoa-Avilés, 2017 | Low | Low | Unclear | Unclear | Low | Unclear | Unclear |
| Nayak, 2016 | Low | Unclear | Unclear | Unclear | Unclear | Low | Unclear |
| Morell-Azanza, 2019 | Low | Unclear | Low | Unclear | Low | Unclear | Unclear |
| Lloyd, 2017 | Low | Low | Unclear | Unclear | Low | Unclear | Unclear |
| Liu, 2018 | Low | Low | Unclear | Unclear | Low | Unclear | Unclear |
| Li, 2019 | Low | Low | Unclear | Low | Low | Low | Unclear |
| Larsen, 2016 | Low | Unclear | Low | Unclear | Unclear | Low | Unclear |
| Kim, 2016 | Low | Unclear | Low | Low | Low | Low | High |
| Hao, 2019 | Low | Unclear | High | High | Unclear | Low | Unclear |
| Chen, 2019 | Low | Unclear | Unclear | Unclear | Unclear | Low | Unclear |
| Bruñó, 2018 | Low | Low | Unclear | High | Low | Unclear | Unclear |
| Ahmad, 2018 | Low | Low | Unclear | Low | Low | Low | Low |
| Yusop, 2018 | Low | Low | Unclear | Unclear | Unclear | Unclear | Unclear |
| Xu, 2017 | Low | Unclear | Unclear | Unclear | Unclear | Unclear | Unclear |
