# Supplementary material for: Face-to-face physical activity incorporated into dietary intervention for overweight/obesity in children and adolescents: a Bayesian network meta-analysis
Source: BMC Med. 2022 Sep 2;20:325. doi: 10.1186/s12916-022-02462-6 (PMC9438135; doi:10.1186/s12916-022-02462-6)
Supplement: Supplementary file 4 — Additional file 4: Figure. S1-2 Risk of Bias graph; Figure. S3-4 Network of evidence of all the trials based on the outcomes of WC and PBF, and the Efficacy of varied treatments compared with named control group; Figure. S5-8 Funnel plots based on the four outcomes; Figure. S9-12 SUCRA plots based on the four outcomes; Figure. S13-16 Inconsistency plots based on the four outcomes; Figure. S17-23 Network evidence of sensitivity analysis based on the four outcomes(12 and 24 months); Figure. S24-30 SUCRA plots of sensitivity analysis based on the four outcomes(12 and 24 months); Figure. S31-37 Funnel plots of sensitivity analysis based on the four outcomes(12 and 24 months). [file 12916_2022_2462_MOESM4_ESM.docx]

**Figure. S1** **Risk of Bias graph**

**Figure. S2 Risk of bias summary**

| Risk of bias | Random sequence generation(Selection bias) | Allocation concealment(Selection bias) | Blinding of participants & personnel (Performance bias) | Blinding of outcome assessors (Detection bias) | Incomplete outcome data(Attrition bias) | Selective reporting (Reporting bias) | Other bias |
| --- | --- | --- | --- | --- | --- | --- | --- |
| Unclear risk | 3.39% | 48.31% | 51.69% | 40.68% | 41.53% | 26.27% | 63.56% |
| Low risk | 96.61% | 38.14% | 37.29% | 51.69% | 56.78% | 73.73% | 25.42% |
| High risk | 0.00% | 13.56% | 11.02% | 7.63% | 1.69% | 0.00% | 11.02% |


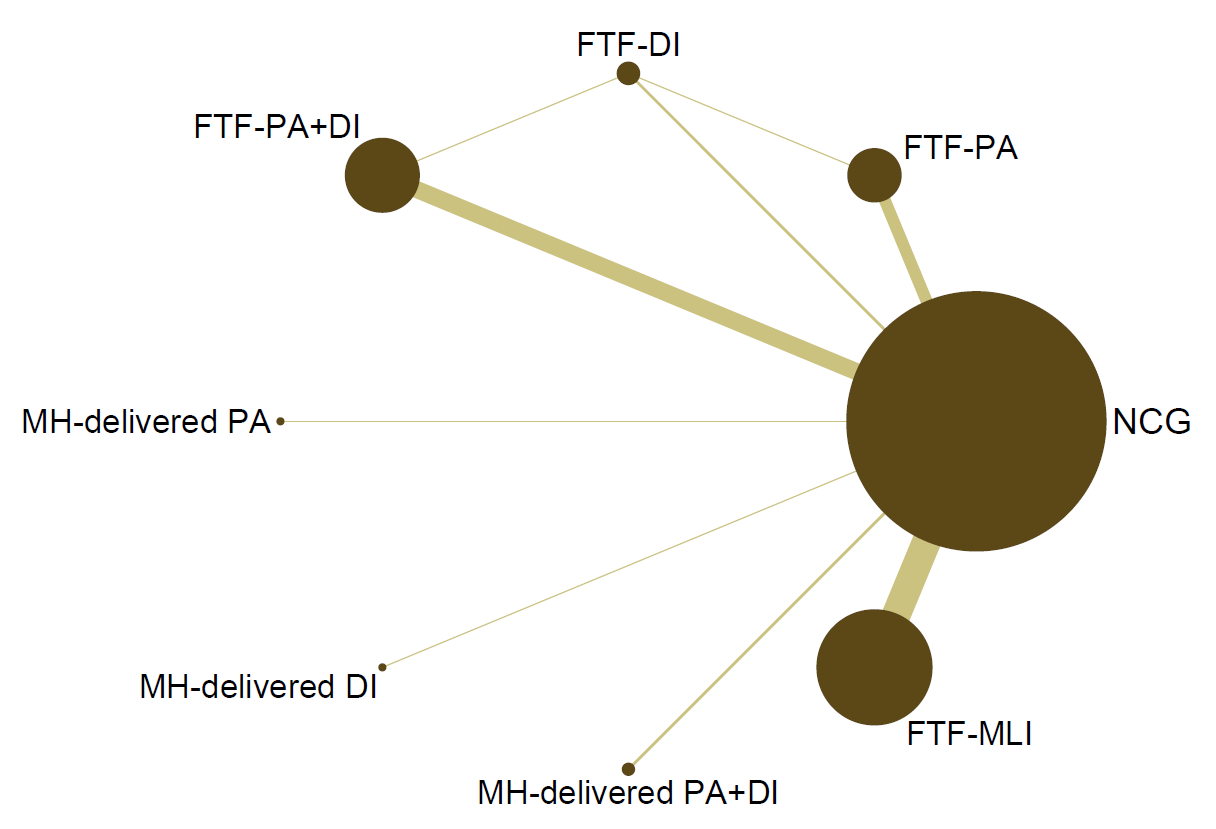
**Figure. S3 Network of evidence of all the trials based on the outcome of WC, and the Efficacy of varied treatments compared with named control group.**

Each node represented a treatment and its size depended on the number of patients that is directly examined. The nodes were joined by different thickness lines which generated to show whether there existed a direct relationship between treatments and the thickness was weighted according to the available direct evidence between them. CrIs, Credible intervals; DI, Dietary intervention; FTF, Face-to-face; MH-delivered, Mobile health-based; MLI, Muti-lifestyle intervention; NCG, Named control group; NR, Not reported; PA, Physical activity; SUCRA, the surface under the cumulative ranking curve.


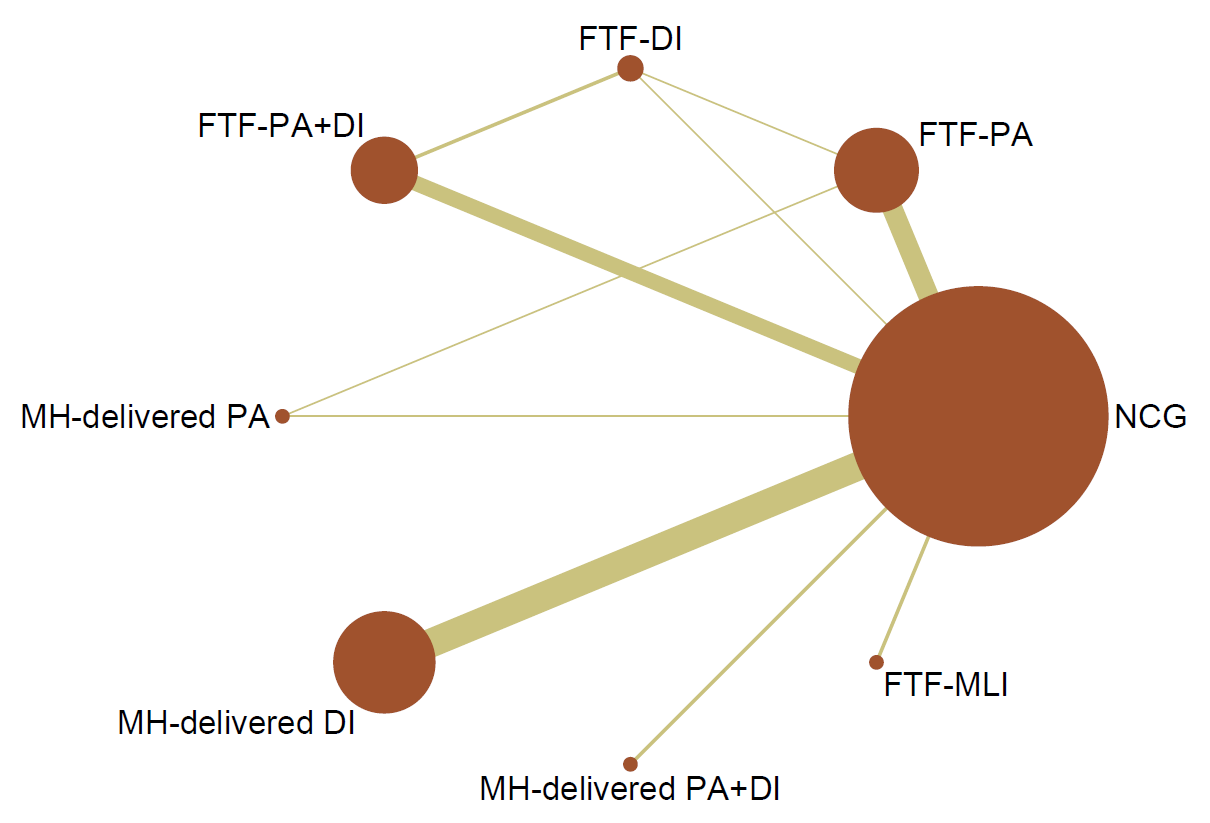
**Figure. S4 Network of evidence of all the trials based on the outcome of PBF, and the Efficacy of varied treatments compared with named control group.**

Each node represented a treatment and its size depended on the number of patients that is directly examined. The nodes were joined by different thickness lines which generated to show whether there existed a direct relationship between treatments and the thickness was weighted according to the available direct evidence between them. CrIs, Credible intervals; DI, Dietary intervention; FTF, Face-to-face; MH-delivered, Mobile health-based; MLI, Muti-lifestyle intervention; NCG, Named control group; NR, Not reported; PA, Physical activity; SUCRA, the surface under the cumulative ranking curv

**
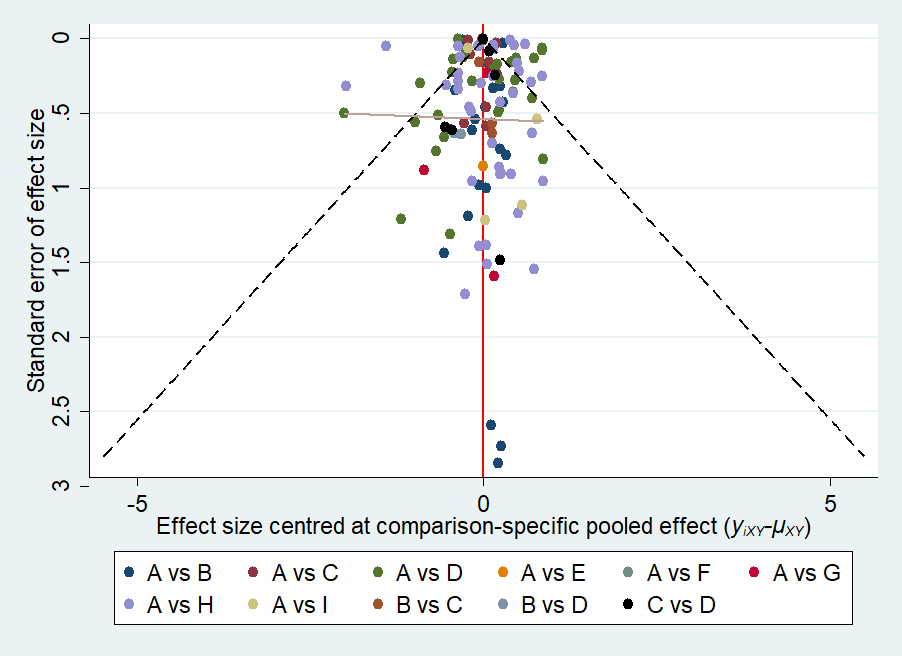
Figure. S5 Funnel plot based on the BMI outcome**

A: NCG, Control group(Treatment as usual, wait-list); B: FTF-PA; C: FTF-DI; D: FTF-PA+DI; E: MH-delivered PA; F: MHI-delivered DI; G: MH-delivered PA+DI; H: FTF-MLI; I: MH-delivered MLI; DI, Dietary intervention; FTF, Face-to-face; MH-delivered, Mobile health-based; MLI, Muti-lifestyle intervention; NCG, Named control group; PA, Physical activity.

**Figure. S6 Funnel plot based on the BMI Z-score outcome**


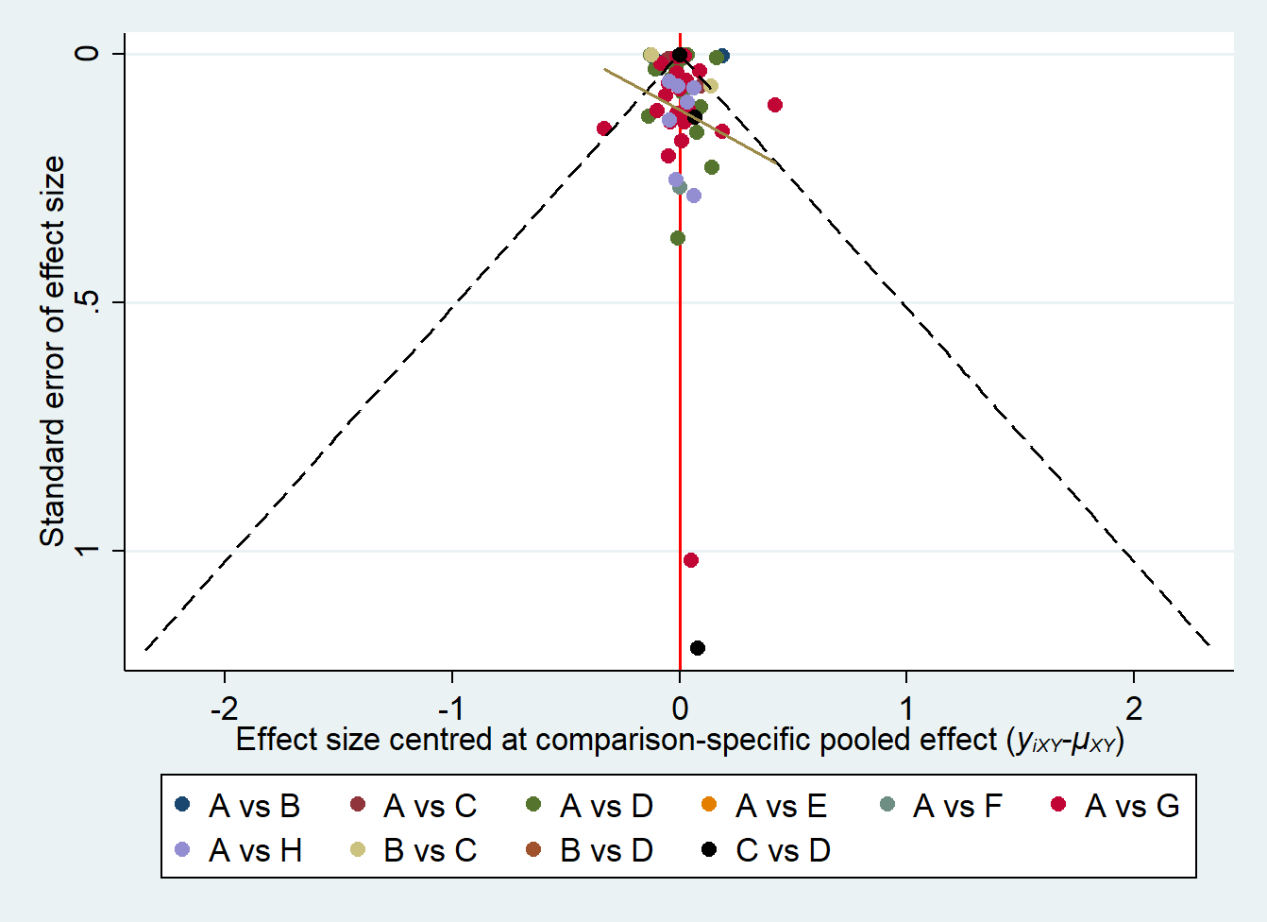


A: NCG, Control group(Treatment as usual, wait-list); B: FTF-PA; C: FTF-DI; D: FTF-PA+DI; E: MH-delivered PA; F: MH-delivered DI; G: MH-delivered PA+DI; H: FTF-MLI; MLI; DI, Dietary intervention; FTF, Face-to-face; MH-delivered, Mobile health-based; MLI, Muti-lifestyle intervention; NCG, Named control group; PA, Physical activity.

**Figure. S7 Funnel plot based on the WC outcome**


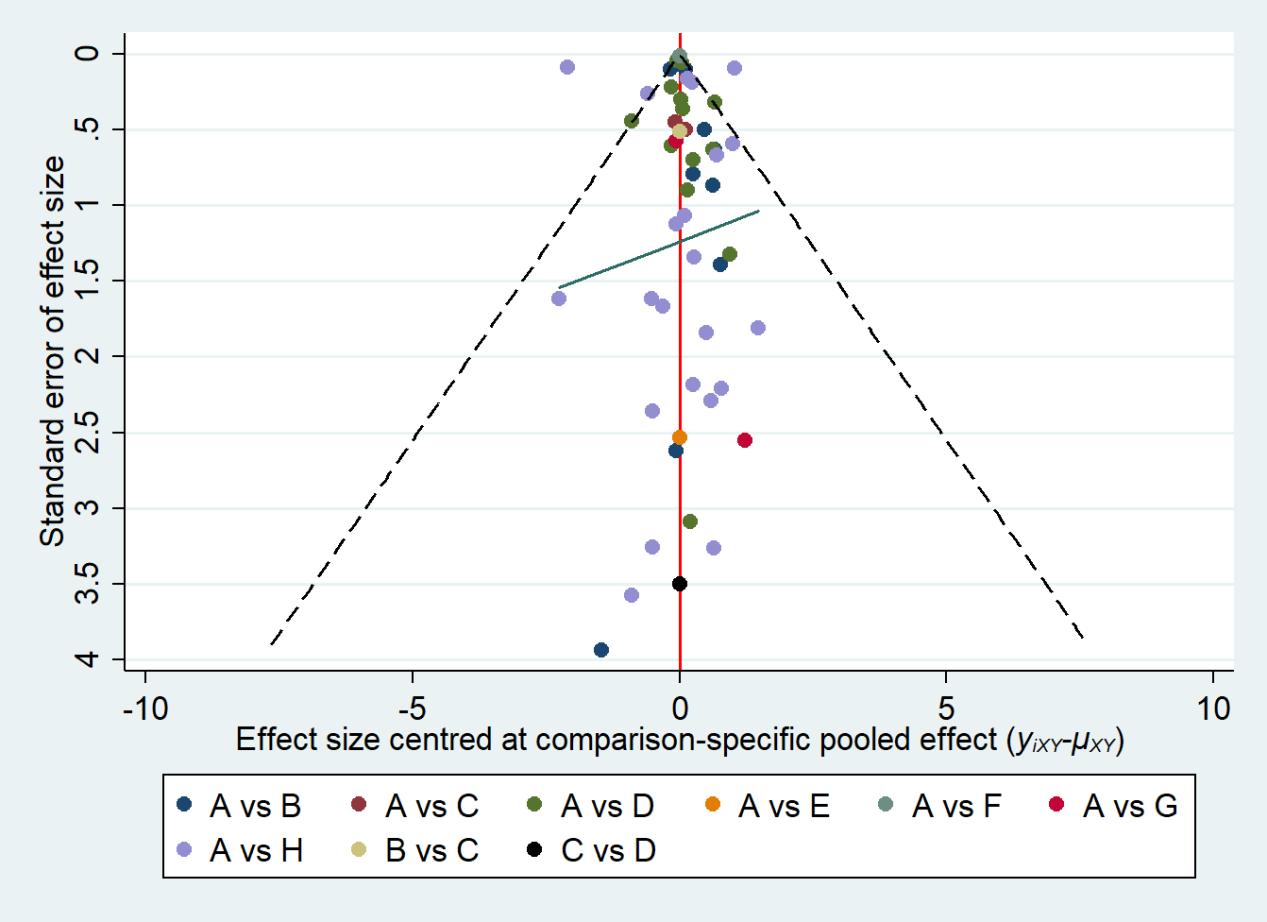


A: NCG, Control group(Treatment as usual, wait-list); B: FTF-PA; C: FTF-DI; D: FTF-PA+DI; E: MH-delivered PA; F: MH-delivered DI; G: MH-delivered PA+DI; H: FTF-MLI; DI, Dietary intervention; FTF, Face-to-face; MH-delivered, Mobile health-based; MLI, Muti-lifestyle intervention; NCG, Named control group; PA, Physical activity.

**Figure. S8 Funnel plot based on the PBF outcome**


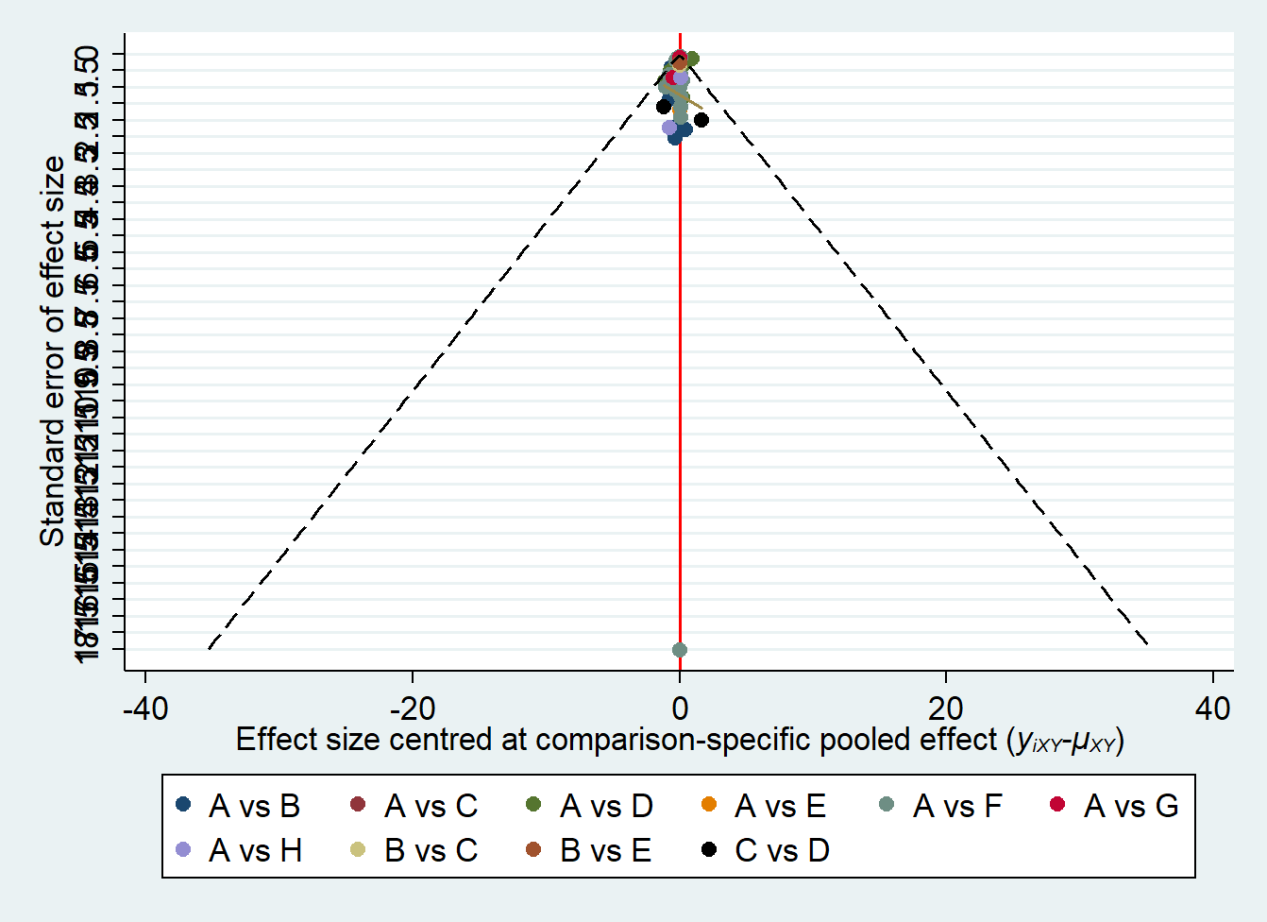
A: NCG, Control group(Treatment as usual, wait-list); B: FTF-PA; C: FTF-DI; D: FTF-PA+DI; E: MH-delivered PA; F: MH-delivered DI; G: MH-delivered PA+DI; H: FTF-MLI; DI, Dietary intervention; FTF, Face-to-face; MH-delivered, Mobile health-based; MLI, Muti-lifestyle intervention; NCG, Named control group; PA, Physical activity.

**Figure. S9 SUCRA plot based on the BMI outcome**


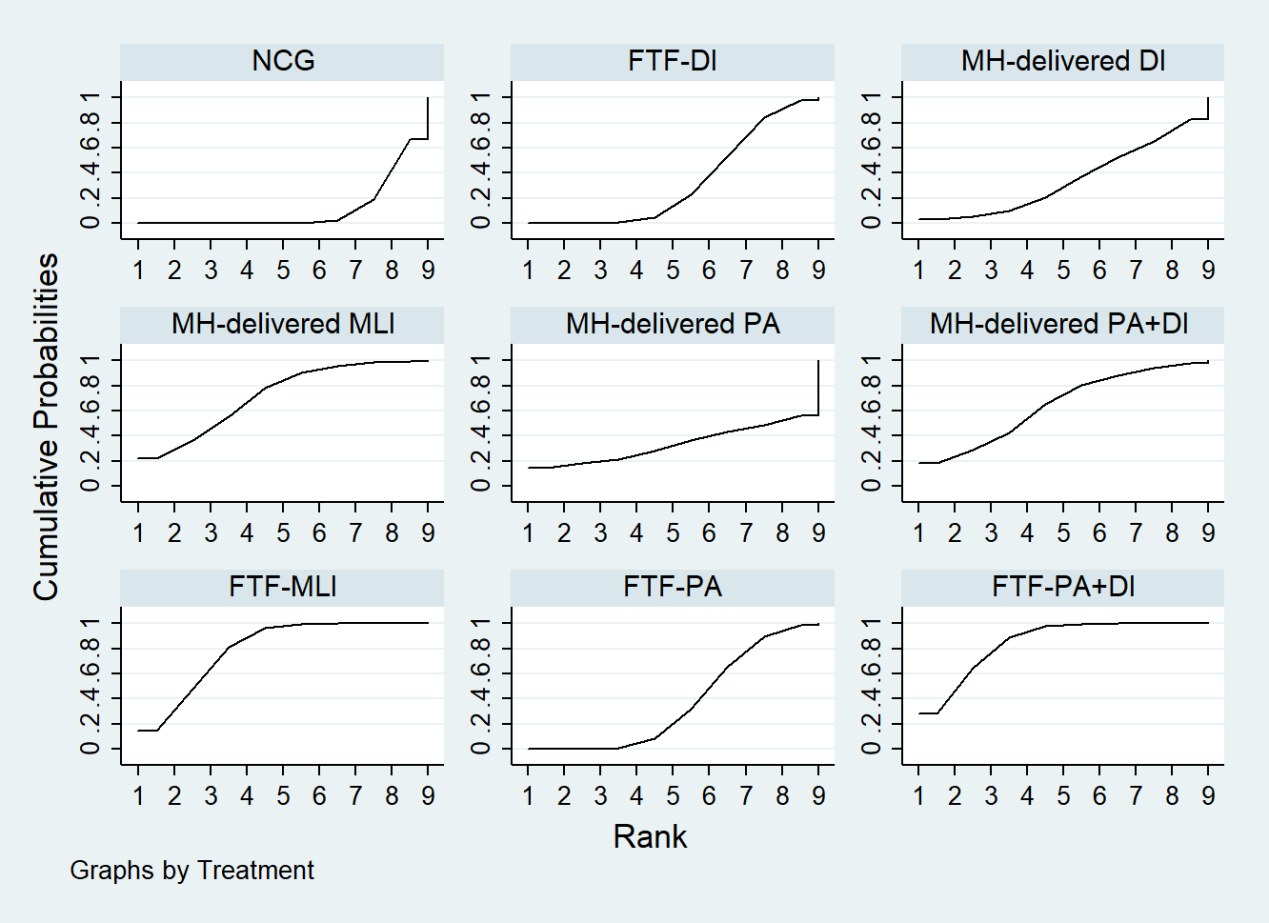
DI, Dietary intervention; FTF, Face-to-face; MH-delivered, Mobile health-based; MLI, Muti-lifestyle intervention; NCG, Named control group; PA, Physical activity.

**Figure. S10 SUCRA plot based on the BMI Z-score outcome**


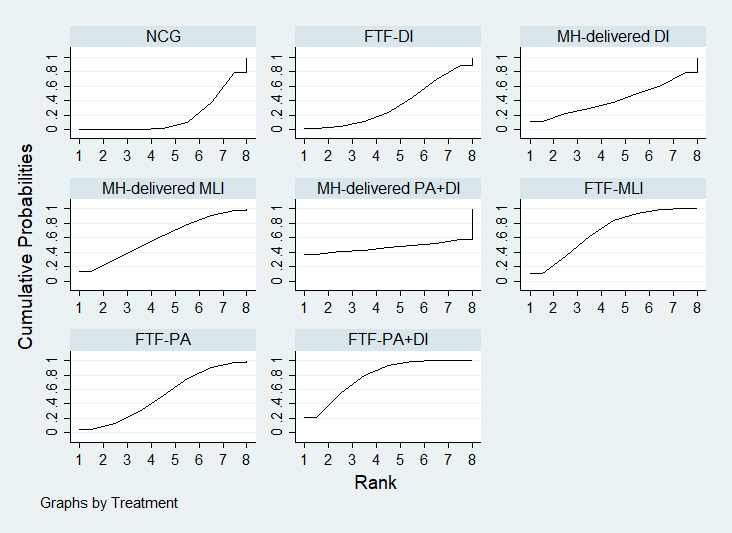
DI, Dietary intervention; FTF, Face-to-face; MH-delivered, Mobile health-based; MLI, Muti-lifestyle intervention; NCG, Named control group; PA, Physical activity.

**Figure. S11 SUCRA plot based on the WC outcome**


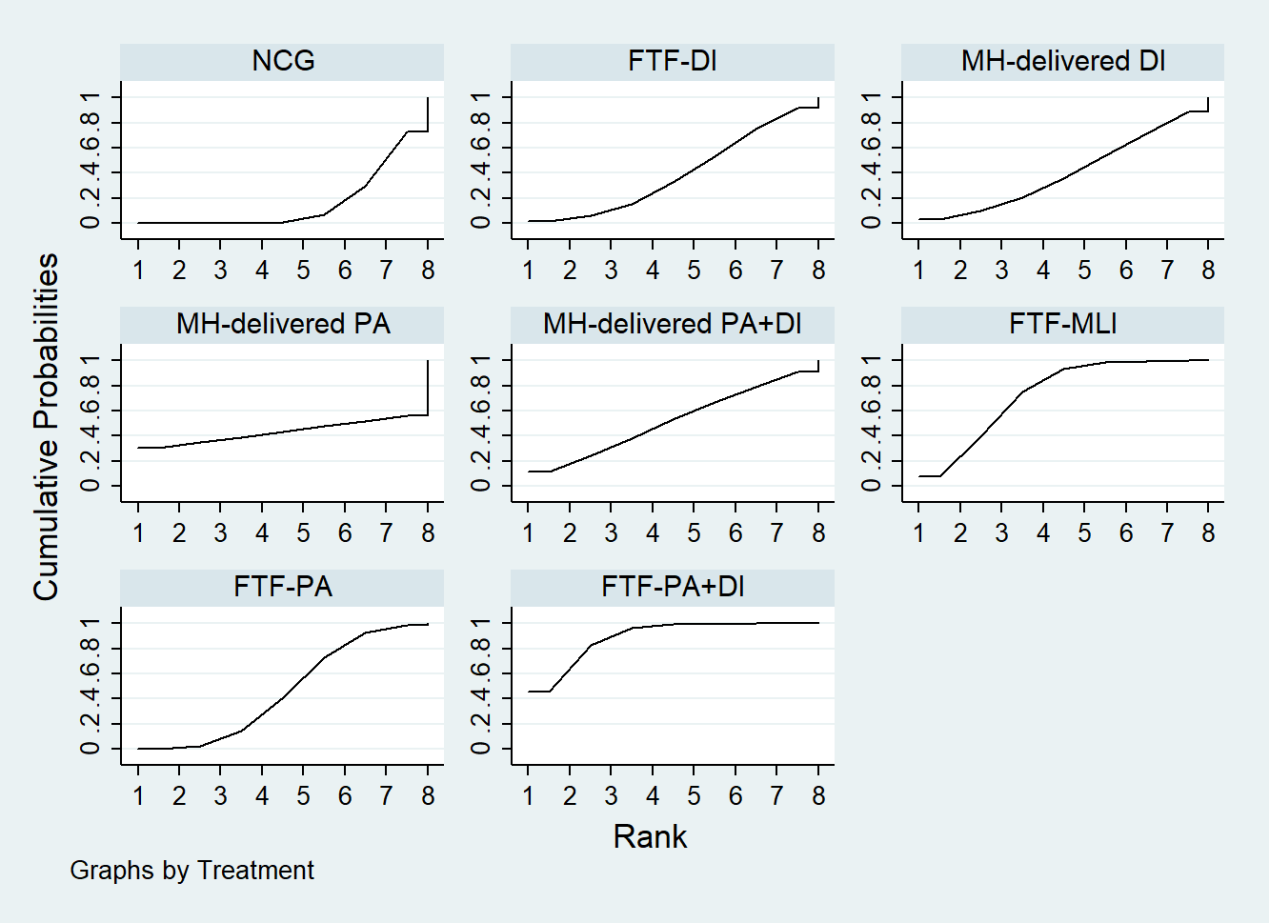
DI, Dietary intervention; FTF, Face-to-face; MH-delivered, Mobile health-based; MLI, Muti-lifestyle intervention; NCG, Named control group; PA, Physical activity.

**Figure. S12 SUCRA plot based on the PBF outcome**


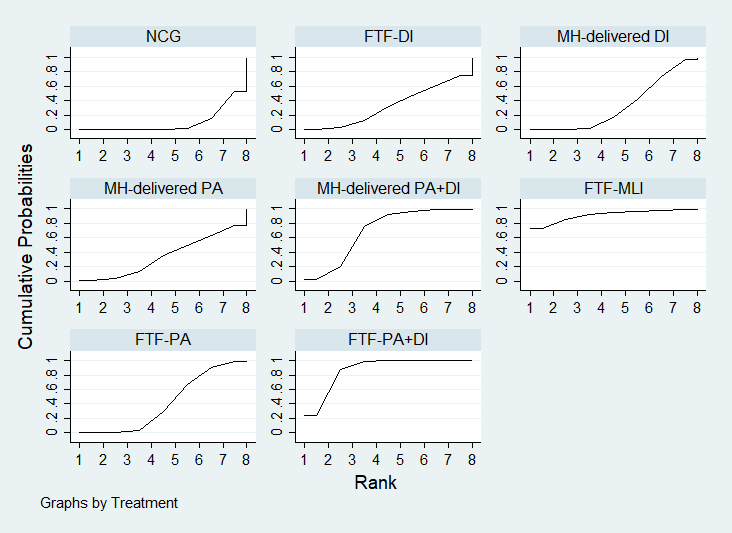
DI, Dietary intervention; FTF, Face-to-face; MH-delivered, Mobile health-based; MLI, Muti-lifestyle intervention; NCG, Named control group; PA, Physical activity.

**Figure. S13 Inconsistency plot based on the BMI outcome**


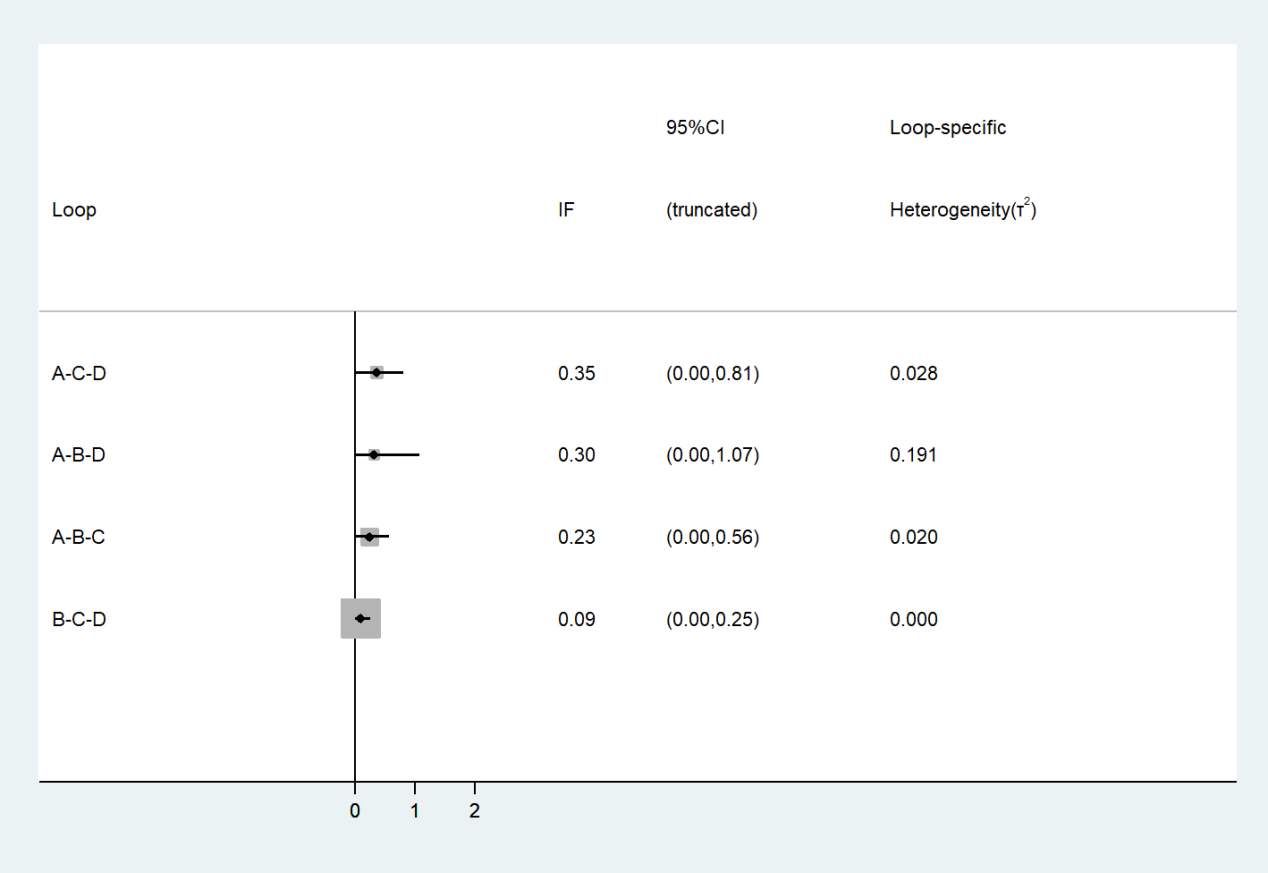
A: NCG, Control group(Treatment as usual, wait-list); B: FTF-PA; C: FTF-DI; D: FTF-PA+DI; DI, Dietary intervention; FTF, Face-to-face; MH-delivered, Mobile health-based; MLI, Muti-lifestyle intervention; NCG, Named control group; PA, Physical activity.

**Figure. S14 Inconsistency plot based on the BMI Z-score outcome**


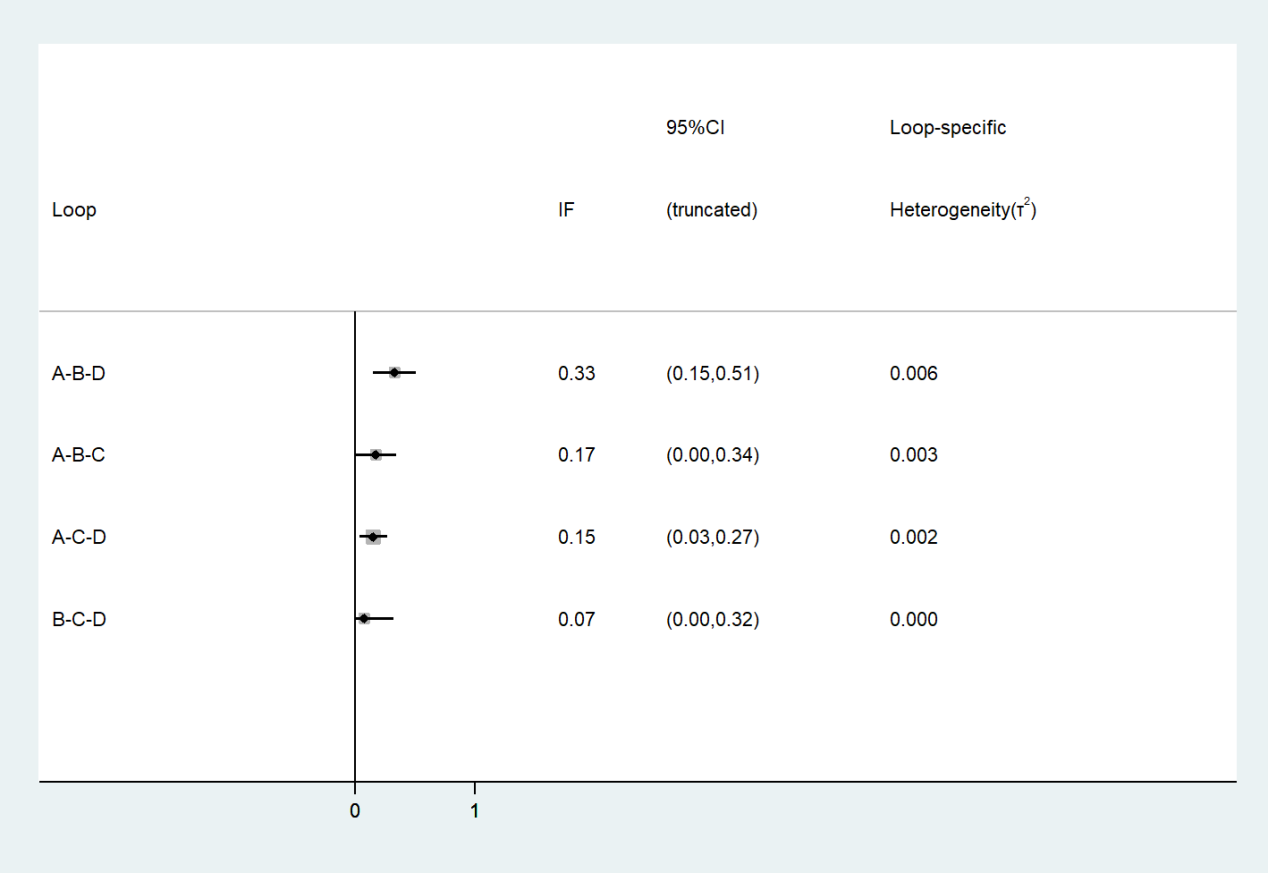
A: NCG, Control group(Treatment as usual, wait-list); B: FTF-PA; C: FTF-DI; D: FTF-PA+DI; DI, Dietary intervention; FTF, Face-to-face; MH-delivered, Mobile health-based; MLI, Muti-lifestyle intervention; NCG, Named control group; PA, Physical activity.

**Figure. S15 Inconsistency plot based on the WC outcome**


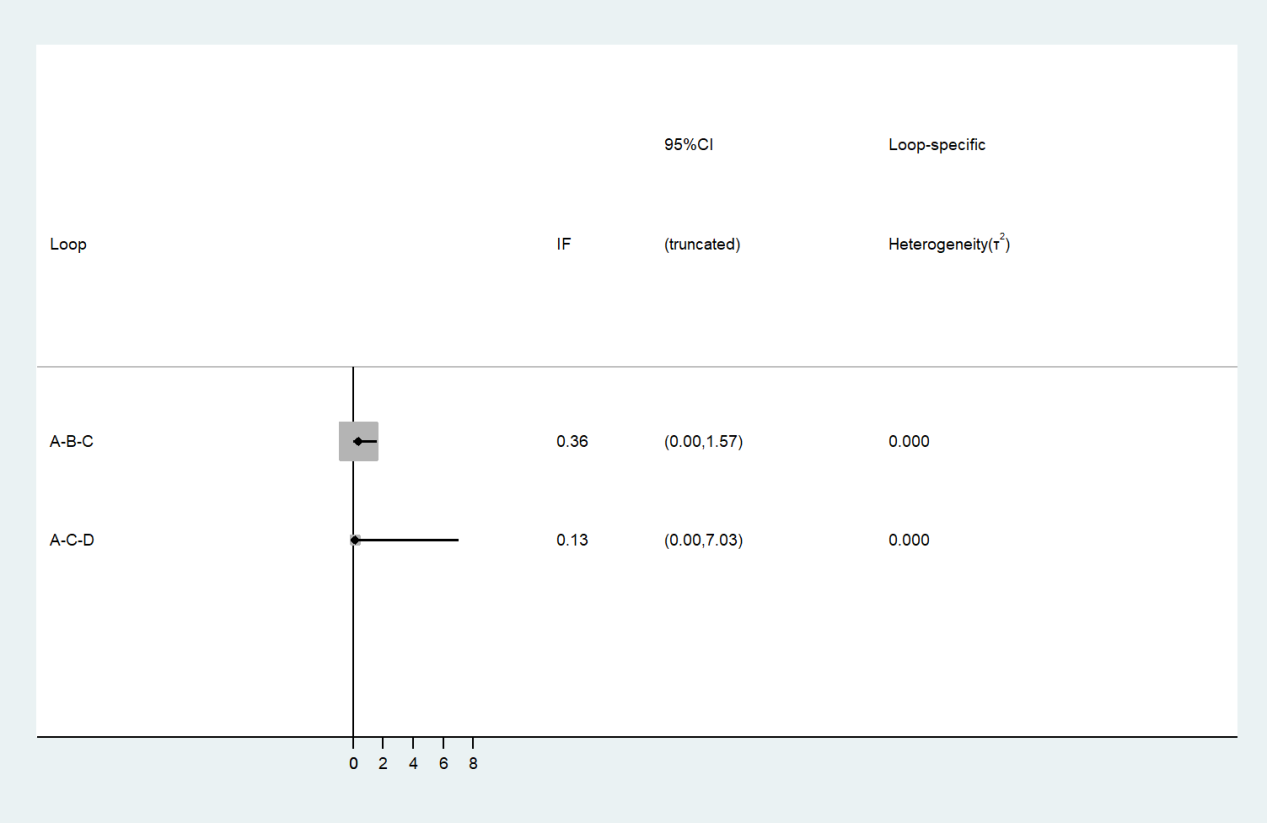
A: NCG, Control group(Treatment as usual, wait-list); B: FTF-PA; C: FTF-DI; D: FTF-PA+DI; DI, Dietary intervention; FTF, Face-to-face; MH-delivered, Mobile health-based; MLI, Muti-lifestyle intervention; NCG, Named control group; PA, Physical activity.

**Figure. S16 Inconsistency plot based on the PBF outcome**


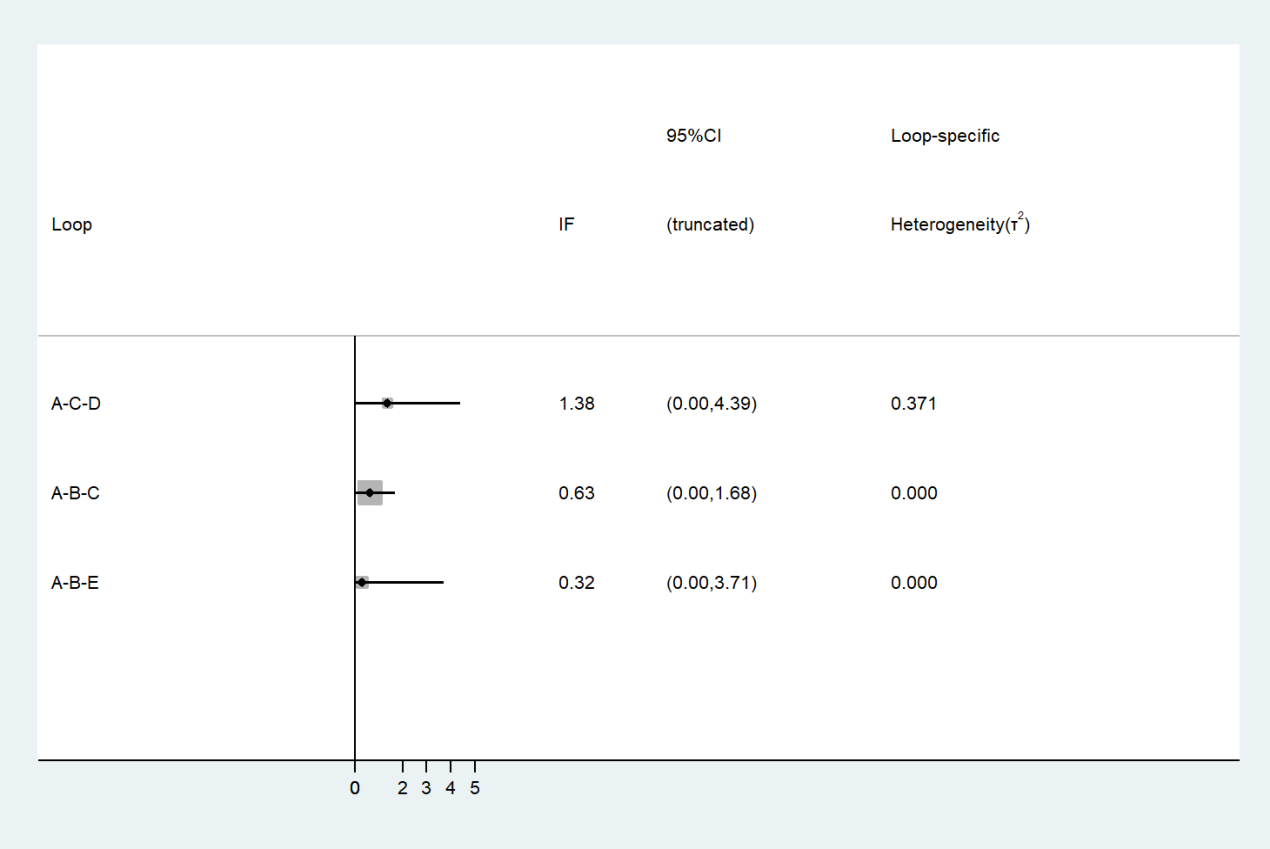
A: NCG, Control group(Treatment as usual, wait-list); B: FTF-PA; C: FTF-DI; D: FTF-PA+DI; E: MHI-delivered PA; DI, Dietary intervention; FTF, Face-to-face; MH-delivered, Mobile health-based; MLI, Muti-lifestyle intervention; NCG, Named control group; PA, Physical activity.

**Figure. S17 Network evidence of sensitivity analysis based on the outcome of BMI(12 months).**

DI, Dietary intervention; FTF, Face-to-face; MH-delivered, Mobile health-based; MLI, Muti-lifestyle intervention; NCG, Named control group; PA, Physical activity. **
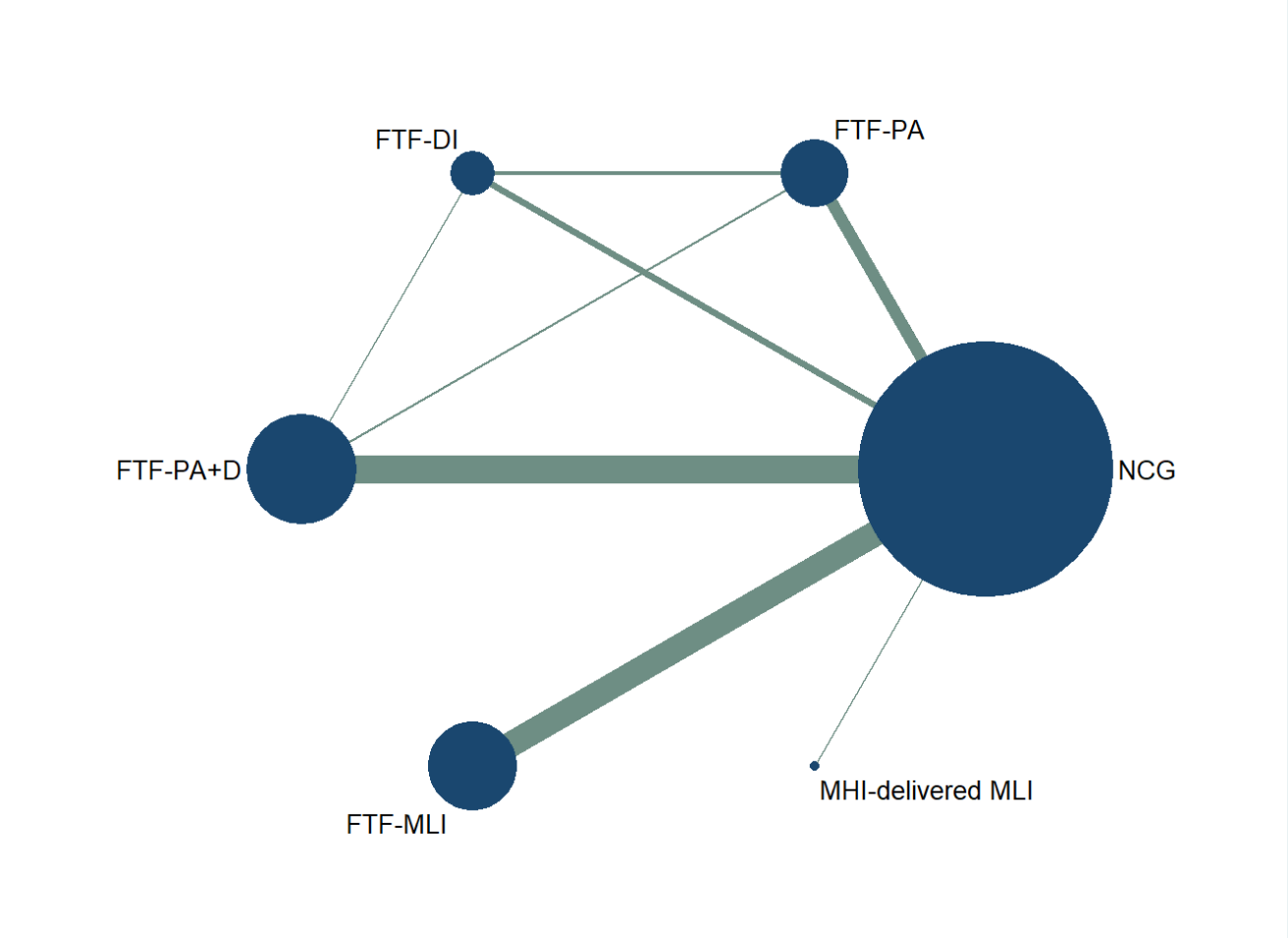
**


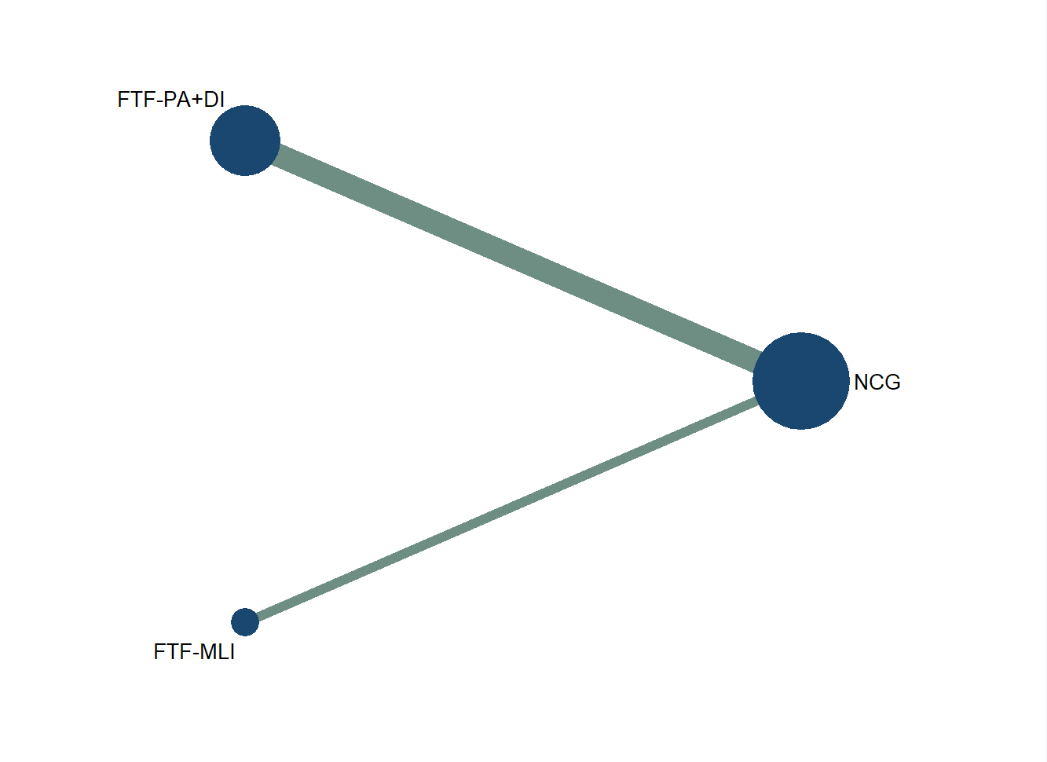
**Figure. S18 Network evidence of sensitivity analysis based on the outcome of BMI(24 months).**

DI, Dietary intervention; FTF, Face-to-face; MH-delivered, Mobile health-based; MLI, Muti-lifestyle intervention; NCG, Named control group; PA, Physical activity.

**Figure. S19 Network evidence of sensitivity analysis based on the outcome of BMI Z-score(12 months).**

DI, Dietary intervention; FTF, Face-to-face; MH-delivered, Mobile health-based; MLI, Muti-lifestyle intervention; NCG, Named control group; PA, Physical activity. **
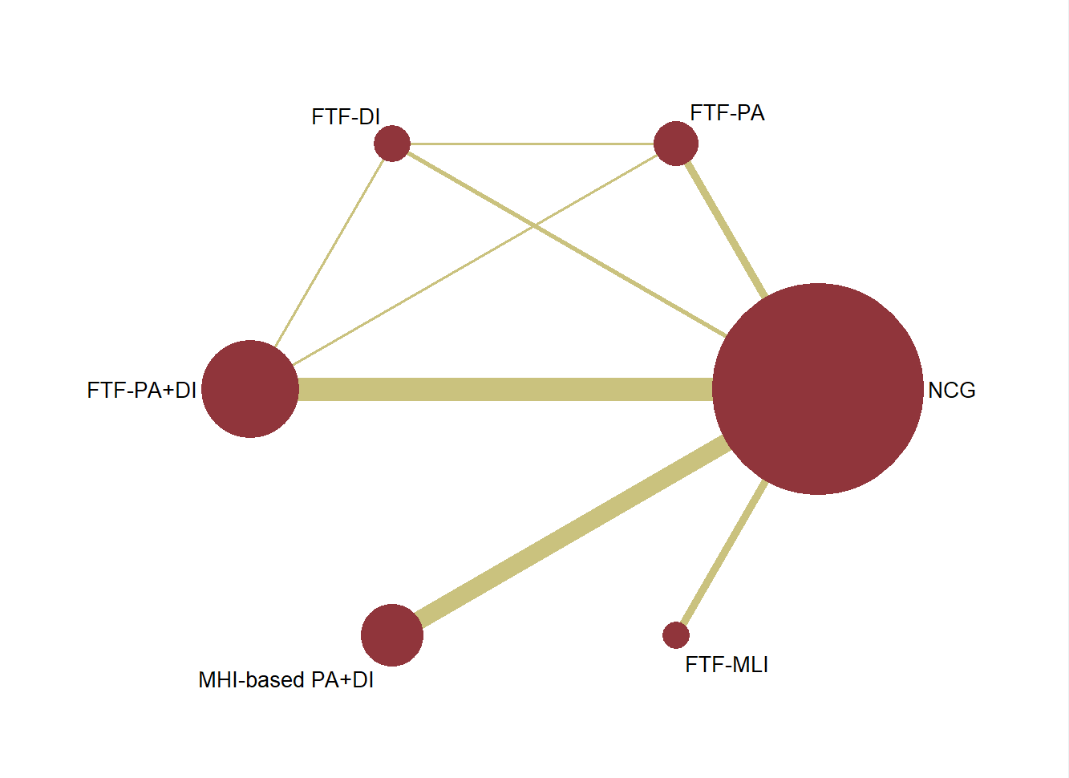
**

**
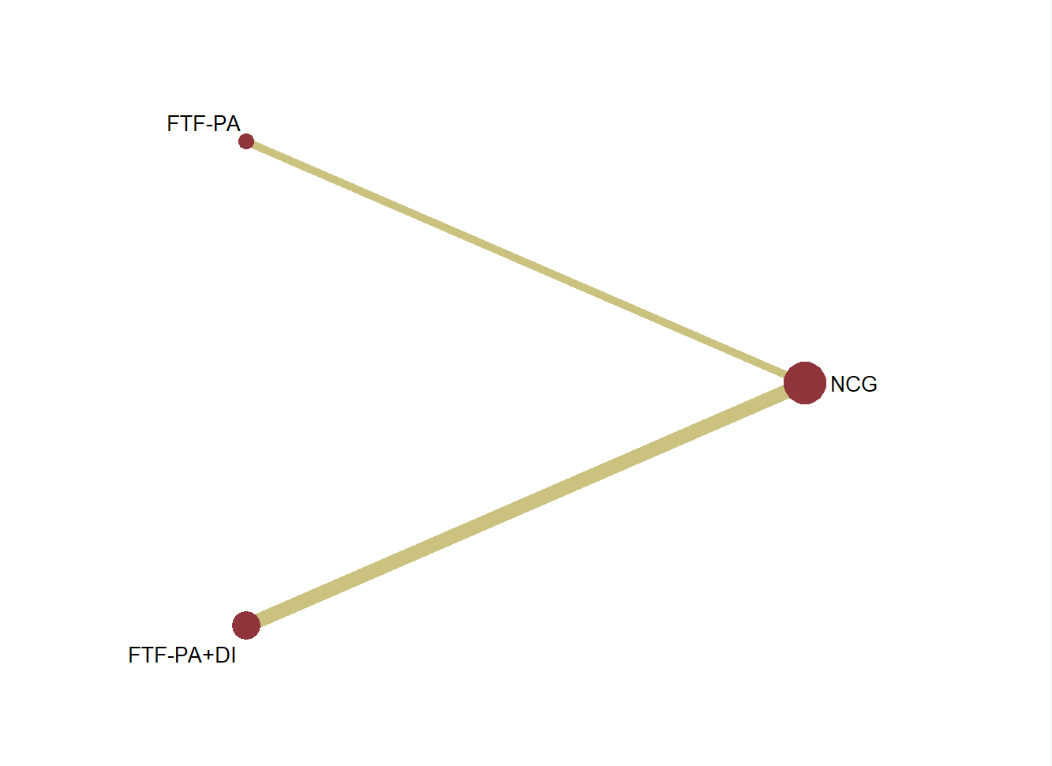
Figure. S20 Network evidence of sensitivity analysis based on the outcome of BMI Z-score(24 months).**

DI, Dietary intervention; FTF, Face-to-face; NCG, Named control group; PA, Physical activity.

**Figure. S21 Network evidence of sensitivity analysis based on the outcome of WC(12 months).**

**
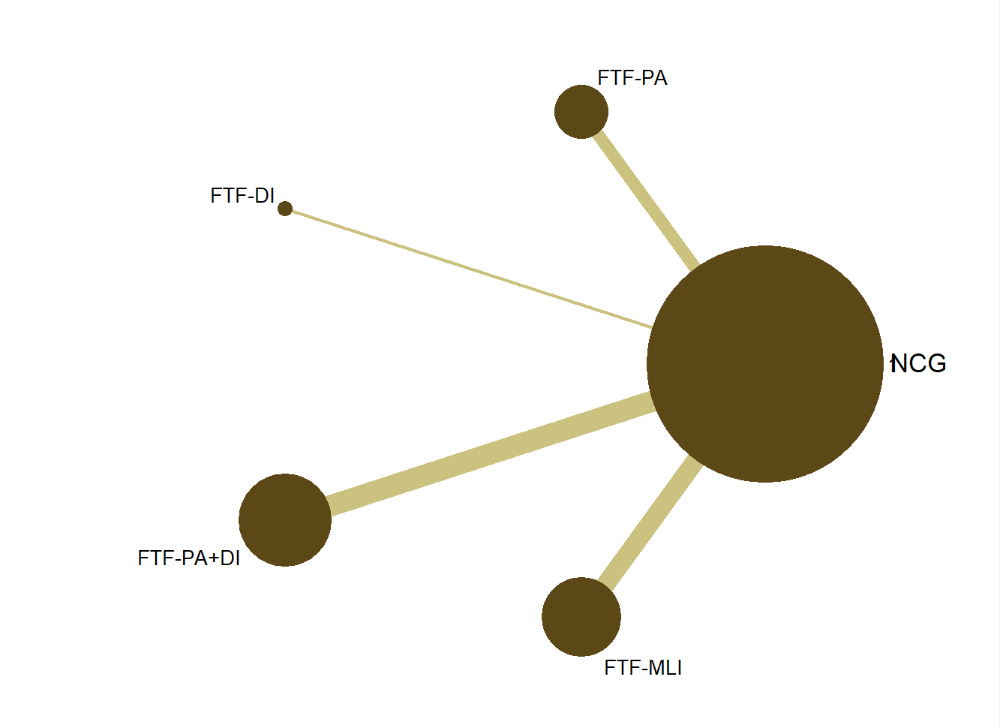
**DI, Dietary intervention; FTF, Face-to-face; MH-delivered, Mobile health-based; MLI, Muti-lifestyle intervention; NCG, Named control group; PA, Physical activity.

**
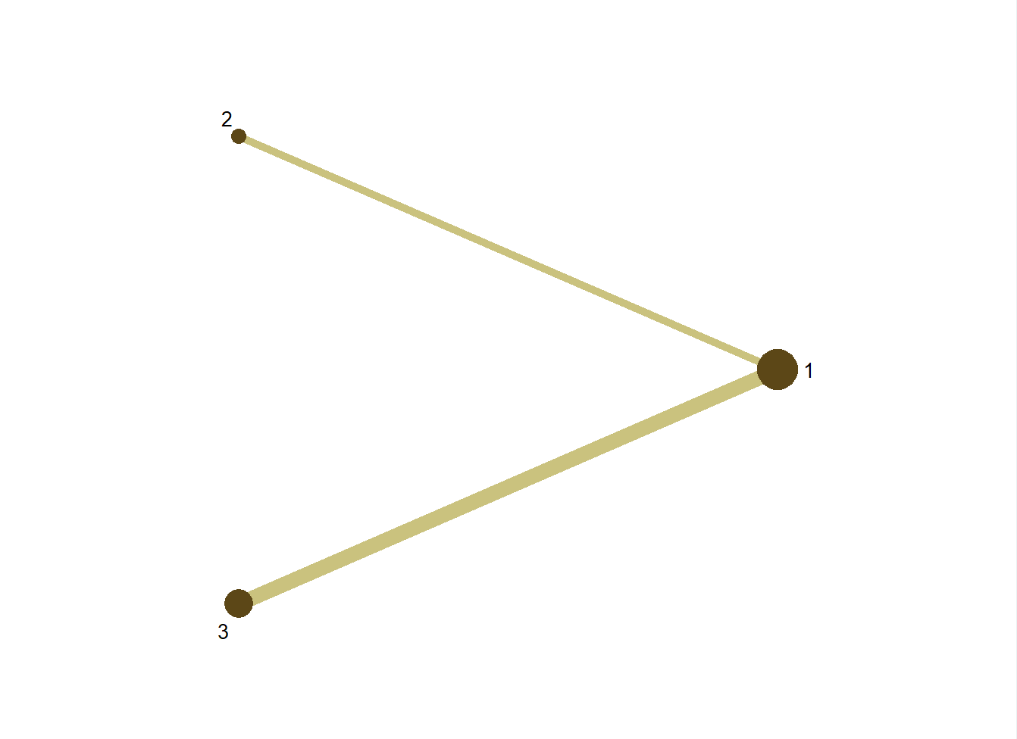
Figure. S22 Network evidence of sensitivity analysis based on the outcome of WC(24 months).**

1: NCG; 2: FTF-PA; 3:FTF-PA+DI; DI, Dietary intervention; FTF, Face-to-face; MH-delivered, Mobile health-based; MLI, Muti-lifestyle intervention; NCG, Named control group; PA, Physical activity.

**Figure. S23 Network evidence of sensitivity analysis based on the outcome of PBF(12 months).**

**
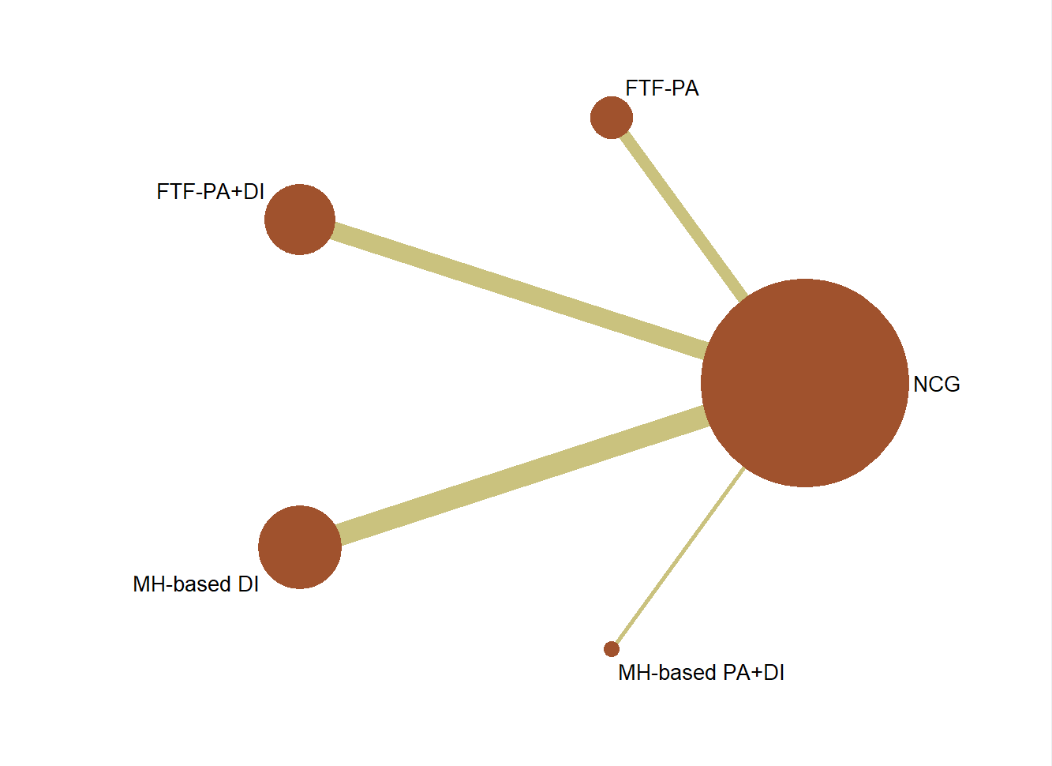
**DI, Dietary intervention; FTF, Face-to-face; MH-delivered, Mobile health-based; MLI, Muti-lifestyle intervention; NCG, Named control group; PA, Physical activity.

**
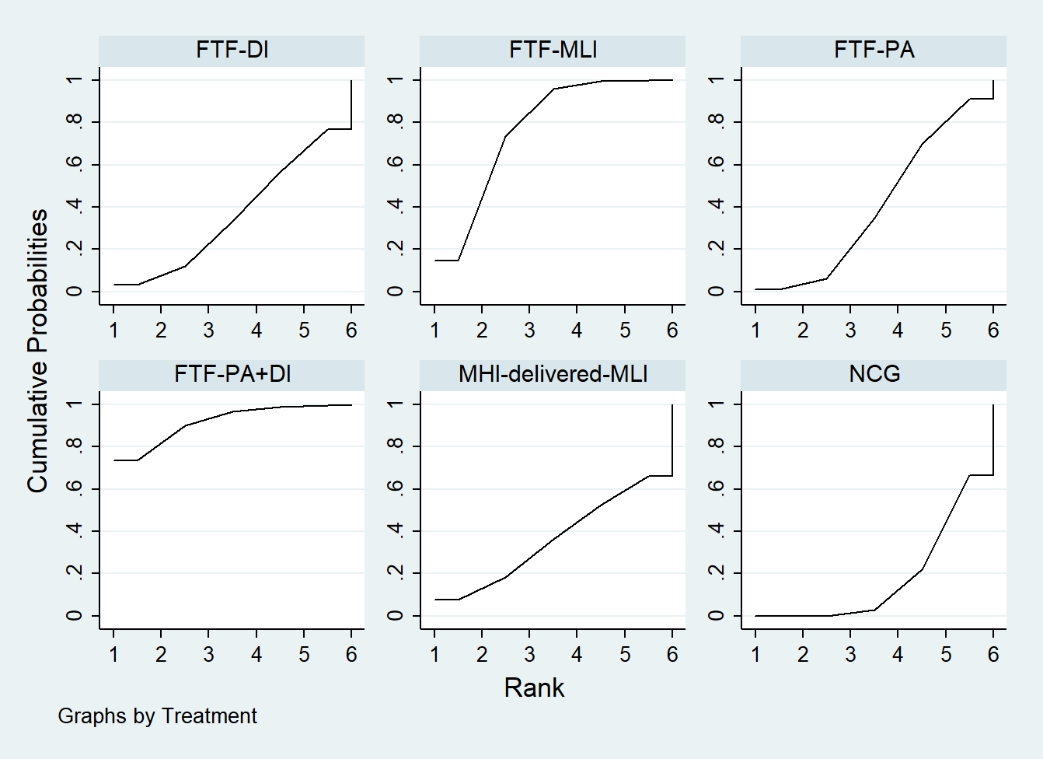
Figure. S24 SUCRA plot of sensitivity analysis based on the BMI outcome(12 months).**

DI, Dietary intervention; FTF, Face-to-face; MH-delivered, Mobile health-based; MLI, Muti-lifestyle intervention; NCG, Named control group; PA, Physical activity.

**
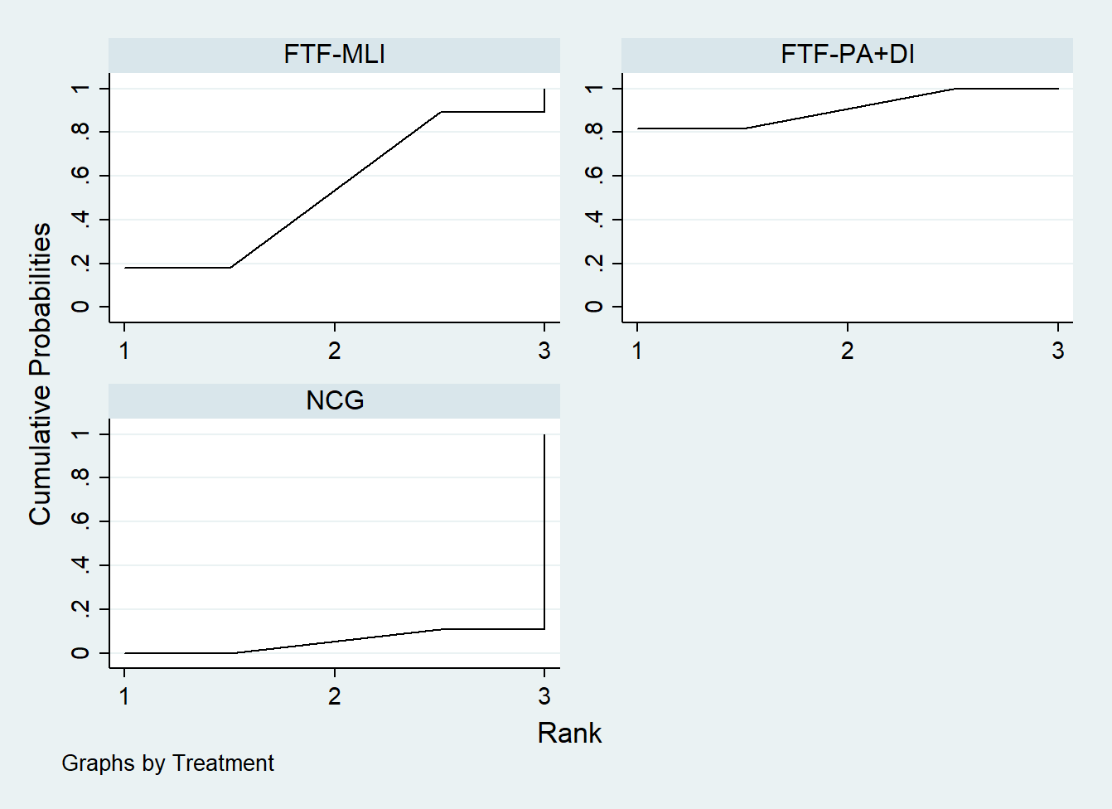
Figure. S25 SUCRA plot of sensitivity analysis based on the BMI outcome(24 months).**

DI, Dietary intervention; FTF, Face-to-face; MLI, Muti-lifestyle intervention; NCG, Named control group; PA, Physical activity.

**
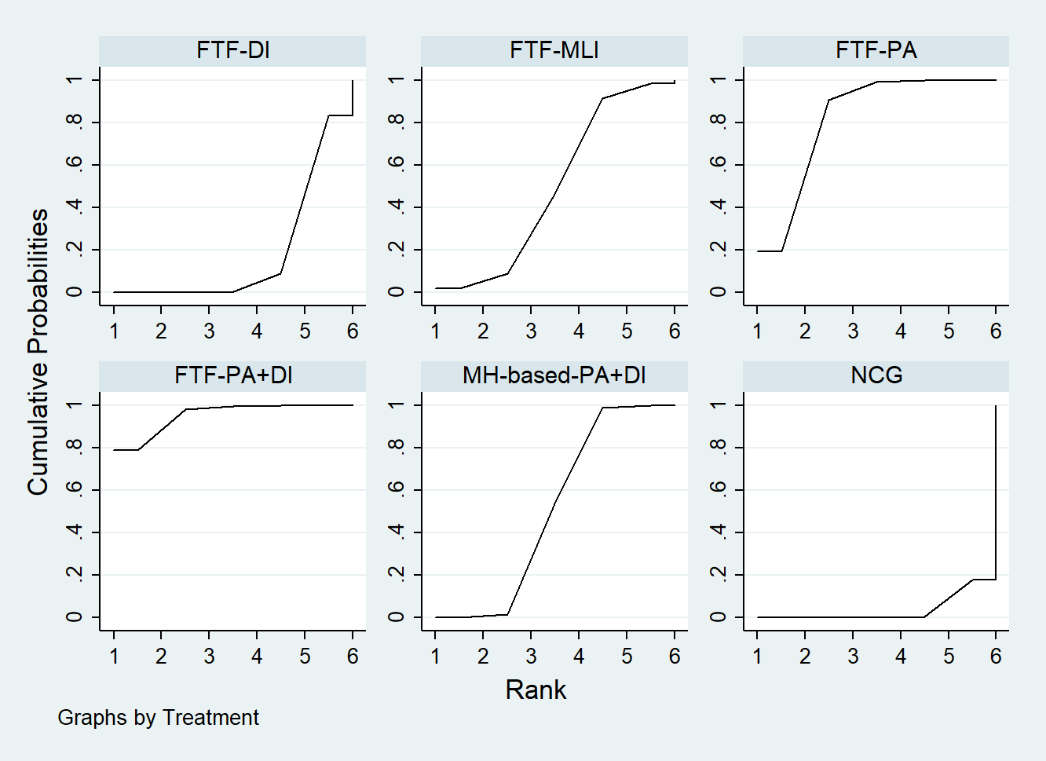
Figure. S26 SUCRA plot of sensitivity analysis based on the BMI Z-score outcome(12 months).**

DI, Dietary intervention; FTF, Face-to-face; MH-delivered, Mobile health-based; MLI, Muti-lifestyle intervention; NCG, Named control group; PA, Physical activity.

**
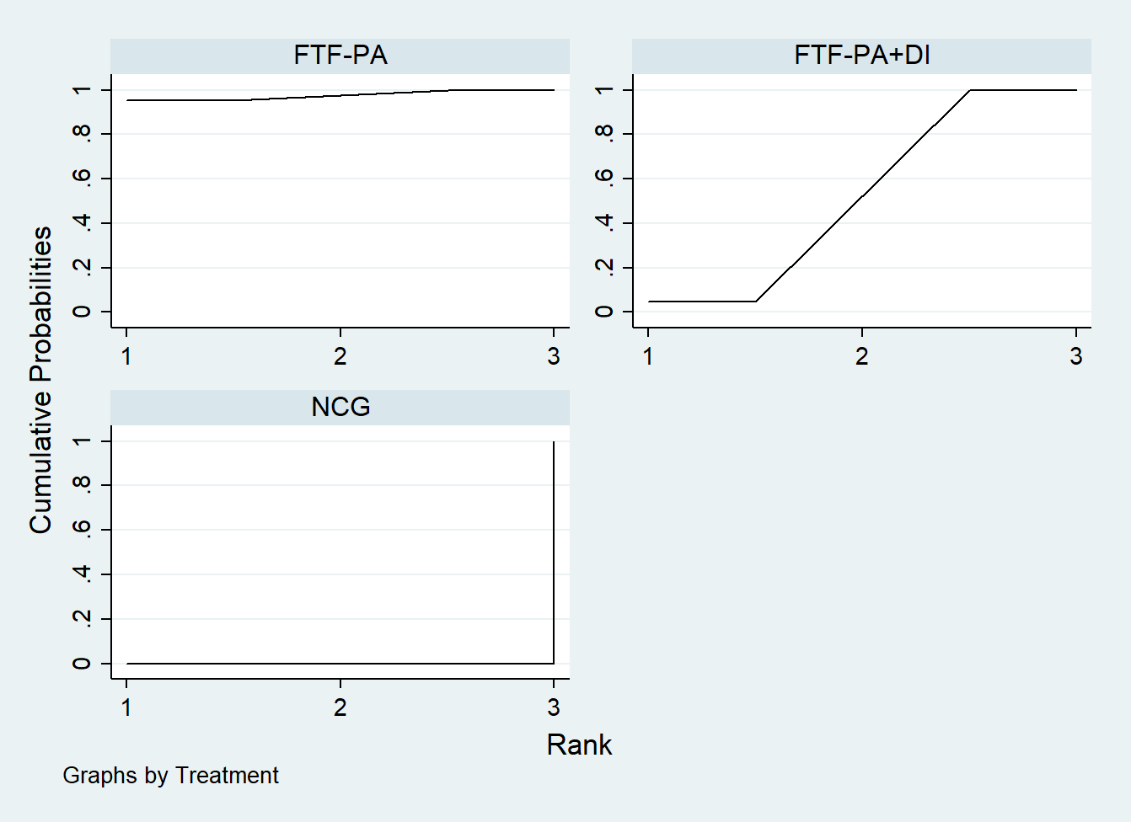
Figure. S27 SUCRA plot of sensitivity analysis based on the BMI Z-score outcome(24 months).**

DI, Dietary intervention; FTF, Face-to-face; NCG, Named control group; PA, Physical activity.

**
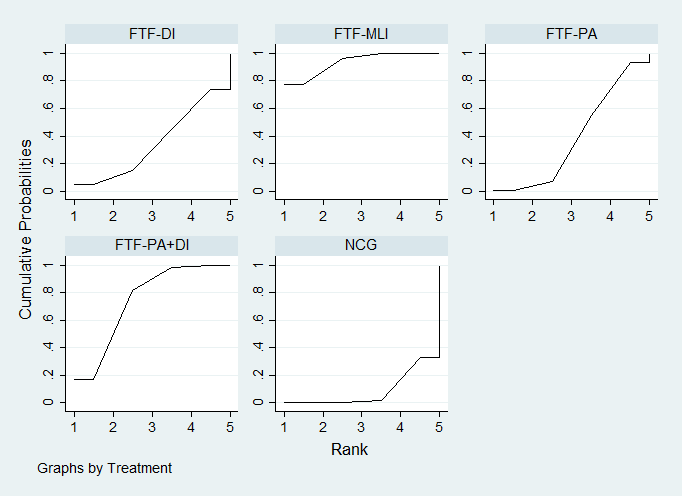
Figure. S28 SUCRA plot of sensitivity analysis based on the WC outcome(12 months).**

DI, Dietary intervention; FTF, Face-to-face; MLI, Muti-lifestyle intervention; NCG, Named control group; PA, Physical activity.

**
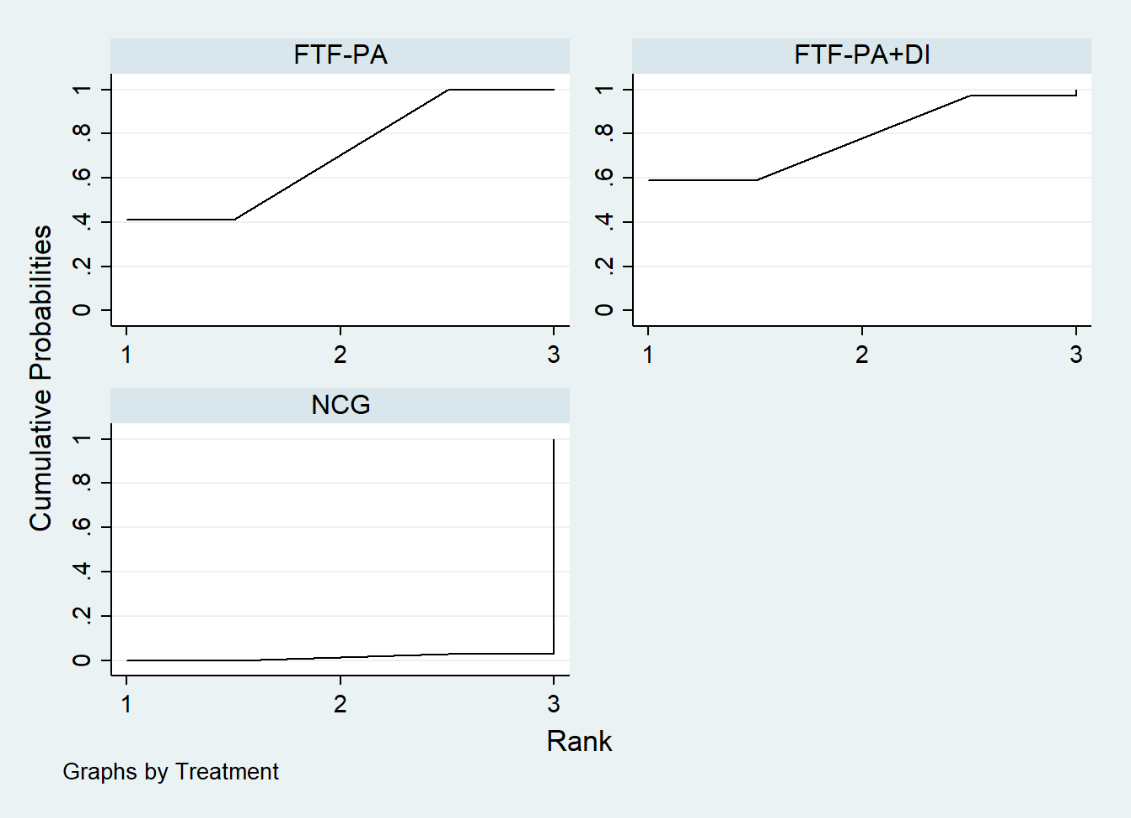
Figure. S29 SUCRA plot of sensitivity analysis based on the WC outcome(24 months).**

DI, Dietary intervention; FTF, Face-to-face; NCG, Named control group; PA, Physical activity.


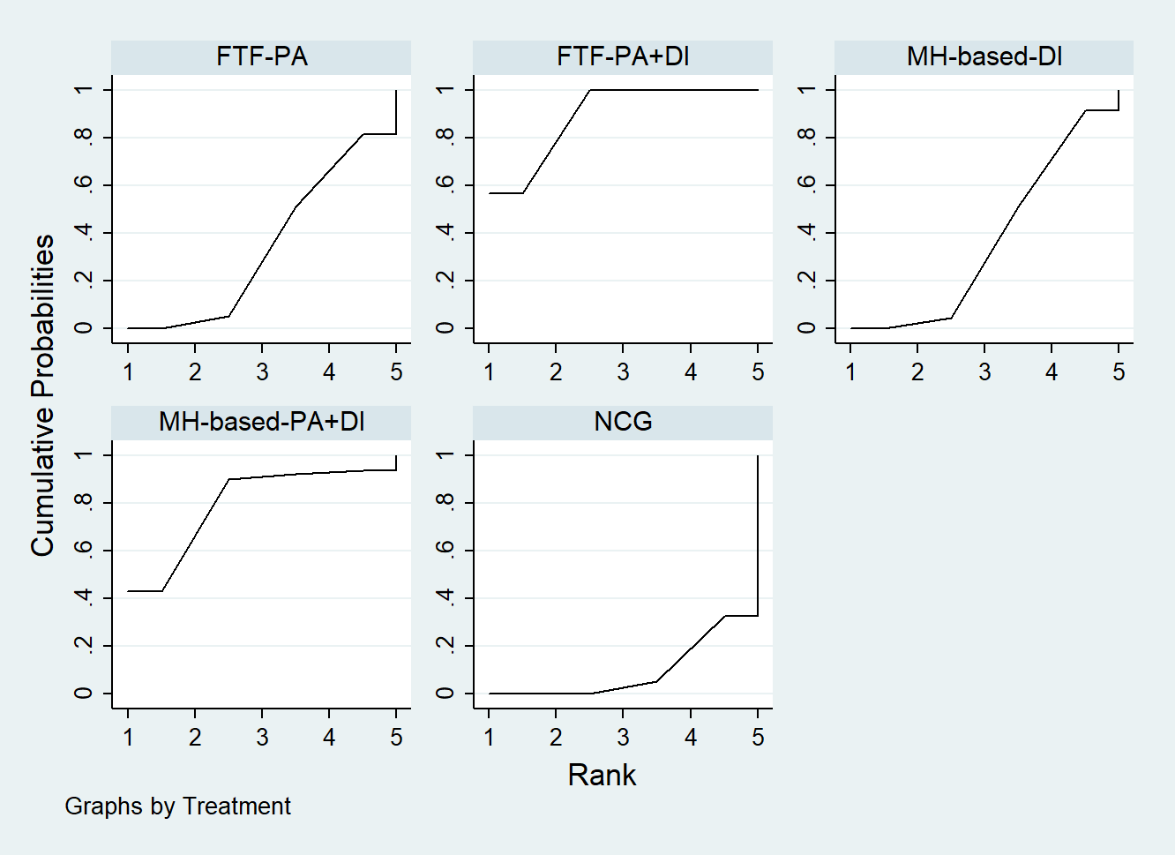
**Figure. S30 SUCRA plot of sensitivity analysis based on the PBF outcome(12 months).**

DI, Dietary intervention; FTF, Face-to-face; MH-delivered, Mobile health-based; MLI, Muti-lifestyle intervention; NCG, Named control group; PA, Physical activity.

**
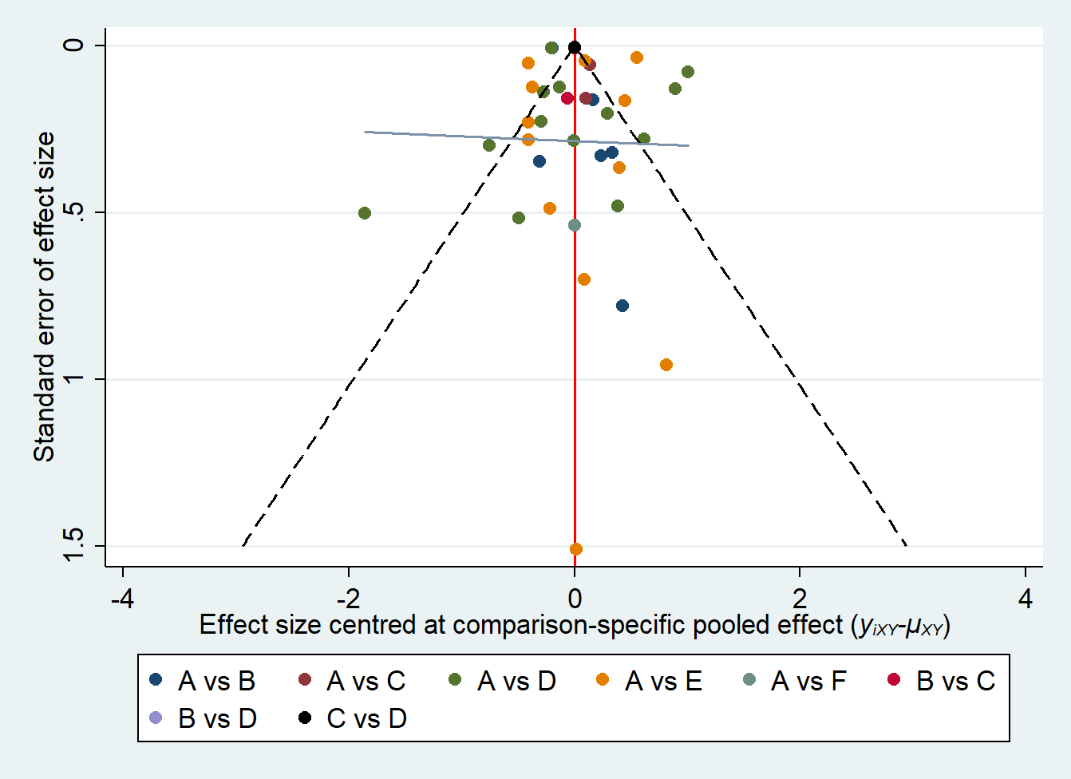
Figure. S31 Funnel plot of sensitivity analysis based on the BMI outcome(12 months).**

A: NCG, Control group(Treatment as usual, wait-list); B: FTF-PA; C: FTF-DI; D: FTF-PA+DI; E: FTF-MLI; F: MH-delivered MLI; DI, Dietary intervention; FTF, Face-to-face; MH-delivered, Mobile health-based; MLI, Muti-lifestyle intervention; NCG, Named control group; PA, Physical activity.

**
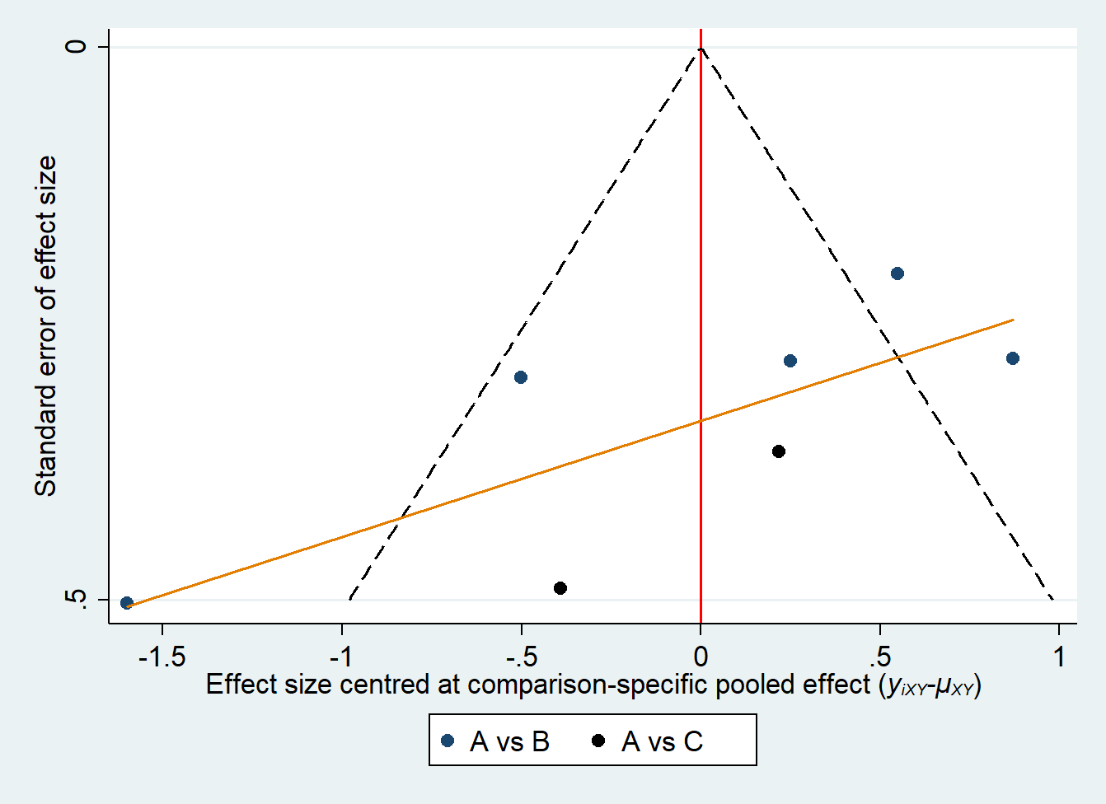
Figure. S32 Funnel plot of sensitivity analysis based on the BMI outcome(24 months).**

A: NCG, Control group(Treatment as usual, wait-list); B: FTF-PA+DI; C: FTF-MLI; DI, Dietary intervention; FTF, Face-to-face; MLI, Muti-lifestyle intervention; NCG, Named control group; PA, Physical activity.

**
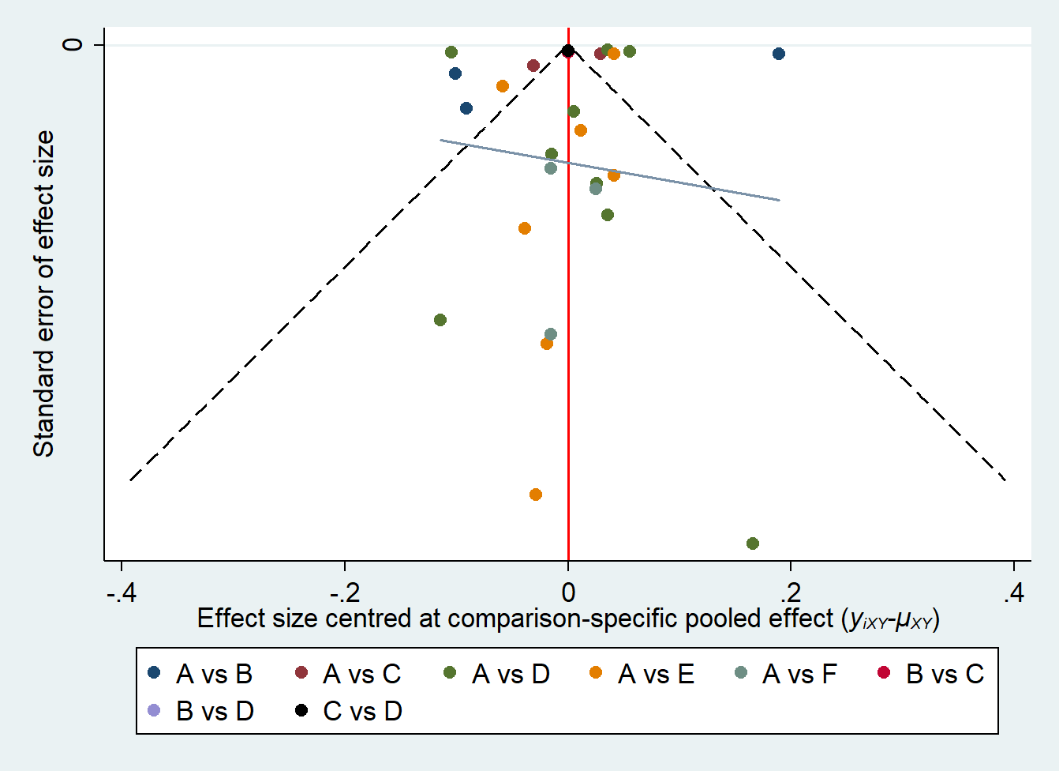
Figure. S33 Funnel plot of sensitivity analysis based on the BMI Z-score outcome(12 months).**

A: NCG, Control group(Treatment as usual, wait-list); B: FTF-PA; C: FTF-DI; D: FTF-PA+DI; E: MH-delivered PA+DI; F: FTF-MLI; DI, Dietary intervention; FTF, Face-to-face; MH-delivered, Mobile health-based; MLI, Muti-lifestyle intervention; NCG, Named control group; PA, Physical activity.

**
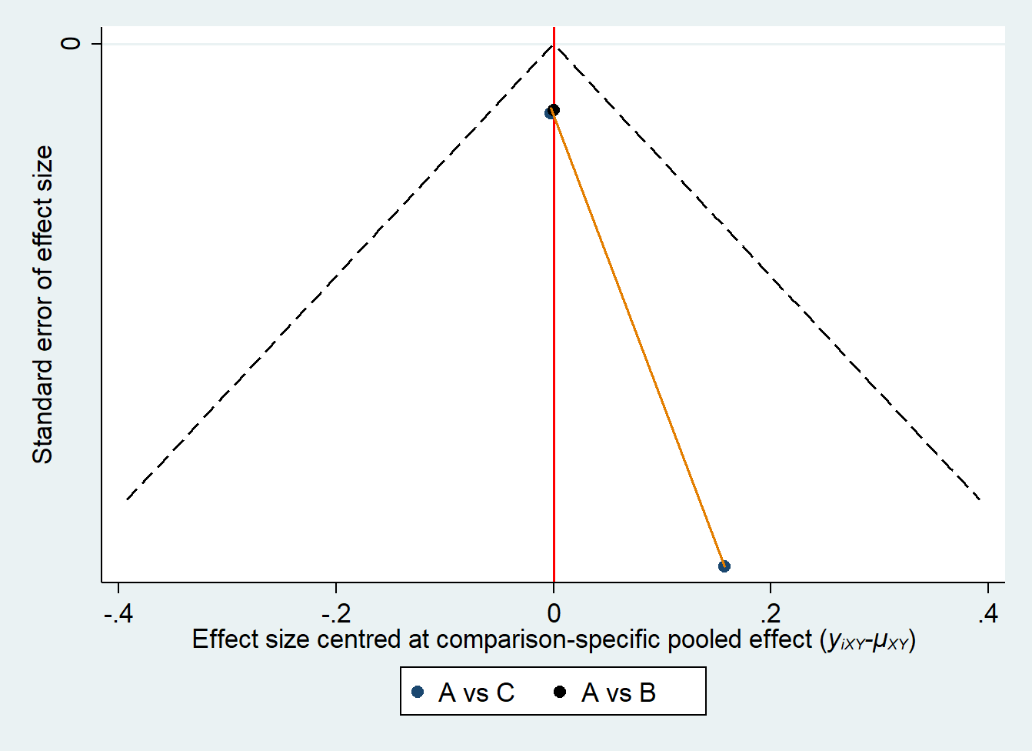
Figure. S34 Funnel plot of sensitivity analysis based on the BMI Z-score outcome(24 months).**

A: NCG, Control group(Treatment as usual, wait-list); B: FTF-PA; C: FTF-PA+DI; DI, Dietary intervention; FTF, Face-to-face; NCG, Named control group; PA, Physical activity.

**
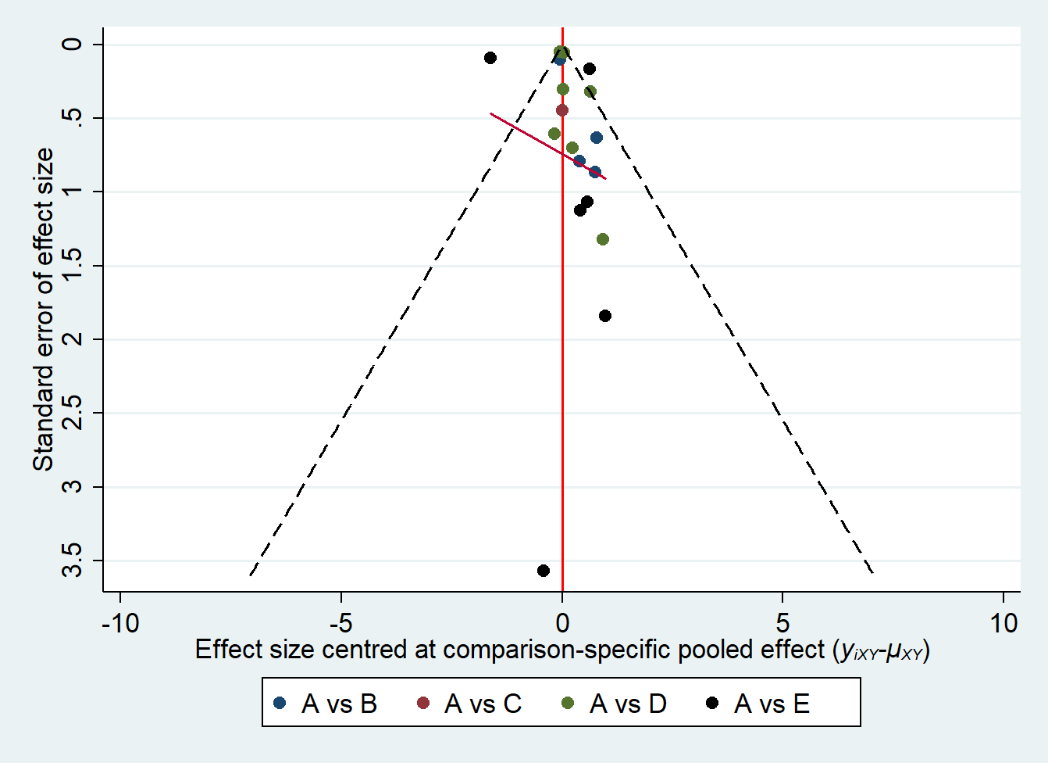
Figure. S35 Funnel plot of sensitivity analysis based on the WC outcome(12 months).**

A: NCG, Control group(Treatment as usual, wait-list); B: FTF-PA; C: FTF-DI; D: FTF-PA+DI; E: FTF-MLI; DI, Dietary intervention; FTF, Face-to-face; MLI, Muti-lifestyle intervention; NCG, Named control group; PA, Physical activity.

**
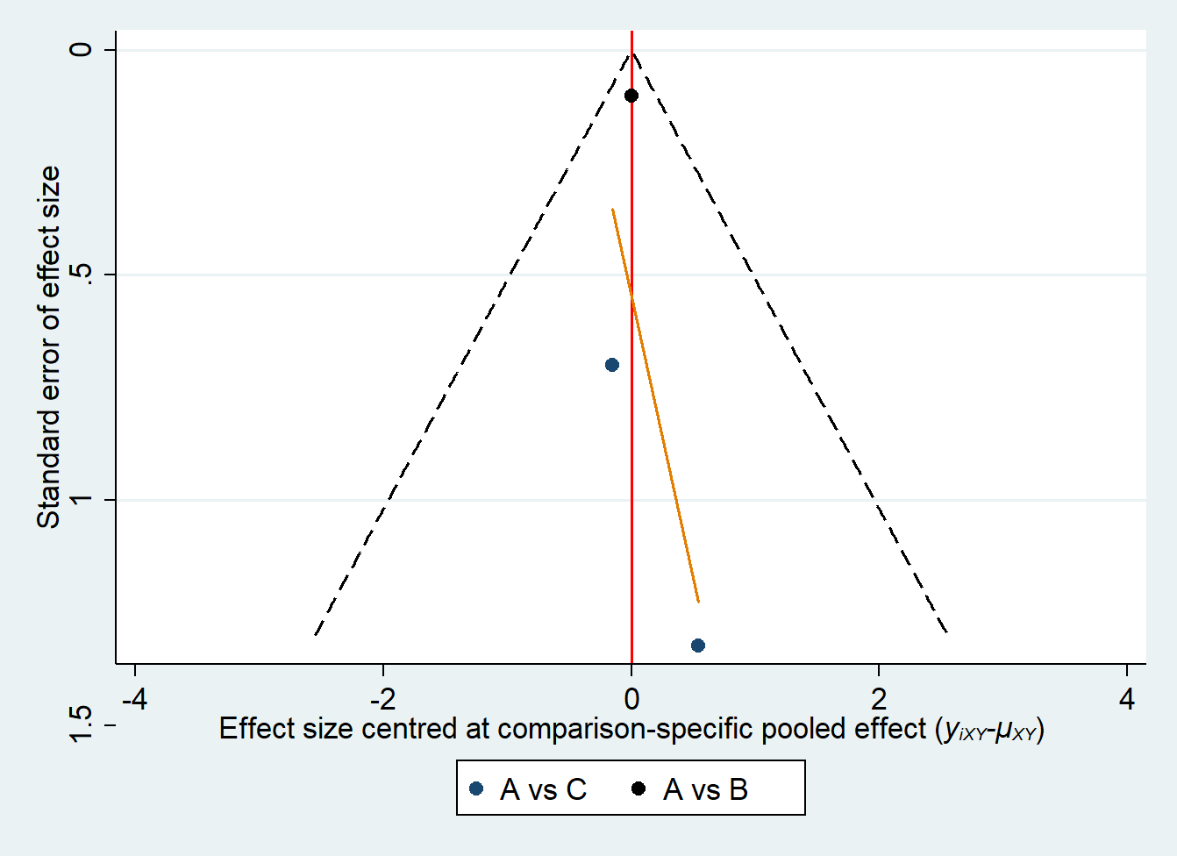
Figure. S36 Funnel plot of sensitivity analysis based on the WC outcome(24 months).**

A: NCG, Control group(Treatment as usual, wait-list); B: FTF-PA; C: FTF-PA+DI; DI; FTF, Face-to-face; NCG, Named control group; PA, Physical activity.

**
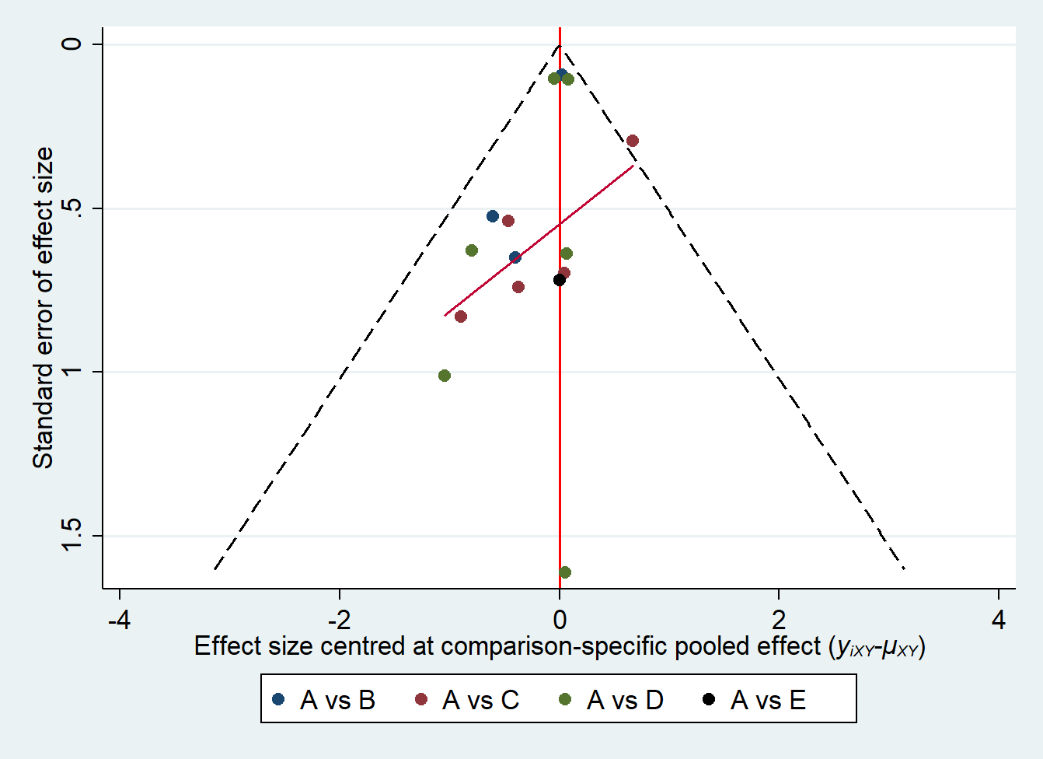
Figure. S37 Funnel plot of sensitivity analysis based on the PBF outcome(12 months).**

A: NCG, Control group(Treatment as usual, wait-list); B: FTF-PA; C: FTF-PA+DI; D: MH-delivered DI; F: MH-delivered PA+DI; DI, Dietary intervention; FTF, Face-to-face; MH-delivered, Mobile health-based; NCG, Named control group; PA, Physical activity.
